# A novel and sensitive DNA methylation marker for the urine-based liquid biopsies to detect bladder cancer

**DOI:** 10.1186/s12885-022-09616-y

**Published:** 2022-05-06

**Authors:** Leihong Deng, Haichao Chao, Huanhuan Deng, Zhaojun Yu, Rongsong Zhao, Longwu Huang, Yun Gong, Yueting Zhu, Qingping Wang, Feng Li, Lirong Liu, Lei He, Zhimin Tang, Caizhi Liao, Yan Qi, Xianshu Wang, Tao Zeng, Hongzhi Zou

**Affiliations:** 1 The First Affiliated Hospital of Nanchang University, No. 1 Minde Road, Nanchang, 330006 Jiangxi China; 2 The Second Affiliated Hospital of Nanchang University, No. 1, Minde Road, Nanchang, 330006 Jiangxi China; 3 Donghu Campus, Medical College of Nanchang University, 461 Bayi Dadao, Nanchang, 330006 Jiangxi China; 4 Creative Biosciences (Guangzhou) CO., Ltd, Guangzhou, 510530 Guangdong China

**Keywords:** Bladder cancer (BC), Urine-based DNA (uDNA) test, Methylation biomarker, Sensitivity, Specificity

## Abstract

**Background:**

Better prognostic outcome is closely correlated with early detection of bladder cancer. Current non-invasive urianalysis relies on simultaneously testing multiple methylation markers to achieve relatively high accuracy. Therefore, we have developed an easy-to-use, convenient, and accurate single-target urine-based DNA methylation test for the malignancy.

**Methods:**

By analyzing TCGA data, 344 candidate markers with 424 primer pairs and probe sets synthesized were systematically screened in cancer cell lines, paired tissue specimens, and urine sediments from bladder cancer patients and normal controls. The identified marker was further validated in large case-control cohorts. Wilcoxon rank sum tests and c2 tests were performed to compare methylation levels between case-control groups and correlate methylation levels with demographic and clinical characteristics. In addition, MSP, qMSP, RT-PCR, western blot analysis, and immunohistochemistry were performed to measure levels of DNA methylation, mRNA transcription, and protein expression in cancer cell lines and tissues.

**Results:**

A top-performing *DMRTA2* marker identified was tested in both discovery and validation sets, showing similar sensitivity and specificity for bladder cancer detection. Overall sensitivity in the aggregate set was 82.9%(179/216). The specificity, from a control group consisting of patients with lithangiuria, prostatoplasia, and prostatitis, is 92.5%(468/506). Notably, the methylation assay had the highest sensitivities for tumors at stages of T1(90.4%) and T2(95.0%) compared with Ta (63.0%), T3(81.8%), and T4(81.8%). Furthermore, the test showed admirable detection rate of 80.0%(24/30) for recurring cancers. While methylation was observed in 39/54(72.2%) urine samples from patients with carcinomas of renal pelvis and ureter, it was detected at extremely low rate of 6.0%(8/133) in kidney and prostate cancers. Compared with SV-HUC-1, the normal bladder epithelial cell line, *DMRTA2* was hypermethylated in 8/9 bladder cancer cell lines, consistent with the results of MSP and qMSP, but not correlated with mRNA and protein expression levels in these cell lines. Similarly, DMRTA2 immunostaining was moderate in some tissues but weak in others. Further studies are needed to address functional implications of *DMRTA2* hypermethylation.

**Conclusions:**

Our data demonstrated that a single-target DNA methylation signature, m*DMRTA2*, could be highly effective to detect both primary and recurring bladder cancer via urine samples.

**Supplementary Information:**

The online version contains supplementary material available at 10.1186/s12885-022-09616-y.

## Introduction

Bladder cancer (BC) is the 4th most frequently occurring malignancies and the 9th most common cause of death worldwide in men [1]. In China, about 53,000 new cases were diagnosed annually, and the incidence rate ranked 6th in men ahead of prostate cancer and had been continuously increasing [2]. The invasive and metastatic form of the cancer is the main cause of death or unfavorable prognosis for BC patients. The 5-year survival rate for patients with localized tumors can reach as high as 92%, but only 45% for those with tumors spreading to nearby regions [3]. Around 70% of the patients with superficial BC can be completely free of tumor cells after surgical resection. However, the overall 5-year survival rate did not significantly improve in the past thirty years even though numerous therapeutic approaches have been developed for its clinical treatment [4]. Therefore, it is imperative to detect BC at early stage for the therapeutic interventions to be more effective and survival rate to be further increased.

Certain urinary tests for detection of BC have been approved by the US Food and Drug Administration (FDA) for clinical practice. BladderChek (Matritech Inc., Newton, MA), an immunoassay of a nuclear matrix protein NMP22 in urine, is more sensitive than cytology in detecting low-grade and early stage BC [5]. Cytological examination of urine using fluorescence in situ hybridization (FISH) technique, UroVysion (Vysis Inc., Downers Grove, IL), can identify BC at Ta, G1, and T1 with very high specificity but poor sensitivity [5, 6]. Another adjunct test to cytology, Immunocyt™, is a immunocytological assay particularly suited for monitoring BC recurrence [7]. The common drawback of the aforementioned urianalyses is that they have low specificity and are prone to interference from benign conditions of the urinary tract. Other routine diagnostic methods including computed tomography (CT) and ultrasound are not particularly adept at detecting early stage BC [8]. Cystoscopy combined with tissue biopsy--the gold standard for diagnosing BC--can miss 10–40% of the cancer cases due to multiple factors [9, 10]. Meanwhile, the method is invasive, causing physical discomfort and psychological trauma for the patients. Furthermore, BC recurrence after surgery is frequent, and postoperative patients are recommended to undergo lifetime surveillance by cystoscopy, putting continuous physical stress and financial burden on individuals and their families [11]. Therefore, development of a non-invasive, highly sensitive, and more specific diagnostic alternative is desirable for early detection and postoperative surveillance of BC.

In recent years, a variety of novel biomarkers have been screened and identified in urine sediments for BC detection through wide-ranging technologies including MassARRAY, expression profiling, metabolomic analysis, and second-generation sequencing [12–15]. Among these various testing developments, detection of aberrantly methylated DNA in urine has gained prominence and emerged as a promising and attractive approach to aid BC diagnosis and prognosis. DNA methylation is one of the most common epigenetic alterations and plays crucial roles in early tumorigenesis [16]. Unlike recurring somatic mutations in limited numbers, large-scale DNA methylation, which is tissue- and cancer-specific, can be better suited to detect early-stage cancers [17]. Hypermethylation in promoter regions of a large number of cancer driver genes has been well characterized for bladder tumors versus normal epithelia [18, 19]. Increased methylation in most of these promoter sites occurs early in BC and is shared across all grades and stages [19]. Hence, a plethora of individual genes and gene panels have been tested on tumor tissues and urine samples for their diagnostic potential for BC mostly via methylation-specific PCR method [20, 21]. Several methylation markers or their combinations, including *ZNF154*, *POU4F2*, *EOMES*, *HOXA9*, *TWIST1*, *OTX1,* etc., demonstrated highest sensitivities in early diagnosis or recurrence surveillance of BC [20]. However, current non-invasive urianalysis for detecting BC have only been able to achieve relatively high accuracy by simultaneously testing multiple markers [22–29]. A commercial test, Bladder EpiCheck (Nucleix, Rehovot, Israel), demonstrated higher diagnostic accuracy than most other urinary tests for BC detection in multiple clinical trials, but it employed a total of 15 DNA methylation biomarkers to achieve robust performance [23, 30]. Most recently, several studies reported robust performance characteristics for urine-based tests using combination of two methylation biomarkers [12, 28, 29]. Compared to other non-invasive tests including NMP22, UroVision, and Bladder EpiCheck, the dual-marker risk prediction model seems to offer both superior sensitivity in detecting BC, in particular Ta stage cancer, and significant cost-reduction in clinical practice [29]. By contrast, based on most up-to-date publications, comprehensive evaluation of a single-target methylation test for BC risk prediction has not been reported. By analyzing TCGA data and improving analytic technology currently available, we have developed an easy-to-use, convenient, and accurate detection approach for BC relying on a single methylation marker. In current investigation, we systematically evaluated the performance indexes of the exclusive marker in terms of its sensitivity, specificity, AUC values in a large number of urine samples from BC patients, healthy donors, and control individuals with benign conditions of urinary tract. In addition, we also assessed its detection capacity for other types of urothelial carcinomas and the effect of interference diseases such as prostate and kidney cancers on its performance in detecting BC. Finally, we examined its levels of mRNA and protein expression in BC cell lines and tumor tissues.

## Materials and methods

### BC cell lines

Seven BC cell lines, BIU-87, SW780, T24, 5637, SCaBER, TCCSUP, and J82, were used to test primer sets for candidate genes by methylight method. Two additional BC cell lines, UM-UC-3 and RT4, were also included for subsequent measurement of DNA methylation levels and quantitative RT-PCR experiments. SV-HUC-1, a bladder epithelial cell line, was used as a normal control.

### Sample collection

The study was approved by the Institutional Review Board of the Second Affiliated Hospital of Nanchang University ([2018] No (027)) and performed to Helsinke Declaration. Additionally, written informed consents were obtained for all participants. All samples including paraffin-embedded blocks, fresh frozen tissues, and urine specimens were collected from May, 2018 to February, 2021. Frozen tissues were stored at − 80 °C until use. All cancer tissue specimens were reviewed by an experienced pathologist, and all cancers were classified according to the 7th edition of American Joint Committee on Cancer (AJCC).

### Microdissection and DNA extraction

BC tissue sections were examined by an experienced pathologist who circled out histologically distinct lesions with more than 70% tumor cells to direct careful microdissection. Different types of DNA were extracted using QIAamp DNA Mini Kit (Qiagen, Hilden, Germany) according to the manufacturer’s instruction.

### Bisulfite treatment

DNA was treated with bisulfite using EZ DNA Methylation Kit (Zymo Research, Irvine, CA) according to the manufacturer’s instructions. For cell line and tissue DNA samples, approximately 500 ng genomic DNA was added into the bisulfite treatment reaction and eluted out in 20 μL M-Elution Buffer. For urine DNA samples, 0–400 ng extracted DNA was bisulfite-treated and eluted out in 100 μL TE buffer.

### Methylation-specific PCR (MSP)

MSP was performed to determine the methylation status of *DMRTA2* in BC cell lines. Methylated and unmethylated *DMRTA2* (m*DMRTA2* and um*DMRTA2*) primers were designed in its CpG islands. Briefly, 1 μL bisulfite-treated DNA was amplified in a total volume of 25 μL containing 2 × iTaq Universal SYBR@ Green Supermix (Bio-Rad, Hercules, CA) and 100 nmol/L of each primer. Amplification included hot-start at 95 °C for 5 minutes, denaturing at 95 °C for 30s, annealing at 60 °C for 30s, extension at 72 °C for 30s for 35 cycles, and a final 5 minutes extension step at 72 °C [31]. Bisulfite treated human genomic DNA (Merck Milipore, Darmstadt, Germany) and CpGenome Universal Methylated DNA (Sigma-Aldrich, St. Louis, MO) were used as unmethylation and methylation controls, respectively. Water was used as no template control. All MSP products were verified by 2% agarose gel electrophoresis.

### Real-time quantitative methylation-specific PCR (qMSP)

An improved methylight assay was performed for bisulfite-treated DNA [32]. The sequences of primers and TaqMan probes designed for m*DMRTA2* as well as *ACTB* were included in Supplementary Table S1. *ACTB* was included as a reference gene to assess the quality of isolated DNA. The qMSPs for urine samples and BC cancer cell lines were conducted as previously described [31]. Briefly, the total volume of each reaction was 30 μL, amplified via 95 °C, 5 min followed by 45 cycles of 95 °C, 15 s, 58 °C, 30s, and 72 °C, 30s and a final step at 40 °C for 30s on Roche LightCycler 480 II (Roche, Basel, Switzerland) [31]. The probes used for qMSP of *CHAD*, *MEIS1*, *CMTM2*, *DRD4*, *PENK*, and *DMRTA2* are CGGTTGCGGTTAGGGTTATCGTAT, CGAGAGGGGTCGGGCGAGTTAG, CGTTGCGTTCGCGGAGTTTAGG, CGTGA GTTTGGCGGTCGTCGATTT, CGAACCAAACTACGAAACTCTAAACGCC, and CTATTACCGCCGCCGCCGTCG, respectively.

### Interpretation and data analysis of real-time qMSP of *DMRTA2*

Abs Quant/2nd Derivative Max method in Roche LightCycler 480 II (Roche, Basel, Switzerland) was used to calculate cycling threshold (CT value) by assigning a prespecified cut-off value for each amplification curve as previously reported [33]. Every batch of PCR reactions were performed with three controls, an *ACTB* internal control, m*DMRTA2* as a positive control, and um*DMRTA2* as a negative control. If a sample showed no amplification of m*DMRTA2*, no CT value would be assigned for the sample. All valid samples should satisfy the requirement of CT value of *ACTB* ≤ 35. If a sample has CT value of *ACTB* > 35, the result would be considered invalid. Target gene capture, bisulfite treatment, and PCR amplification would be rerun using a second aliquot from the sample. The CT threshold of 37 was selected to dichotomize the result of qMSP for m*DMRTA2* mainly to maximize sensitivity and minimize false positive rate. Therefore, urine samples with CT values ≤37 for m*DMRTA2* were called “positive” and were most likely associated with BCs. In contrast, urine samples with CT value > 37 or no CT value assigned were reported negative and were unlikely associated with bladder neoplasia. All negative samples without CT values assigned from Roche LightCycler 480 II would be arbitrarily given a value of 43 each in order to compare m*DMRTA2* levels between BCs and normal controls.

### 5-aza-2′-deoxycytidine treatment

To assess the impact of methylation on the expression of *DMRTA2* gene, demethylation agent 5-aza-2′-deoxycytidine (5-Aza-dC, Sigma, St. Louis, MO) was used to treat all nine BC cell lines and one normal cell line as reported previously [34]. Treat the cells with 10 μM 5-aza for 6 consecutive days, change the medium every day. The mRNA expression of *DMRTA2* in cell lines was quantified with RT-PCR. *GAPDH* was used as an internal reference gene to normalize cDNA input. The RT-PCR primers of *GAPDH* and *DMRTA2* are F2: GGAAGGTGAAGGTCGG AGTCA; R2: GTCATTGATGGCAACAATATCCACT; F7: CAGACAGGTGCAGGT GTTCT; R7: TCCCAGCCTTTTGGAAAGGG.

### IHC and western blot

IHC was used to detect DMRTA2 expression in tissues. Tissue sections of normal bladder and bladder tumor were used. The procedure was conducted as previously reported [35]. The commercially available antibodies DMRTA2 (PA5–60237) in 1:20 dilution were used to stain sections. The intensity of the specific immunohistochemical staining reactions were evaluated using a semi-quantitative method (IRS-score), as previously described [36]. H&E staining was carried out using a Hematoxylin and Eosin Staining Kit (Beyotime, Shanghai, China) according to the manufacturer’s protocol. Western blot analysis was also conducted to detect *DMRTA2* protein expression in cell lines. Total protein was extracted, electrophoresed, and transferred to polyvinylidene fluoride membranes. Membranes were incubated with DMRTA2 and GAPDH primary antibodies (Invitrogen, Carlsbad, CA) and then with appropriate HRP-conjugated secondary antibodies (Invitrogen, Carlsbad, CA). Fluorescent signals were detected with ChemiDoc™ Imaging System (Bio-Rad, Hercules, CA).

### RNA extraction and RT-PCR

Total RNA was isolated using TRIzol™ Reagent (Invitrogen, Carlsbad, CA) from various cell lines. First-strand cDNA was synthesized using the ReverAid First Strand cDNA Synthesis Kit (Thermo Fisher Scientific, Waltham, MA). Real Time-PCR (RT-PCR) was performed with Applied Biosystems ABI 7500. *GAPDH* was used as an internal control. Primer sequences are shown in Supplementary Table S2.

### Statistical analysis

Wilcoxon rank sum tests were performed to compare methylation levels between different types of sample groups. Paired t test was used in paired samples. c2 test was applied to evaluate the correlation of methylation levels with demographic and clinical characteristics, such as age, sex, tumor-node-metastasis (TNM) stage, tumor location, tumor size, and dysplasia. ROC curve was constructed to compare *DMRTA2* methylation levels between sample types. The associated AUC value was calculated for each ROC curve. All experiments examining levels of methylation, mRNA and protein expression in BC cell lines were independently performed at least three times. Data is shown as mean ± SD, with the significance between the means calculated using Two-tailed Student’s t-test. A *p* value less than 0.05 was considered statistically different (* *p* < 0.05, ** *p* < 0.01). Statistical analyses were conducted with GraphPad Prism Version 5.0 (Graph Pad Software Inc. San Diego, CA).

## Results

### Screening for best-performing methylation biomarkers in urine specimens

**Fig. 1:**
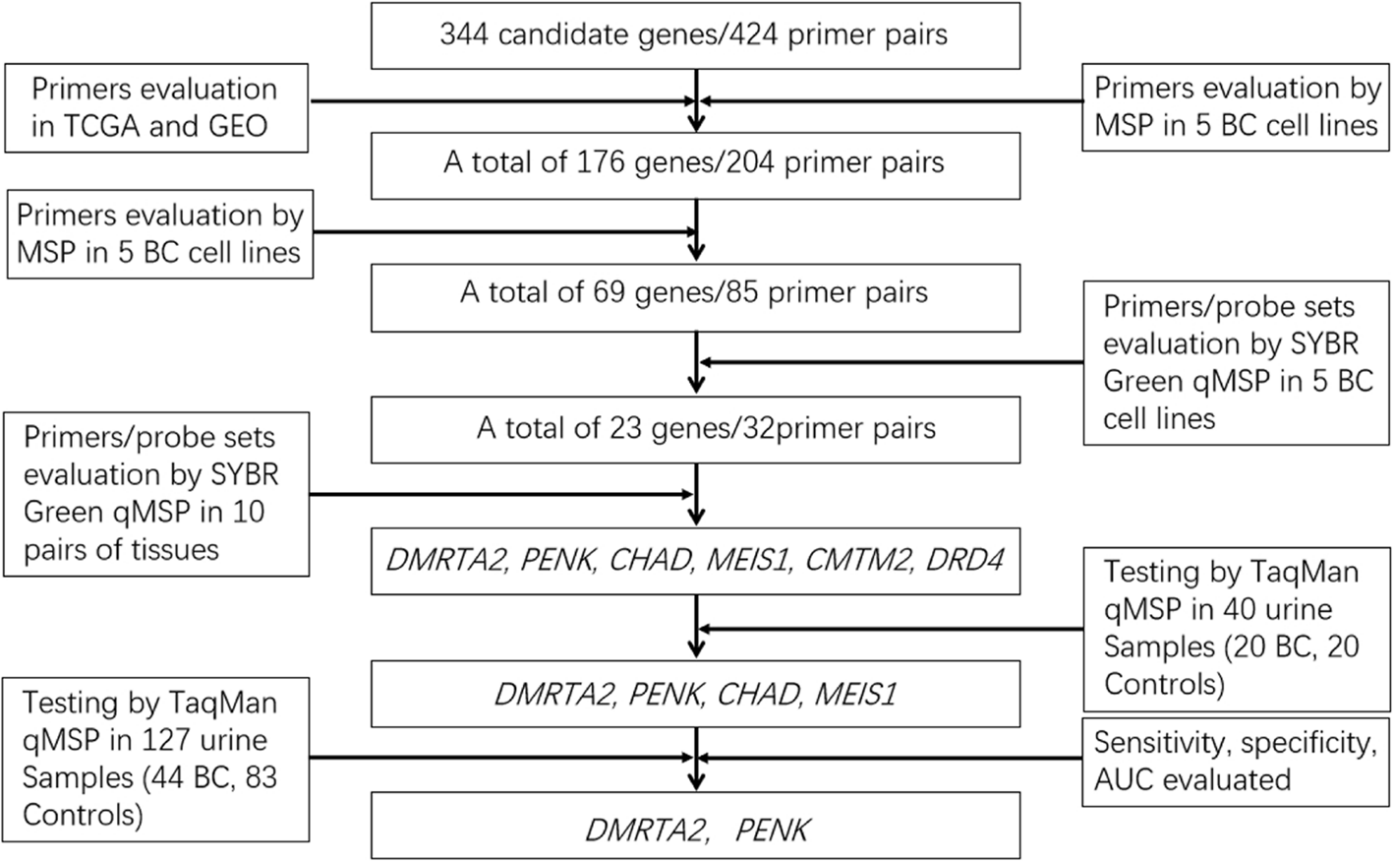
Diagram of work-flow to screen for top-two urine biomarkers, m*DMRTA2* and m*PENK*, for BC detection

In the current investigation, we initially searched literature covering a wide variety of cancers and conducted differential methylation analysis of Illumina’s HM450K data from 21 pairs of BC and matched normal tissue specimens in TCGA as well as methylation data of 20 BC cell lines available in GEO (GSE68379) to select 344 candidate markers and design 424 primer pairs for MSP assays of a plethora of methylation sites (Supplementary Information and Supplementary Table S1). The panel was subsequently whittled down to 176 genes and 204 primer pairs based on first-round MSP results for genes obtained from literature search in 4 BC cell lines including 5637, SW780, T24, and TCCSUP and one immortalized epithelial cell line SV-HUC-1. All 204 selected primer pairs were further tested via second-round MSP assays in the aforementioned cell lines (Supplementary Table S2). A total of 69 genes with detectable methylation in at least 3 out of 4 BC lines were further selected for quantitative assessment of their methylation levels via SYBR green qMSP (Supplementary Table S2-S3). Using the same quantifying method, the group was further narrowed down to a panel of 23 genes whose methylation was further quantified in 10 pairs of BC and normal tissue specimens (Supplementary Table S4). The top 6 genes including *CHAD*, *MEIS1*, *DMRTA2*, *PENK*, *CMTM2*, *DRD4* with highest sensitivity and specificity were selected for subsequent examination in 40 urine samples from 20 BC patients and 20 controls by TaqMan probe-based qMSP (Supplementary Table S5). The top 4 candidate genes were tested again in a total of 127 urine samples including 44 BC patients and 83 normal controls. Finally, two best markers, *DMRTA2* and *PENK*, were subjected to the final round of screening in 237 urine samples including 100 BC patients and 137 controls to further evaluate their performance (Fig. 1, Supplementary Table S6).

**Fig. 2:**
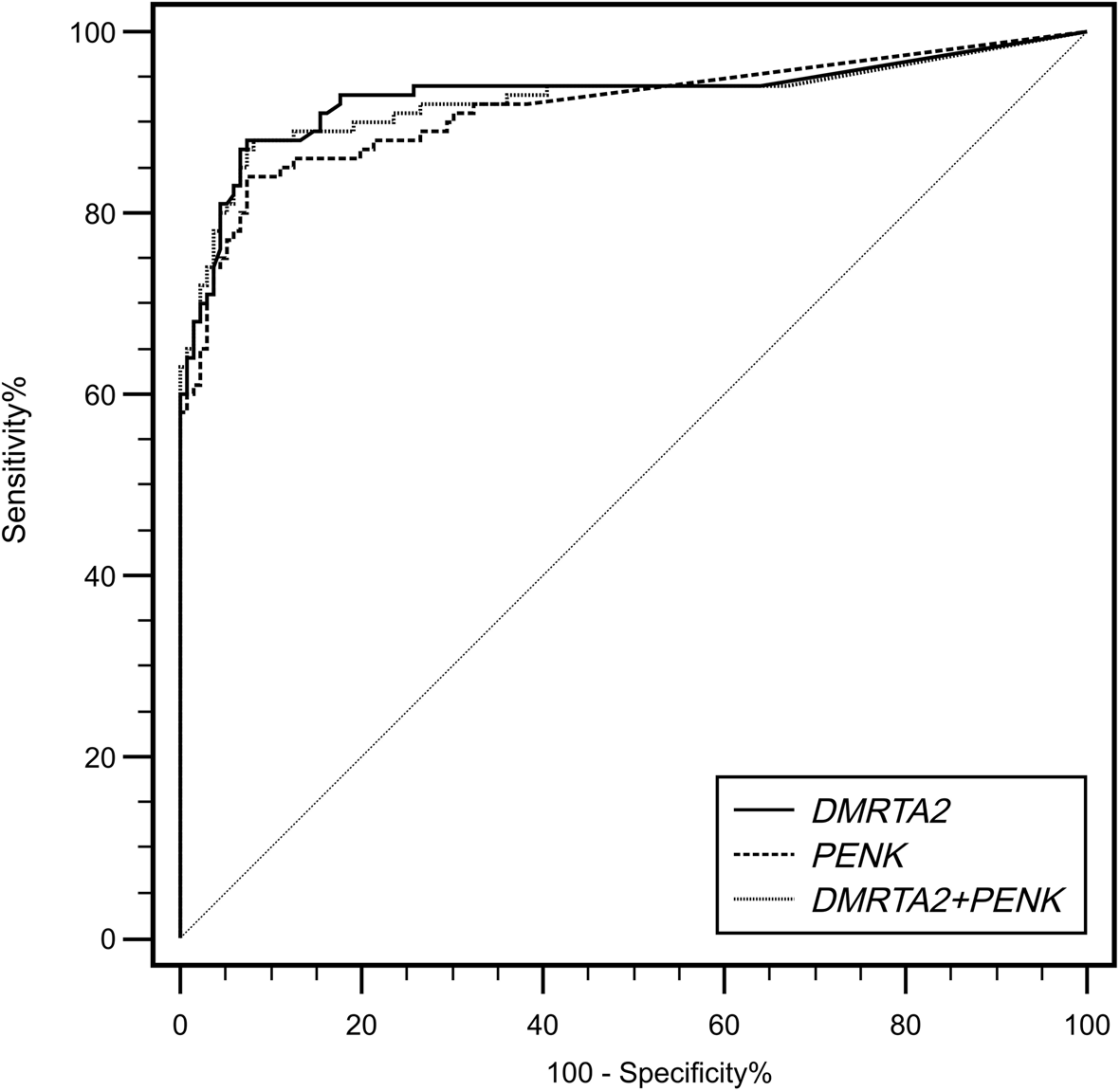
ROC curves and associated AUCs of the diagnostic prediction model using DNA methylation analysis of *DMRTA2* and *PENK*

*DMRTA2* and *PENK* showed very similar assay performance in detecting BC from 237 urine samples. With specificity from a control group consisting of patients with lithangiuria, prostatoplasia, and prostatitis fixed at 95%, the two markers had sensitivities of 84.37 and 78.12%, respectively (Table 1). The AUC value for *DMRTA2* was 0.958, higher than the 0.937 for *PENK* (Table 1, Fig. 2). Furthermore, the sensitivity remained unchanged when the two best markers were combined, and AUC value was 0.955, slightly lower than *DMRTA2* alone (Table 1). Hence, the subsequent assessment of the uDNA test’s performance in independent discovery and validation sets was exclusively performed for *DMRTA2* as the most accurate marker.

**Table 1:** Performance characteristics of m*DMRTA2* and m*PENK* in urine samples

Gene	Specificity (%)	Sensitivity (%)	95% CI	Ct value	AUC (95% CI)
m*DMRTA2*	95	84.37	68.75 to 95.83	≤35.6	0.958 (0.922 to 0.979)
m*PENK*	95	78.12	60.47 to 89.58	≤35.9	0.937 (0.893 to 0.963)
m*DMRTA2 +* m*PENK*	95	84.37	68.75 to 94.79	> 0.564	0.955 (0.905 to 0.976)

### Performance characteristics of *DMRTA2* in a discovery set

**Fig. 3:**
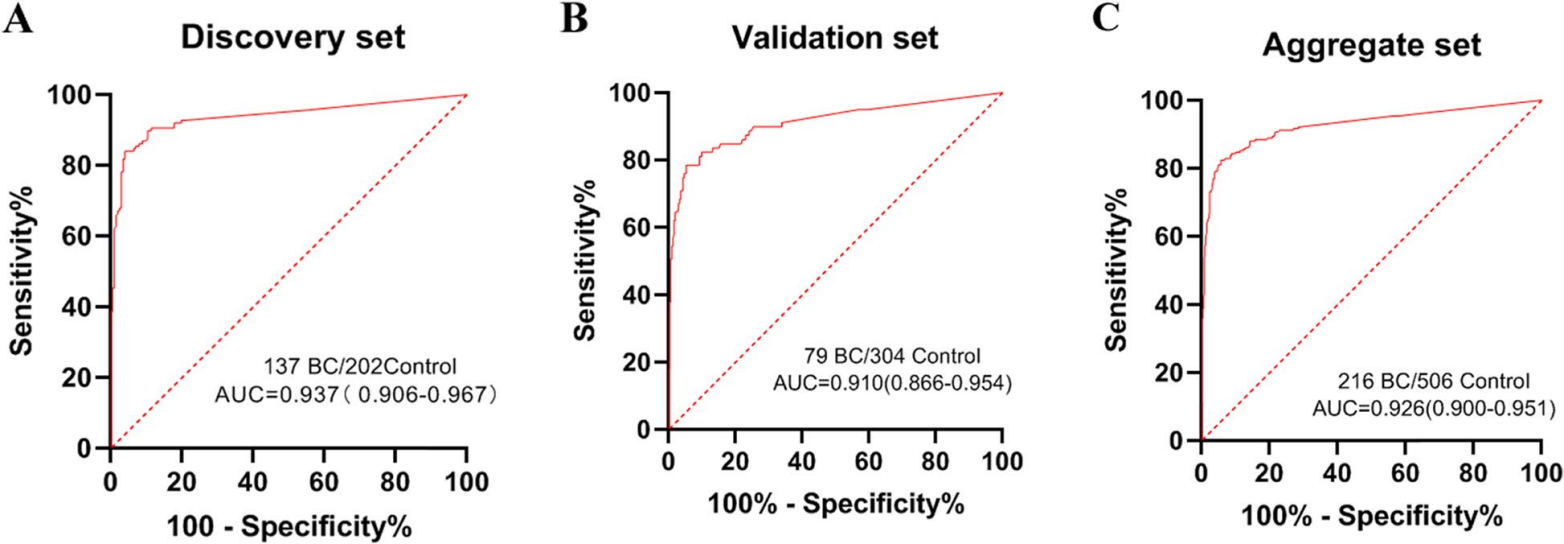
ROC curves and associated AUCs of the diagnostic prediction model using DNA methylation analysis of *DMRTA2* in the discovery (**A**), validation (**B**), and aggregate (**C**) sets. Cutoff CT-value of 37 was used for both discovery and validation set

The best methylation biomarker, m*DMRTA2*, was further tested in a group of 477 urine samples, consisting of 137 BCs, 202 normal controls, 31 renal carcinomas, 36 carcinomas of renal pelvis and ureter, 28 benign tumors of the bladder, 13 prostate cancers, and 30 postoperative patients including 22 recurring cancers (Supplementary Table S7). Overall, at a CT cutoff value of 37 (Supplementary Table S8), the single-target uDNA methylation test had a sensitivity of 85.4% (95% CI: 0.781–0.906), a specificity of 93.1% (95% CI: 0.884–0.960), and an AUC value of 0.937 for BC detection (Table 2, Fig. 3A). Notably, the methylation assay had the highest sensitivities for tumors at stages of T1 (94.1%) and T2 (96.4%) compared with T3 (77.8%) and T4 (71.4%) (Table 3). While methylation was observed in 25/36 urine samples from patients with carcinomas of renal pelvis and ureter, a good sensitivity of 69.4%, it was detected at extremely low rate of 2.3% (1/44) in those with interfering cancers of kidney and prostate (Table 2). The test was also sensitive in detecting recurring cancers in the bladder at 77.3% (17/22) and less sensitive in detecting benign bladder tumors at 10.7% (3/28) (Table 2). Overall, the assay seems to be a feasible methylation-specific testing for early-stage BCs and recurring cancers.

**Table 2:** The sensitivity of *DMRTA2* by different types of disease in urine samples

	Discovery set^**a**^			Validation set^**a**^			Aggregate set		
Type of disease	Samples	***DMRTA2***-positive	Sensitivity (95%CI)	Samples	***DMRTA2***-positive	Sensitivity (95%CI)	Samples	***DMRTA2***-positive	Sensitivity (95%CI)
**Bladder cancer**	137	117	85.4% (78.1–90.6%)	79	62	78.5% (67.5–86.6%)	216	179	82.9% (77.0–87.5%)
**Carcinomas of renal pelvis**	23	18	78.3% (55.8–91.7%)	12	11	91.7% (59.8–99.6%)	35	29	82.9% (65.7–92.8%)
**Carcinomas of ureter**	13	7	53.8% (26.1–79.6%)	6	3	50.0% (13.9–86.1%)	19	10	52.6% (29.5–74.8%)
**Renal carcinomas**	31	0	0	40	2	5.0% (0.8–18.2%)	71	2	2.8% (0.5–10.7%)
**Prostate cancers**	13	1	7.7% (0.4–37.9%)	49	5	10.2% (3.8–23.0%)	62	6	9.7% (4–20.5%)
**Benign tumors of bladder**	28	3	10.7% (2.8–29.4%)	22	7	31.8% (14.7–54.9%)	50	10	20.0% (10.5–34.1%)
**Recurring cancers**	22	17	77.3% (54.2–91.3%)	8	7	87.5% (46.7–99.3%)	30	24	80.0% (60.8–91.6%)
			**Specificity**			**Specificity**			**Specificity**
**Normal**^**b**^	202	14	93.1%^b^ (88.4–96.0%)	304	24	92.1%^b^ (88.3–94.8%)	506	38	92.5%^b^ (89.7–94.6%)

^a^Cutoff CT-value of 37 was used for both discovery and validation set

^b^Normal controls were not healthy donors but subjects with benign diseases of the urinary tract

**Table 3:** The sensitivity of *DMRTA2* to detect BC at different TNM stages in discovery and validation sets

	Discovery set			Validation set		
Stage^**a**^	Bladder cancer (***n*** = 128)	***DMRTA2***- positive	Sensitivity (95%CI)	Bladder cancer (***n*** = 61)	***DMRTA2***- positive	Sensitivity (95%CI)
Total	137	117	85.4% (78.1–90.6%)	79	62	78.5% (67.5–86.6%)
Ta	33	22	66.7% (48.1–81.4%)	21	12	57.1% (34.4–77.4%)
T1	51	48	94.1% (82.8–98.5%)	22	18	81.8% (59.0–94.0%)
T2	28	27	96.4% (79.8–99.8%)	12	11	91.7% (59.8–99.6%)
T3	9	7	77.8% (40.2–96.1%)	2	2	100% (19.8–100%)
T4	7	5	71.4% (30.3–94.9%)	4	4	100% (39.5–100%)

^a^A total of 27 cases of unknown stage (*n* = 9 and 18 in discovery and validation sets, respectively) are not included in the table

### Further validation of *DMRTA2* as the exclusive methylation marker

After evaluation of the methylation levels of *DMRTA2* in our discovery set for BC detection, we further validated its performance in an additional and independent set of 520 urine samples from which 79 were from BC patients, 22 benign growths of the bladder, 304 from control individuals, 107 other types of malignancies, as well as 8 recurring cancers (Supplementary Table S9). At the same CT cut-off value as in the discovery set (Supplementary Table S10), the m*DMRTA2* test was able to identify 62 out of 79 BC cases with a sensitivity of 78.5% (95% CI: 67.5–86.6), which is similar to that of the discovery set (Table 2). For 34 cases whose stage T1 or T2 tumors were confined to bladder walls, the sensitivity was drastically improved to 29 out of 34, at 85.3% (95% CI: 68.1–94.5), significantly higher than that of stage Ta at 57.1% and consistent with the data from the discovery set (Table 3). The sensitivity for recurring BC stood at 87.5% (7/8), which was similar to that for all BC cases, and the specificity of the m*DMRTA2* test was 92.1% (95% CI: 88.3–94.7) for 304 normal controls (Table 2). The AUC value for BC detection was 0.910, representing an excellent diagnostic accuracy with cystoscopy combined with tissue biopsies (Fig. 3B).

When the two independent sample sets (discovery and validation) were combined, the performance indexes for BC detection in a total of 216 urine samples from BC patients and 506 urine samples from controls were 82.9% (95% CI: 77.0–87.5) for sensitivity and 92.5% (95% CI: 89.7–94.6) for specificity, resulting in an AUC value of 0.926 (Table 2, Fig. 3C). In further striated analysis according to TNM stage, the methylation test retains highest sensitivity for stage T1 and T2 tumors, which is similar to the trend observed in both discovery and validation sets (Table 3, Table 4, and Supplementary Table S11). The test performs better in older men (≥60 y) and for high grade neoplasia than low grade ones (*p* < 0.001), but no significant association was observed between level of m*DMRTA2* and gender (*p* > 0.05) (Table 4, Supplementary Table S11). Notably, the uDNA test of *mDMRTA2 was* also sensitive in detecting carcinomas of renal pelvis (29/35, 82.9%), and to a lesser extent, ureter (10/19, 52.6%), but performed poorly in detecting prostate cancer (6/62, 9.7%), clear cell carcinoma of kidney (2/71, 2.8%), and benign tumors of the bladder (10/50, 20.0%) (Table 2).

**Table 4:** The sensitivity and specificity of *DMRTA2* by different clinical characteristics in the aggregate set

Clinical characteristics	***DMRTA2***-positive	Bladder cancer	Sensitivity(95%CI)	***DMRTA2***-negative	Non-cancer disease	Specificity (95%CI)
**Total**	179	216	82.9% (77.0–87.5%)	468	506	92.5% (89.7–94.6%)
**Age**						
< 60	29	44	65.9% (50.0–79.1%)	218	233	93.6% (89.4–96.2%)
60 ~ 69	63	74	85.1% (74.5–92.0%)	156	169	92.3% (86.9–95.7%)
70 ~ 79	62	68	91.2% (81.1–96.3%)	76	85	89.4% (80.4–94.7%)
≥ 80	25	30	83.3% (64.5–93.7%)	18	19	94.7% (71.9–99.7%)
**Sex**						
Male	152	179	84.9% (78.6–89.7%)	281	306	91.8% (88.0–94.5%)
Female	30	37	81.1% (64.3–91.4%)	187	200	93.5% (88.9–96.3%)
**Grade**						
Low	60	84	71.4% (60.4–80.5%)	0	0	NA
High	106	112	94.6% (88.2–97.8%)	0	0	NA
NA	16	20	80% (55.7–93.4%)	0	0	NA
**Stage**						
Ta	34	54	63% (48.7–75.4%)	0	0	NA
T1	66	73	90.4% (80.7–95.7%)	0	0	NA
T2	38	40	95.0% (81.8–99.1%)	0	0	NA
T3	9	11	81.8% (47.8–96.8%)	0	0	NA
T4	9	11	81.8% (47.8–96.8%)	0	0	NA

### DNA methylation status and gene expression of DMRTA2 in BC cell lines and tissues

**Fig. 4:**
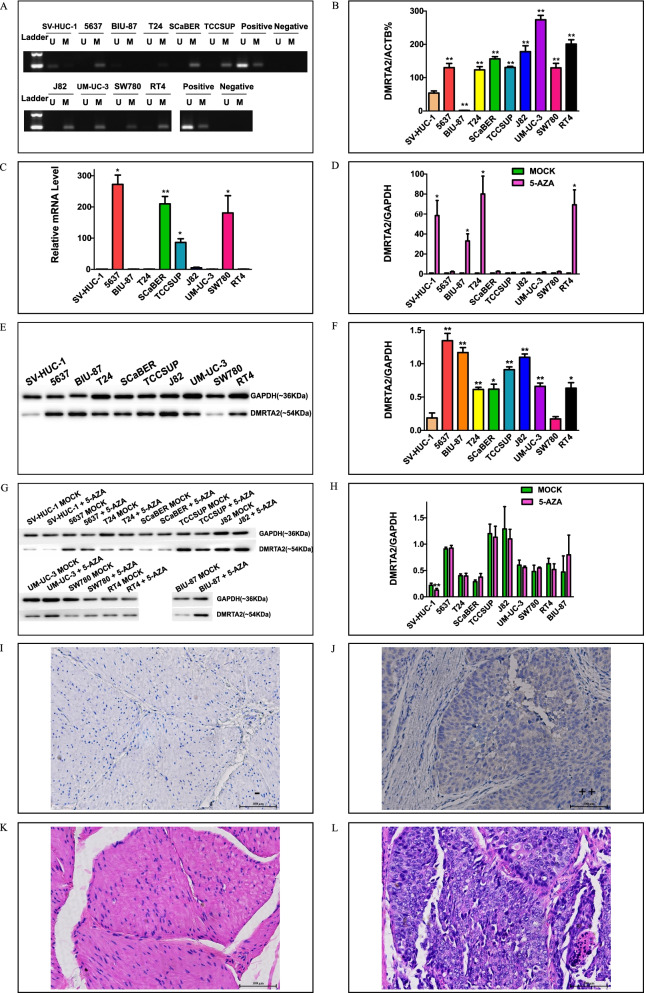
**A** *DMRTA2* methylation in SV-HUC-1 and various BC cell lines detected by MSP. MSP products in lanes U and M indicate the presence of unmethylated and methylated *DMRTA2*, respectively. **B** Quantification of m*DMRTA2* in SV-HUC-1 and various BC cell lines by qMSP with *ACTB* as the reference gene. Data is shown as mean ± s.d. of three independent experiments (*n* = 3). **C** Level of *DMRTA2* mRNA expression in SV-HUC-1 and various BC cell lines by RT-qPCR with *GAPDH* as the reference gene. Data is shown as mean ± s.d. of three independent experiments (*n* = 3). (D) Elevation of *DMRTA2* mRNA expression in SV-HUC-1 and various BC cell lines after 5’-Aza-dC treatment (demethylation) by RT-qPCR with *GAPDH* as the reference gene. Data is shown as mean ± s.d. of three independent experiments (*n* = 3). **E** and **F**, Level of DMRTA2 protein expression detected in SV-HUC-1 and various BC cell lines by western blot analysis with GAPDH as the internal control. Data is shown as mean ± s.d. of three independent experiments (*n* = 3). **G** and **H**, Effect of 5’-Aza-dC treatment (demethylation) on DMRTA2 expression in the same set of cell lines by western blot analysis. Data shown as mean ± s.d. of three independent experiments (*n* = 3). **I** and **J**, Examination of DMRTA2 expression in BC and adjacent normal tissues by IHC. Left panel and right panel show negative and positive staining for two distinct tissue sections. **K** and **L**, Morphological features of tumor and adjacent normal tissue sections revealed by H&E staining. Scale bar is 100 μm. Paired t test was used to analyze statistical significance for experiments with 5’-Aza-dC treatment. Independent t test was used for all other experiments. *p < 0.05 and **p < 0.01

Additionally, we detected level of *mDMRTA2* and its mRNA expression in 9 BC cell lines and 1 normal bladder epithelial cell line by MSP, qMSP and RT-qPCR methods. Compared with SV-HUC-1, the normal bladder epithelial cell line, *DMRTA2* gene was hypermethylated in 8 out of 9 BC cell lines, which is consistent with the results of MSP and qMSP (Fig. 4A–B). Notably, BIU-87 was an outliner, which may be attributable to potential cross-contamination of this line [37]. The mRNA levels of *DMRTA2* were low in some BC cell lines, such as T24, J82, UM-UC-3 and RT4, however, were drastically higher in 5637, SCaBER, TCCSUP and SW780 (Fig. 4C). The *DMRTA2* hypermethylation could be reversed by 5′-aza treatment, drastically reducing methylation level and stimulated its mRNA expression in certain cell lines including SV-HUC-1, T24, and RT4 (Supplementary Table S12 and Fig. 4D). The protein levels in all 10 of the aforementioned cell lines were not significantly increased after demethylation even in the aforementioned 4 cell lines with significant mRNA up-regulation, implying a more complex pattern of gene expression at mRNA and protein synthesis levels for *DMRTA2* (Fig. 4E–H). Further IHC staining of a total of 11 pairs of BC and adjacent normal tissue specimens as well as 8 standalone BC carcinoma sections showed mainly weak staining of DMRTA2 in cancerous cells compared with no recognizable staining in normal tissues (Fig. 4I-L, Supplementary Table S13), which is consistent with the results from western analysis of BC cell lines (Fig. 4F). The unclear mechanism via which the hypermethylated region of *DMRTA2* gene regulates its own expression needs to be further addressed.

## Discussion

In summary, our systematic screening approach of candidate markers did generate two top-performers in *DMRTA2* and *PENK*, who showed similar sensitivity and specificity in detecting BC. Since *PENK* had been analyzed in multigene panels for BC detection in some previous studies [27, 38], and we did not find increased sensitivity when these two genes were combined in a test of a case-control group of 237 urine samples, we evaluated the performance of m*DMRTA2* as the sole biomarker in a large hospital-based cohort. Overall, the single-target uDNA methylation test achieved 82.9% of sensitivity and 92.5% specificity. In particular, m*DMRTA2* is useful in detecting early BC such as T1 and T2 stage tumors with enhanced sensitivity up to 92.0%, a much desirable feature for any in vitro diagnostic test. The single-target test also had an admirable detection rate for recurring BC at 80.0% (Ct cutoff = 37) or 88% (Ct cutoff = 38), comparable to some of the tests currently available on the market. Put together, the simple, non-invasive, and convenient urine-based m*DMRTA2* test offered a much more affordable and attractive option than certain multigene panels, such as EpiChek [23], with comparable performance for aiding early diagnosis and monitoring recurrence of the disease.

Most of the reported methylation markers tested in relatively large cohorts had poor specificity [23, 24, 39–42]. To reduce false positive rate and increase specificity, we included urine samples from 506 patients with lithangiuria, prostatoplasia, and prostatitis, benign diseases routinely seen in outpatient visits, as normal controls. Under such conditions, the m*DMRTA2* test achieved 92.5% specificity at a cut-off Ct value of 37, significantly higher than those reported for FDA-approved urine tests [5, 7]. Moreover, the test’s false positive rate is also lower than the published values for large-scale studies using multigene panels of DNA methylation markers (7.5% versus 15% for a two-gene signature of *GHSR/MAL* [28], 16.9% for a two-marker model of *OTX1/SOX1-OT* [12], 10.3% for a dual-marker panel of *ONECUT2/VIM* and 13.2% for a five-marker panel of *VIM/OSTM1/SLC4A10/AC092805.1/ONECUT2* [29], and 10.0–17.9% for EpiCheck, a 15-marker methylation test, in various clinical trials [23, 43]). The lowest false positive rate is a desirable feature for the current test that may be used in the future for BC screening in a high-risk population with low prevalence to avoid excessive invasive diagnostic tests [1, 2].

The sensitivity of the uDNA methylation test for BC is 82.9%, generally lower than reported values for multigene panels (92% for *GHSR/MAL*, 90.0% for *OTX1/SOX1-OT*, 90.5% for *VIM/OSTM1/SLC4A10/AC092805.1/ONECUT2*, 88.1–91.2% for *ONECUT2/VIM* and 62.5–90.0% for EpiCheck). Similarly, the sensitivity to BC at Ta stage is at 63.0% (34/54), also lower than most of the published results for multigene panels (95% (Ta/T1) for *GHSR/MAL*, 64.5% for *OTX1/SOX1-OT*, 83.3% for *ONECUT2/VIM*, and 51.9% for EpiCheck [23]), but higher than cytology (22.2–41.2%), FISH (44.4–52.9%), and FDA-approved NMP22 test (39–51%) [42]. However, the single-marker test has higher AUC values (0.910–0.937) than most of those for multigene panels (0.86 for *GHSR/MAL*, 0.919 for *OTX1/SOX1-OT*, 0.881–0.889 for *VIM/OSTM1/SLC4A10/AC092805.1/ ONECUT2*, 0.898–0.935 for *ONECUT2/VIM*, and 0.817 for EpiCheck [23]), indicating that the methylation test is adequately sensitive and substantially accurate in risk prediction for BC.

In addition to BC, the uDNA methylation test could also detect other urothelial cancers including those of ureter and renal pelvis with similar sensitivity at 82.9% (29/35) and 52.6% (10/19). The detection rate for all cancer cases of the bladder, ureter, and renal pelvis combined is still fairly remarkable at 80.7% (218/270), implying that the current test is robust in detecting urothelial cancers. Since the prevalence of urothelial cancers is apparently higher than BC alone, the test would have added value to it if tumor types for detection can be expanded to encompass all carcinomas originated in the epithelial cells lining the urinary tract and in close contact with liquid urine. However, the sensitivity for benign tumors of the bladder, kidney cancer, and prostate cancer was greatly reduced to 20% (10/15), 2.8% (2/71), and 9.7% (6/62), respectively. Low sensitivity for the detection of interfering diseases is another desirable feature for the uDNA methylation test to aid diagnosis and prognosis of BC.

In conclusion, the non-invasive, simple, and user-friendly uDNA test of m*DMRTA2* is a feasible diagnostic method with robust sensitivity, superior specificity, and substantial accuracy. First, the highest specificity of the m*DMRTA2* test (92.5%) among its counterparts is a desirable feature to the clinicians as the lowest false positive rate reduces the number of invasive cystoscopic procedures to a minimum. Second, the superior sensitivity of the methytion test to bladder tumors at T1 or T2 stage (92.0%) is another welcoming feature in clinical practice as early detection of BC is always associated with drastically improved 5-year survival [3]. Third, the use of an exclusive methylation marker further makes the testing simple, easy, and more affordable, facilitating its widespread use in clinics. However, in spite of its several advantages, certain notable limitations are still associated with the test. First, the m*DMRTA2* test was not as sensitive in detecting Ta stage tumor as it was in detecting T1–T4 stage tumors. We can incorporate additional markers that have overlapping methylation profiles with *DMRTA2* into the test to increase its sensitivity in detecting localized tumor confined to the epithelial layer of the bladder. Second, even though the m*DMRTA2* test showed a decent detection rate for recurring cancers, the number of cases tested in this study was still very small compared to a couple of previous studies [23]. A large-scale cohort should be established to accurately evaluate the test’s sensitivity for the detection of recurring BC. Third, the proportion of cases of various types of urothelial malignancies does not necessarily reflect their prevalence in the Chinese population because we wanted to evaluate all the cases that had been collected and were available to us. When the number in cancers of bladder, ureter, and renal pelvis was weighted against their actual incidence (carcinomas of ureter and renal pelvis account for 10% of the total), we have estimated that the sensitivity for urothelial carcinomas would improve from 80.7 to 86.9% [44]. Fourth, the effect of prostate cancer to interfere with the detection of BC was not accurately evaluated due to the small number of cases available. Fifth, the current investigation is a feasibility study performed at the bench of a standard laboratory but not in a real-world clinical setting. Hence, the clinical utility of the uDNA test should be further validated in a multi-center clinical trial before m*DMRTA2* can be used as a reliable and marketable biomarker for both diagnosis and recurrence surveillance of BC.

## Supplementary Information

**Additional file 1.**

## Data Availability

All data generated or analyzed during this study are included in this published article and its supplementary information files.
