# Supplementary material for: A novel and sensitive DNA methylation marker for the urine-based liquid biopsies to detect bladder cancer
Source: BMC Cancer. 2022 May 6;22:510. doi: 10.1186/s12885-022-09616-y (PMC9077853; doi:10.1186/s12885-022-09616-y)
Supplement: Supplementary file 1 — Additional file 1. [file 12885_2022_9616_MOESM1_ESM.docx]

**Supplementary Information and Tables**

**Selection of candidate genes and primer pairs**

We initially searched literature to identify 260 tumor-associated candidate genes with informative methylation profiles. A large proportion of the genes were selected from two major publications (Supplementary Reference: Yu et al., Clin Cancer Res, 2007; Zhao et al., Journal of Molecular Biomarkers and Diagnosis, 2015). We then obtained sequences of a total of 291 known primer pairs for these 260 genes from 48 publications (Supplementary Reference) in addition to a set of 133 newly designed primer pairs for 94 additional candidate genes based on methylation data from TCGA and GEO databases. Briefly, to identify 94 additional candidate genes, we conducted paired t test on Illumina 450K methylation data of 21 pairs of BC and adjacent normal tissues in TCGA. To increase specificity, we selected those methylation sites with false discovery rate (FDR) < 0.01 and β-value > 0.2. To increase sensitivity, we obtained methylation data of 20 BC cell lines from GEO (GSE68379) database and selected methylation sites with β-value > 0.5 and methylated in more than 80% of the BC cell lines. Finally, by removing 10 duplicating genes found in the two primer pools, we actually screened 344 candidate genes using 424 primer pairs (Supplementary Table S1) by MSP and qMSP in BC cell lines, tissue samples, and urine specimens to identify top-performing methylation markers for BC.

**Screening for top-performing methylation biomarkers for BC**

The first-round MSP was performed on the 266 genes obtained from literature search in four BC cell lines including 5637, T24, SW780, and TCCSUP and one immortalized epithelial cell line SV-HUC-1. The candidate markers showing hypermethylation in less than half of the BC cell lines were eliminated. After the first-round MSP, the starting panel was whittled down to 176 genes and 204 primer pairs (Supplementary Table S2). All 204 primer pairs were subjected to second-round of MSP in the same set of cell lines to identify those showing hypermethylation in at least 3 out of 4 BC cell lines. A total of 69 genes and 85 primer pairs passed this round of screening and were further evaluated by SYBR green qMSP in the aforementioned cell lines (Supplementary Table S3). The qMSP assays gave rise to 23 genes and 32 primer pairs, which were put into another round of testing by SYBR green qMSP using 10 pairs of tumor and adjacent normal tissues (Supplementary Table S4). Six candidate genes with highest sensitivity and specificity including *CHAD*, *MEIS1*, *DMRTA2*, *PENK*, *CMTM2*, and *DRD4* were remained after this round of screening. Finally, the two top-performing markers, *DMRTA2* and *PENK*, were identified based on sensitivity, specificity, and AUC values after another two rounds of evaluation by TaqMan probe-based qMSP on urine samples from BC patients and normal controls (Figure1, Supplementary Table S5).

**Supplementary Table S1. 424 primer pairs designed and synthesized for distinct methylation sites within 344 candidate genes**

| ID | Ensembl ID | Gene | Region | Biotype | Description | Forward | Reverse | Reference |
| --- | --- | --- | --- | --- | --- | --- | --- | --- |
| US-CG00 | ENSG00000075624 | ACTB | 7:5527147-5563784:-1 | protein_coding | actin beta [Source:HGNC Symbol;Acc:HGNC:132] | tggtgatggaggaggtttagtaagt | aaccaataaaacctactcctcccttaa | [1] |
| US-CG01 | ENSG00000243064 | ABCC13 | 21:14236206-14362754:1 | transcribed_unprocessed_pseudogene | ATP binding cassette subfamily C member 13 (pseudogene) [Source:HGNC Symbol;Acc:HGNC:16022] | gcgggcggtttttattag | caaaaactcgtccgtcca | [2] |
| US-CG02 | ENSG00000091262 | ABCC6 | 16:16148928-16223522:-1 | protein_coding | ATP binding cassette subfamily C member 6 [Source:HGNC Symbol;Acc:HGNC:57] | ggcgttcggggagtt | cgacctcgacccgataat | [2] |
| US-CG03 | ENSG00000006071 | ABCC8 | 11:17392498-17476879:-1 | protein_coding | ATP binding cassette subfamily C member 8 [Source:HGNC Symbol;Acc:HGNC:59] | gacgtgcggtattacgttg | acaaaaacgcgacaaacg | [2] |
| US-CG04 | ENSG00000052850 | ALX4 | 11:44260440-44310166:-1 | protein_coding | ALX homeobox 4 [Source:HGNC Symbol;Acc:HGNC:450] | gagtttgaggttgtcgttcg | aacccgttacgacgctaaac | [2] |
| US-CG05 | ENSG00000134982 | APC | 5:112707498-112846239:1 | protein_coding | APC, WNT signaling pathway regulator [Source:HGNC Symbol;Acc:HGNC:583] | tattgcggagtgcgggtc | tcgacgaactcccgacga | [2][3][4][5][6] |
| US-CG06 | ENSG00000137936 | BCAR3 | 1:93561786-93847150:-1 | protein_coding | BCAR3, NSP family adaptor protein [Source:HGNC Symbol;Acc:HGNC:973] | gcgtttcgggaggaatag | actacgaaacgcaccgact | [2] |
| US-CG07 | ENSG00000171791 | BCL2 | 18:63123346-63320128:-1 | protein_coding | BCL2, apoptosis regulator [Source:HGNC Symbol;Acc:HGNC:990] | gaagtcgtcgtcggtttg | cccgcaccgaacatc | [2][7][8][9] |
| US-CG08 | ENSG00000266524 | GDF10 | 10:47300386-47313547:1 | protein_coding | growth differentiation factor 10 [Source:HGNC Symbol;Acc:HGNC:4215] | gcggtaaagggtcgaagt | aactcgaaccgccgata | [2] |
| US-CG09 | ENSG00000176171 | BNIP3 | 10:131966455-131982013:-1 | protein_coding | BCL2 interacting protein 3 [Source:HGNC Symbol;Acc:HGNC:1084] | tcgttcggtttcgttttg | acgctccgttctacgaca | [2] |
| US-CG10 | ENSG00000012048 | BRCA1 | 17:43044295-43170245:-1 | protein_coding | BRCA1, DNA repair associated [Source:HGNC Symbol;Acc:HGNC:1100] | ggttaatttagagtttcgagagacg | tcaacgaactcacgccgcgcaatcg | [2] |
| US-CG11 | ENSG00000139618 | BRCA2 | 13:32315474-32400266:1 | protein_coding | BRCA2, DNA repair associated [Source:HGNC Symbol;Acc:HGNC:1101] | gcggagattgcgttattg | ccgaacccgtttccttac | [2] |
| US-CG12 | ENSG00000159228 | CBR1 | 21:36069941-36073166:1 | protein_coding | carbonyl reductase 1 [Source:HGNC Symbol;Acc:HGNC:1548] | tcgtatttcggcgaggt | aaaccccgcaacgtattc | [2] |
| US-CG13 | ENSG00000159231 | CBR3 | 21:36134912-36146566:1 | protein_coding | carbonyl reductase 3 [Source:HGNC Symbol;Acc:HGNC:1549] | cgtagattatttcgcggtttag | gaaccgaacttcgaaccac | [2] |
| US-CG14 | ENSG00000133101 | CCNA1 | 13:36431520-36442882:1 | protein_coding | cyclin A1 [Source:HGNC Symbol;Acc:HGNC:1577] | tcgtcgcgttttagtcgt | acccgttctcccaacaac | [2][10] |
| US-CG15 | ENSG00000039068 | CDH1 | 16:68737225-68835548:1 | protein_coding | cadherin 1 [Source:HGNC Symbol;Acc:HGNC:1748] | gtgggcgggtcgttagtttc | ctcacaaatactttacaattccgacg | [2][4][6][11][12][13][14] |
| US-CG16 | ENSG00000140945 | CDH13 | 16:82626803-83800640:1 | protein_coding | cadherin 13 [Source:HGNC Symbol;Acc:HGNC:1753] | tcgcggggttcgtttttcgc | gacgttttcattcatacacgcg | [2][9] |
| US-CG17 | ENSG00000129757 | CDKN1C | 11:2883213-2885773:-1 | protein_coding | cyclin dependent kinase inhibitor 1C [Source:HGNC Symbol;Acc:HGNC:1786] | ggttcggttttcgcgtat | aaaacgaacgtcgcgata | [2] |
| US-CG18 | ENSG00000001626 | CFTR | 7:117465784-117715971:1 | protein_coding | cystic fibrosis transmembrane conductance regulator [Source:HGNC Symbol;Acc:HGNC:1884] | agaggtcgcgattgtcgtt | cgactttctccacccactacg | [2] |
| US-CG19 | ENSG00000203667 | COX20 | 1:244835322-244845057:1 | protein_coding | cytochrome c oxidase assembly factor COX20 [Source:HGNC Symbol;Acc:HGNC:26970] | gttcgtcgttgcgatgtt | ccaaactctttcccaaatca | [2] |
| US-CG20 | ENSG00000196730 | DAPK1 | 9:87497228-87708633:1 | protein_coding | death associated protein kinase 1 [Source:HGNC Symbol;Acc:HGNC:2674] | tcggtaattcgtagcggtag | tactcacccgaacgccta | [2][14][15] |
| US-CG21 | ENSG00000185721 | DRG1 | 22:31399523-31530634:1 | protein_coding | developmentally regulated GTP binding protein 1 [Source:HGNC Symbol;Acc:HGNC:3029] | ggtgcggagtatgagtcg | ccgcgaaccaatacgata | [2] |
| US-CG22 | ENSG00000166923 | GREM1 | 15:32717974-32745107:1 | protein_coding | gremlin 1, DAN family BMP antagonist [Source:HGNC Symbol;Acc:HGNC:2001] | tcggtttcgttgatttcg | aaactaccgcgcgtaaaac | [2][16][17] |
| US-CG23 | ENSG00000136160 | EDNRB | 13:77895481-77975529:-1 | protein_coding | endothelin receptor type B [Source:HGNC Symbol;Acc:HGNC:3180] | tagggcgcgttcgtatag | ccactaacgcgcaaactt | [2] |
| US-CG24 | ENSG00000168040 | FADD | 11:70203163-70207390:1 | protein_coding | Fas associated via death domain [Source:HGNC Symbol;Acc:HGNC:3573] | cgtgacgttcgggttg | cctacgcccgacgtatc | [2] |
| US-CG25 | ENSG00000054983 | GALC | 14:87837820-87993665:-1 | protein_coding | galactosylceramidase [Source:HGNC Symbol;Acc:HGNC:4115] | ggtgacgtcggaagagaag | ccgccacgataaatacga | [2] |
| US-CG26 | ENSG00000084207 | GSTP1 | 11:67583595-67586656:1 | protein_coding | glutathione S-transferase pi 1 [Source:HGNC Symbol;Acc:HGNC:4638] | gcgatttcggggatttta | acgacgacgaaactccaa | [2][4][6][10][18] |
| US-CG27 | ENSG00000125798 | FOXA2 | 20:22581005-22585455:-1 | protein_coding | forkhead box A2 [Source:HGNC Symbol;Acc:HGNC:5022] | cgttcgttgttgtttttgc | aaccgtcgaccgctactaa | [2] |
| US-CG28 | ENSG00000144339 | TMEFF2 | 2:191949043-192195709:-1 | protein_coding | transmembrane protein with EGF like and two follistatin like domains 2 [Source:HGNC Symbol;Acc:HGNC:11867] | aagaggggcgttagttcg | cgctcgcaaacgctaa | [2][19] |
| US-CG29 | ENSG00000164362 | TERT | 5:1253147-1295069:-1 | protein_coding | telomerase reverse transcriptase [Source:HGNC Symbol;Acc:HGNC:11730] | gcgtcgcgaggagag | aattcgcgaacacaaacg | [2][20] |
| US-CG30 | ENSG00000090339 | ICAM1 | 19:10270835-10286615:1 | protein_coding | intercellular adhesion molecule 1 [Source:HGNC Symbol;Acc:HGNC:5344] | tagcgcggtgtagatcgt | cgaactaacaaaatacccgaac | [2] |
| US-CG31 | ENSG00000115232 | ITGA4 | 2:181457202-181536187:1 | protein_coding | integrin subunit alpha 4 [Source:HGNC Symbol;Acc:HGNC:6140] | gacgcgagttttgcgtag | taaaataccgcgcactcg | [2] |
| US-CG32 | ENSG00000053747 | LAMA3 | 18:23689443-23956222:1 | protein_coding | laminin subunit alpha 3 [Source:HGNC Symbol;Acc:HGNC:6483] | ttcgttcgcgaagtttgt | taaacgacgccgaaacc | [2] |
| US-CG33 | ENSG00000189067 | LITAF | 16:11547722-11636381:-1 | protein_coding | lipopolysaccharide induced TNF factor [Source:HGNC Symbol;Acc:HGNC:16841] | cggtcgggtttttacgtt | acctcccgactcgacaa | [2] |
| US-CG34 | ENSG00000198681 | MAGEA1 | X:153179285-153183880:1 | protein_coding | MAGE family member A1 [Source:HGNC Symbol;Acc:HGNC:6796] | gttcggtcgaaggaatttga | ccacaaccctccctcttaaa | [2] |
| US-CG35 | ENSG00000085563 | ABCB1 | 7:87503633-87713323:-1 | protein_coding | ATP binding cassette subfamily B member 1 [Source:HGNC Symbol;Acc:HGNC:40] | ttgggggtttggtagcgc | ctctctaaacccgcgaacgat | [2] |
| US-CG36 | ENSG00000170430 | MGMT | 10:129467184-129768007:1 | protein_coding | O-6-methylguanine-DNA methyltransferase [Source:HGNC Symbol;Acc:HGNC:7059] | agcgtcgttgttttgtgc | cgctttcaaaaccactcg | [2][4][6][13] |
| US-CG37 | ENSG00000034053 | APBA2 | 15:28884483-29118315:1 | protein_coding | amyloid beta precursor protein binding family A member 2 [Source:HGNC Symbol;Acc:HGNC:579] | tgttggtggattttggattt | aacaacaattccatacacctttct | [2] |
| US-CG38 | ENSG00000107282 | APBA1 | 9:69427530-69672306:-1 | protein_coding | amyloid beta precursor protein binding family A member 1 [Source:HGNC Symbol;Acc:HGNC:578] | ttcgaagcgtttgtttgg | cgcctaacctaacgcaca | [2] |
| US-CG39 | ENSG00000205362 | MT1A | 16:56638666-56640087:1 | protein_coding | metallothionein 1A [Source:HGNC Symbol;Acc:HGNC:7393] | taaggttgggttttcggaac | aaatacgaaccacgaaacca | [2] |
| US-CG40 | ENSG00000170873 | MTSS1 | 8:124550790-124728429:-1 | protein_coding | MTSS1, I-BAR domain containing [Source:HGNC Symbol;Acc:HGNC:20443] | tgatatttcggtcgggagt | aaatacaacgcgctcgaa | [2] |
| US-CG41 | ENSG00000129152 | MYOD1 | 11:17719568-17722131:1 | protein_coding | myogenic differentiation 1 [Source:HGNC Symbol;Acc:HGNC:7611] | gacggttttcgacggttt | gcccgaaaccgaatacac | [2] |
| US-CG42 | ENSG00000197822 | OCLN | 5:69492292-69558104:1 | protein_coding | occludin [Source:HGNC Symbol;Acc:HGNC:8104] | tgcgttcgttaggtgagc | cgaatcccaactcgaaaacg | [2] |
| US-CG43 | ENSG00000147889 | CDKN2A | 9:21967753-21995301:-1 | protein_coding | cyclin dependent kinase inhibitor 2A [Source:HGNC Symbol;Acc:HGNC:1787] | gtcgagttcggttttggagg | aaaaccacaacgacgaacg | [2][4][7][9] |
| US-CG44 | ENSG00000147889 | CDKN2A | 9:21967753-21995301:-1 | protein_coding | cyclin dependent kinase inhibitor 2A [Source:HGNC Symbol;Acc:HGNC:1787] | ttattagagggtggggcggatcgc | accccgaaccgcgaccgtaa | [2][4][7][9] |
| US-CG45 | ENSG00000204624 | DISP3 | 1:11479166-11537584:1 | protein_coding | dispatched RND transporter family member 3 [Source:HGNC Symbol;Acc:HGNC:29251] | tttcgcggtcgttttaga | ccgcccacgtacgtataa | [2] |
| US-CG46 | ENSG00000068028 | RASSF1 | 3:50329782-50340980:-1 | protein_coding | Ras association domain family member 1 [Source:HGNC Symbol;Acc:HGNC:9882] | gtgttaacgcgttgcgtatc | aaccccgcgaactaaaaacga | [2] |
| US-CG47 | ENSG00000177519 | RPRM | 2:153477338-153478808:-1 | protein_coding | reprimo, TP53 dependent G2 arrest mediator homolog [Source:HGNC Symbol;Acc:HGNC:24201] | tgagcgtttattcgtagattagc | gaacgaacgccgaaaac | [2][16] |
| US-CG48 | ENSG00000020633 | RUNX3 | 1:24899511-24965121:-1 | protein_coding | runt related transcription factor 3 [Source:HGNC Symbol;Acc:HGNC:10473] | gaggggcggtcgtacgcggg | aaaacgaccgacgcgaacgcctcc | [2][9] |
| US-CG49 | ENSG00000256463 | SALL3 | 18:78980275-79002677:1 | protein_coding | spalt like transcription factor 3 [Source:HGNC Symbol;Acc:HGNC:10527] | gttcgcgtagtcgtcgtc | tactcgaaaaccccgtca | [2] |
| US-CG50 | ENSG00000206075 | SERPINB5 | 18:63476761-63505085:1 | protein_coding | serpin family B member 5 [Source:HGNC Symbol;Acc:HGNC:8949] | tttgcgtgggtcgaga | gcctcgacgacactcc | [2] |
| US-CG51 | ENSG00000112759 | SLC29A1 | 6:44219505-44234151:1 | protein_coding | solute carrier family 29 member 1 (Augustine blood group) [Source:HGNC Symbol;Acc:HGNC:11003] | aaggcgtcggtcgttagt | tataaaccgccgaacgaa | [2] |
| US-CG52 | ENSG00000115415 | STAT1 | 2:190964358-191020960:-1 | protein_coding | signal transducer and activator of transcription 1 [Source:HGNC Symbol;Acc:HGNC:11362] | gtcgttcggtgattggtg | aacgaaaacgcgacgata | [2] |
| US-CG53 | ENSG00000100234 | TIMP3 | 22:32801701-32863043:1 | protein_coding | TIMP metallopeptidase inhibitor 3 [Source:HGNC Symbol;Acc:HGNC:11822] | gcgttttatttcgtttcgtc | cacgataaacccgaacca | [2][4][6][13][14] |
| US-CG54 | ENSG00000103490 | PYCARD | 16:31201485-31203450:-1 | protein_coding | PYD and CARD domain containing [Source:HGNC Symbol;Acc:HGNC:16608] | ttgtagcggggtgagcggc | aacgtccataaacaacaacgcg | [2] |
| US-CG55 | ENSG00000104689 | TNFRSF10A | 8:23190452-23225126:-1 | protein_coding | TNF receptor superfamily member 10a [Source:HGNC Symbol;Acc:HGNC:11904] | gtttttcggtcgggagtt | actcgcccgataataacga | [2] |
| US-CG56 | ENSG00000173535 | TNFRSF10C | 8:23102590-23117437:1 | protein_coding | TNF receptor superfamily member 10c [Source:HGNC Symbol;Acc:HGNC:11906] | agcgtttcggtcgtttg | taccgtatccccgtctcc | [2] |
| US-CG57 | ENSG00000173530 | TNFRSF10D | 8:23135588-23164030:-1 | protein_coding | TNF receptor superfamily member 10d [Source:HGNC Symbol;Acc:HGNC:11907] | gaatcgcgacgatgaaga | cacgcgcacaaactacg | [2] |
| US-CG58 | ENSG00000146072 | TNFRSF21 | 6:47231532-47309905:-1 | protein_coding | TNF receptor superfamily member 21 [Source:HGNC Symbol;Acc:HGNC:13469] | ttgtttagcgtcgtatttatcgt | tcctcaaccgctatcgaa | [2] |
| US-CG59 | ENSG00000186153 | WWOX | 16:78099413-79212667:1 | protein_coding | WW domain containing oxidoreductase [Source:HGNC Symbol;Acc:HGNC:12799] | gcgatattgcggagattg | ccctatcgcccgctac | [2] |
| US-CG61 | ENSG00000112297 | CRYBG1 | 6:106360808-106571978:1 | protein_coding | crystallin beta-gamma domain containing 1 [Source:HGNC Symbol;Acc:HGNC:356] | atagtaattcggtcgggatttgcgc | acgcctaaaacttctaacgccgacg | [10] |
| US-CG62 | ENSG00000110680 | CALCA | 11:14966668-14972354:-1 | protein_coding | calcitonin related polypeptide alpha [Source:HGNC Symbol;Acc:HGNC:1437] | ataaaggtgcggatacgatttc | gaacgctacctatacgataaacg | [10] |
| US-CG63 | ENSG00000118971 | CCND2 | 12:4273772-4305350:1 | protein_coding | cyclin D2 [Source:HGNC Symbol;Acc:HGNC:1583] | tcgaggaatcgaaaaagatatttac | gaaaccgctctttactccga | [9][10][39] |
| US-CG65 | ENSG00000187323 | DCC | 18:52340172-53535903:1 | protein_coding | DCC netrin 1 receptor [Source:HGNC Symbol;Acc:HGNC:2701] | ttgtatggttaagggttgcgaacgcg | tctaaatccctcgcgacaataaaaaacgc | [22] |
| US-CG66 | ENSG00000050165 | DKK3 | 11:11963106-12009769:-1 | protein_coding | dickkopf WNT signaling pathway inhibitor 3 [Source:HGNC Symbol;Acc:HGNC:2893] | gttcggttcggtttttagttattc | cgctacgcaatttatactacg | [22] |
| US-CG67 | ENSG00000091831 | ESR1 | 6:151656691-152129619:1 | protein_coding | estrogen receptor 1 [Source:HGNC Symbol;Acc:HGNC:3467] | tcgagttcgttgatgttattgtattcgtc | accgacctcgcgcaccgtataaccg | [22] |
| US-CG68 | ENSG00000189283 | FHIT | 3:59749310-61251459:-1 | protein_coding | fragile histidine triad [Source:HGNC Symbol;Acc:HGNC:3701] | ttggggcgcgggtttgggtttttacgc | cgtaaacgacgccgaccccacta | [14][23] |
| US-CG69 | ENSG00000177374 | HIC1 | 17:2054154-2063241:1 | protein_coding | HIC ZBTB transcriptional repressor 1 [Source:HGNC Symbol;Acc:HGNC:4909] | gtcgcggttcggtggttttgagttc | ccgcctcgaatacatcttctaacgcg | [14][24] |
| US-CG70 | ENSG00000078399 | HOXA9 | 7:27162435-27175180:-1 | protein_coding | homeobox A9 [Source:HGNC Symbol;Acc:HGNC:5109] | agttgggagttacggcgaggtaaac | aaaatcgaaaacgaccaacaaattcg | [25] |
| US-CG71 | ENSG00000125144 | MT1G | 16:56666731-56668065:-1 | protein_coding | metallothionein 1G [Source:HGNC Symbol;Acc:HGNC:7399] | tttgtattcgatttattgagttcgagtc | gaaaaccgctacgaaaaaaccgt | [26] |
| US-CG72 | ENSG00000138650 | PCDH10 | 4:133149315-133208606:1 | protein_coding | protocadherin 10 [Source:HGNC Symbol;Acc:HGNC:13404] | tcgaggtgcgcgattatgggtagtc | acgaaatttccccgccgccaaaacg | [16][22][27] |
| US-CG73 | ENSG00000181195 | PENK | 8:56436674-56446734:-1 | protein_coding | proenkephalin [Source:HGNC Symbol;Acc:HGNC:8831] | tggtcgtgtggggagttatcgagcg | cgacgccttcgcgaaaaaaatcga | [16][22][27] |
| US-CG74 | ENSG00000104332 | SFRP1 | 8:41261958-41309497:-1 | protein_coding | secreted frizzled related protein 1 [Source:HGNC Symbol;Acc:HGNC:10776] | tgtagttttcggagttagtgtcgcgc | cctacgatcgaaaacgacgcgaacg | [16][22][27] |
| US-CG75 | ENSG00000145423 | SFRP2 | 4:153780592-153789120:-1 | protein_coding | secreted frizzled related protein 2 [Source:HGNC Symbol;Acc:HGNC:10777] | gggtcggagtttttcggagttgcgc | ccgctctcttcgctaaatacgactcg | [22][28] |
| US-CG77 | ENSG00000118526 | TCF21 | 6:133889138-133895553:1 | protein_coding | transcription factor 21 [Source:HGNC Symbol;Acc:HGNC:11632] | ggtttttttagaagggtcgcggc | ctcaacactcgcatacgaacccg | [29][30] |
| US-CG78 | ENSG00000144339 | TMEFF2 | 2:191949043-192195709:-1 | protein_coding | transmembrane protein with EGF like and two follistatin like domains 2 [Source:HGNC Symbol;Acc:HGNC:11867] | agttcggatttcgtttcggtagggggc | cgcgaaaaccacaaataaactcgctcg | [19] |
| US-CG79 | ENSG00000026025 | VIM | 10:17228259-17237593:1 | protein_coding | vimentin [Source:HGNC Symbol;Acc:HGNC:12692] | tcgtttcgaggttttcgcgttagagac | cgactaaaactcgaccgactcgcga | [19][25] |
| US-CG80 | ENSG00000156076 | WIF1 | 12:65050626-65121566:-1 | protein_coding | WNT inhibitory factor 1 [Source:HGNC Symbol;Acc:HGNC:18081] | tgtcgtatttacgtaatttggc | gcccaaccgtctaaacga | [22][31] |
| US-CG123 | ENSG00000052850 | ALX4 | 11:44260440-44310166:-1 | protein_coding | ALX homeobox 4 [Source:HGNC Symbol;Acc:HGNC:450] | tgcgttttttattgcgagtcgtcggtc | gacgacgactaaaactacgaaatcgacg | [2][48] |
| US-CG124 | ENSG00000134982 | APC | 5:112707498-112846239:1 | protein_coding | APC, WNT signaling pathway regulator [Source:HGNC Symbol;Acc:HGNC:583] | cgcgagggtatattttcgaggggtacg | ccgacgaaaataaaaaacgccctaatccg | [3][4][5][6] |
| US-CG125 | ENSG00000266524 | GDF10 | 10:47300386-47313547:1 | protein_coding | growth differentiation factor 10 [Source:HGNC Symbol;Acc:HGNC:4215] | cgcgagttcgttaaggtagcgcgtc | gcgatccgtaaataccgataacccga | [48] |
| US-CG126 | ENSG00000012048 | BRCA1 | 17:43044295-43170245:-1 | protein_coding | BRCA1, DNA repair associated [Source:HGNC Symbol;Acc:HGNC:1100] | tacggttagaatggacgttaaggtc | aaaaacgactttttccgtatctccg | [2] |
| US-CG127 | ENSG00000139618 | BRCA2 | 13:32315474-32400266:1 | protein_coding | BRCA2, DNA repair associated [Source:HGNC Symbol;Acc:HGNC:1101] | tcgtagttttagtttagcgtggc | aacgaaactcgaatttacttaacga | [48] |
| US-CG128 | ENSG00000133101 | CCNA1 | 13:36431520-36442882:1 | protein_coding | cyclin A1 [Source:HGNC Symbol;Acc:HGNC:1577] | ttcgtggggatttcgagtac | aaatacaatcgcgacaatattacg | [2][10] |
| US-CG129 | ENSG00000196730 | DAPK1 | 9:87497228-87708633:1 | protein_coding | death associated protein kinase 1 [Source:HGNC Symbol;Acc:HGNC:2674] | attggtcgtttgtcggtcgtttttc | aatccccgacgttaactcgatccg | [14][15] |
| US-CG130 | ENSG00000136160 | EDNRB | 13:77895481-77975529:-1 | protein_coding | endothelin receptor type B [Source:HGNC Symbol;Acc:HGNC:3180] | cgtttttgtaaatcgtagagataatgac | tacgtaacgaaaatcctaccgt | [48] |
| US-CG131 | ENSG00000084207 | GSTP1 | 11:67583595-67586656:1 | protein_coding | glutathione S-transferase pi 1 [Source:HGNC Symbol;Acc:HGNC:4638] | ttcggggtgtagcggtcgtc | gccccaatactaaatcacgacg | [4][6][10][18] |
| US-CG132 | ENSG00000053747 | LAMA3 | 18:23689443-23956222:1 | protein_coding | laminin subunit alpha 3 [Source:HGNC Symbol;Acc:HGNC:6483] | gatttcgtggaagtatcgtatcgg | gaaacgcctacatctcttaaaacgc | [48] |
| US-CG133 | ENSG00000143995 | MEIS1 | 2:66433452-66573869:1 | protein_coding | Meis homeobox 1 [Source:HGNC Symbol;Acc:HGNC:7000] | ttttggtattgggaggtttcgtc | gctaccgaaatactaaactccgac | [48] |
| US-CG134 | ENSG00000170430 | MGMT | 10:129467184-129768007:1 | protein_coding | O-6-methylguanine-DNA methyltransferase [Source:HGNC Symbol;Acc:HGNC:7059] | atcgggatttttattaagcgggcgtc | gcgaacgtacaaacgacctaccacg | [4][6][13] |
| US-CG135 | ENSG00000115507 | OTX1 | 2:63050057-63057836:1 | protein_coding | orthodenticle homeobox 1 [Source:HGNC Symbol;Acc:HGNC:8521] | tcgttgcgtggtgtttagac | gaaaattctactccgacccg | [48] |
| US-CG136 | ENSG00000138650 | PCDH10 | 4:133149315-133208606:1 | protein_coding | protocadherin 10 [Source:HGNC Symbol;Acc:HGNC:13404] | ttatagttggttaagcgcgtttttc | atacaccgctacgaatatctacgaa | [48] |
| US-CG137 | ENSG00000068028 | RASSF1 | 3:50329782-50340980:-1 | protein_coding | Ras association domain family member 1 [Source:HGNC Symbol;Acc:HGNC:9882] | cgtattcggttggagcgtgttaacgc | caaaaatcgcaccacgtatacgtaacga | [48] |
| US-CG138 | ENSG00000106483 | SFRP4 | 7:37905932-38025695:-1 | protein_coding | secreted frizzled related protein 4 [Source:HGNC Symbol;Acc:HGNC:10778] | gggtgatgttatcgtttttgtatcgac | cctcccctaacgtaaactcgaaacg | [48] |
| US-CG139 | ENSG00000114026 | OGG1 | 3:9749944-9788219:1 | protein_coding | 8-oxoguanine DNA glycosylase [Source:HGNC Symbol;Acc:HGNC:8125] | gtcggagttgtgttcgtttc | ctatccgtcttcaaaaccacg | [48] |
| US-CG140 | ENSG00000085563 | ABCB1 | 7:87503633-87713323:-1 | protein_coding | ATP binding cassette subfamily B member 1 [Source:HGNC Symbol;Acc:HGNC:40] | cggggatttgttttttgagttcgc | ctaaactttacccgccgccaatacg | [48] |
| US-CG141 | ENSG00000120437 | ACAT2 | 6:159760328-159779055:1 | protein_coding | acetyl-CoA acetyltransferase 2 [Source:HGNC Symbol;Acc:HGNC:94] | cgcggttttgttcgtaagtttagatttc | gcgcatcccactatacgcaaacg | [48] |
| US-CG142 | ENSG00000148848 | ADAM12 | 10:126012381-126388455:-1 | protein_coding | ADAM metallopeptidase domain 12 [Source:HGNC Symbol;Acc:HGNC:190] | cgtcgtttttcggggtagttttggatttc | ctcgccctaaccgatactctactcgcg | [48] |
| US-CG143 | ENSG00000154734 | ADAMTS1 | 21:26835747-26845409:-1 | protein_coding | ADAM metallopeptidase with thrombospondin type 1 motif 1 [Source:HGNC Symbol;Acc:HGNC:217] | ttagggtgcgttatcggac | taaaacaacgcgaaaattaatacctaacg | [48] |
| US-CG144 | ENSG00000166106 | ADAMTS15 | 11:130448974-130476641:1 | protein_coding | ADAM metallopeptidase with thrombospondin type 1 motif 15 [Source:HGNC Symbol;Acc:HGNC:16305] | gttgtttcgtagcgttggc | ccgttaatatccgaatccaatcg | [48] |
| US-CG145 | ENSG00000134917 | ADAMTS8 | 11:130404925-130428993:-1 | protein_coding | ADAM metallopeptidase with thrombospondin type 1 motif 8 [Source:HGNC Symbol;Acc:HGNC:224] | tacggtgtcggagaagaagtagtcgc | acgcccgaattcaaaatcgaacg | [48] |
| US-CG146 | ENSG00000144843 | ADPRH | 3:119579268-119589945:1 | protein_coding | ADP-ribosylarginine hydrolase [Source:HGNC Symbol;Acc:HGNC:269] | ttaagcgagagtggacgggattttc | tcttcaaaacatttaaaacgaacgacg | [48] |
| US-CG147 | ENSG00000184254 | ALDH1A3 | 15:100877714-100916626:1 | protein_coding | aldehyde dehydrogenase 1 family member A3 [Source:HGNC Symbol;Acc:HGNC:409] | gtttcgaagtcgggaaatcgaggtc | aaacgaacaacgcaaaaacacgtcg | [48] |
| US-CG148 | ENSG00000162551 | ALPL | 1:21509372-21578412:1 | protein_coding | alkaline phosphatase, liver/bone/kidney [Source:HGNC Symbol;Acc:HGNC:438] | tattaagagacgtagaaggaagatgtcgac | taccgaaaacgaaactaaaaacgaa | [48] |
| US-CG149 | ENSG00000180071 | ANKRD18A | 9:38571358-38620660:-1 | protein_coding | ankyrin repeat domain 18A [Source:HGNC Symbol;Acc:HGNC:23643] | tttttttcgaagagagatcggggtc | gacgactacaccgaaacaaaaaccg | [48] |
| US-CG150 | ENSG00000103490 | PYCARD | 16:31201485-31203450:-1 | protein_coding | PYD and CARD domain containing [Source:HGNC Symbol;Acc:HGNC:16608] | aggcggggagtttaggtttcgtttc | tcctcgacgatcaaattctccaacg | [48] |
| US-CG151 | ENSG00000183734 | ASCL2 | 11:2268495-2270952:-1 | protein_coding | achaete-scute family bHLH transcription factor 2 [Source:HGNC Symbol;Acc:HGNC:739] | gtcgtgttttagatcgagtcgc | ctaacgaactccccgacgat | [48] |
| US-CG152 | ENSG00000149311 | ATM | 11:108222484-108369102:1 | protein_coding | ATM serine/threonine kinase [Source:HGNC Symbol;Acc:HGNC:795] | ggtgagagtttcggagttcgagtc | cgcaaaacacgatatacccatacg | [48] |
| US-CG153 | ENSG00000103126 | AXIN1 | 16:287440-352673:-1 | protein_coding | axin 1 [Source:HGNC Symbol;Acc:HGNC:903] | aggtcgtggcggtttttac | gtaaaacgcaacccttttctaccg | [48] |
| US-CG154 | ENSG00000143032 | BARHL2 | 1:90711539-90717237:-1 | protein_coding | BarH like homeobox 2 [Source:HGNC Symbol;Acc:HGNC:954] | cgttagtagtcggattataagcgaac | aaaaattacgaaacaaacacgaccg | [48] |
| US-CG155 | ENSG00000004838 | ZMYND10 | 3:50341110-50346852:-1 | protein_coding | zinc finger MYND-type containing 10 [Source:HGNC Symbol;Acc:HGNC:19412] | agattcgttcggatttaggc | caattacgaaacgattaaaaaacgt | [48] |
| US-CG156 | ENSG00000168487 | BMP1 | 8:22164736-22212326:1 | protein_coding | bone morphogenetic protein 1 [Source:HGNC Symbol;Acc:HGNC:1067] | tcgtttcgggtcggttttttcgttc | acgaactttcgacgccctccaacg | [48] |
| US-CG157 | ENSG00000125845 | BMP2 | 20:6767664-6780280:1 | protein_coding | bone morphogenetic protein 2 [Source:HGNC Symbol;Acc:HGNC:1069] | agtatagttattcgtttcggcggttcgggattc | gactctaacgccccgtcgtcctcga | [48] |
| US-CG158 | ENSG00000152785 | BMP3 | 4:81030965-81057531:1 | protein_coding | bone morphogenetic protein 3 [Source:HGNC Symbol;Acc:HGNC:1070] | tttagcgttggagtggagacggcgttc | cgcgaccgaatacaacgaaataacga | [48] |
| US-CG159 | ENSG00000169594 | BNC1 | 15:83255903-83284714:-1 | protein_coding | basonuclin 1 [Source:HGNC Symbol;Acc:HGNC:1081] | taagacgttcgtttcgtattttttc | gaaacgctctaaaattctctaacg | [16] |
| US-CG160 | ENSG00000152430 | BOLL | 2:197726879-197786762:-1 | protein_coding | boule homolog, RNA binding protein [Source:HGNC Symbol;Acc:HGNC:14273] | ttagcgaagttcgagtaggtaaatc | tcgccttaaacgactaaatcg | [48] |
| US-CG161 | ENSG00000174744 | BRMS1 | 11:66337333-66345125:-1 | protein_coding | BRMS1, transcriptional repressor and anoikis regulator [Source:HGNC Symbol;Acc:HGNC:17262] | gttcggtaggttgcggtaaattc | gcgaccaacaacaactaataaaaaacg | [48] |
| US-CG162 | ENSG00000112276 | BVES | 6:105096822-105137174:-1 | protein_coding | blood vessel epicardial substance [Source:HGNC Symbol;Acc:HGNC:1152] | cgttgcggttatagagatttttaac | gacgaaaaaaacaaaaacgaa | [48] |
| US-CG163 | ENSG00000064012 | CASP8 | 2:201233443-201287711:1 | protein_coding | caspase 8 [Source:HGNC Symbol;Acc:HGNC:1509] | gtcgaggttggcggattattc | accgaatacctacgattacaaacgc | [34] |
| US-CG164 | ENSG00000118971 | CCND2 | 12:4273772-4305350:1 | protein_coding | cyclin D2 [Source:HGNC Symbol;Acc:HGNC:1583] | tcgaggaatcgaaaaagatatttac | gaaaccgctctttactccga | [9][10][21] |
| US-CG165 | ENSG00000106991 | ENG | 9:127815012-127854756:-1 | protein_coding | endoglin [Source:HGNC Symbol;Acc:HGNC:3349] | ttcggtcgtttgtttttttc | gcgatccatactatccacgta | [48] |
| US-CG166 | ENSG00000174059 | CD34 | 1:207880972-207911402:-1 | protein_coding | CD34 molecule [Source:HGNC Symbol;Acc:HGNC:1662] | tcgagtagtgttttttattcggtgc | gcgacatcctaaaccctacg | [48] |
| US-CG167 | ENSG00000026508 | CD44 | 11:35138870-35232402:1 | protein_coding | CD44 molecule (Indian blood group) [Source:HGNC Symbol;Acc:HGNC:1681] | ttattttcgtttcgtttttcgtc | acgaacactcaccgatctacgcc | [6] |
| US-CG168 | ENSG00000172116 | CD8B | 2:86815339-86861924:-1 | protein_coding | CD8b molecule [Source:HGNC Symbol;Acc:HGNC:1707] | ggtcgcgtagttgataggtaaggc | cgatcccccgaaataatacgat | [48] |
| US-CG169 | ENSG00000184661 | CDCA2 | 8:25458997-25507920:1 | protein_coding | cell division cycle associated 2 [Source:HGNC Symbol;Acc:HGNC:14623] | gatttcgggtttcgggttcgaagagc | cgaaaaaacaaccccgtaacaacgcga | [48] |
| US-CG170 | ENSG00000140945 | CDH13 | 16:82626803-83800640:1 | protein_coding | cadherin 13 [Source:HGNC Symbol;Acc:HGNC:1753] | cgtttttagtcggataaaatgtagtc | tccgaatacaaaaacgaaacg | [2][9] |
| US-CG171 | ENSG00000165556 | CDX2 | 13:27962137-27971139:-1 | protein_coding | caudal type homeobox 2 [Source:HGNC Symbol;Acc:HGNC:1806] | gttgtcgtcgagtagttgtttttc | cgaatttatctccgaaccgta | [48] |
| US-CG172 | ENSG00000116254 | CHD5 | 1:6101793-6180123:-1 | protein_coding | chromodomain helicase DNA binding protein 5 [Source:HGNC Symbol;Acc:HGNC:16816] | agttttaattcgtgcgttgtcggatc | gaccgactacgaaactcgaactacg | [35] |
| US-CG173 | ENSG00000183765 | CHEK2 | 22:28687743-28742422:-1 | protein_coding | checkpoint kinase 2 [Source:HGNC Symbol;Acc:HGNC:16627] | ggtagaagaatttgttttttcgttc | gctaaactacgatactacgtcgc | [48] |
| US-CG174 | ENSG00000072609 | CHFR | 12:132822187-132956304:-1 | protein_coding | checkpoint with forkhead and ring finger domains [Source:HGNC Symbol;Acc:HGNC:20455] | tttcgcgggttattttcggtttc | ccgccgctatcaaaaaacattacgac | [48] |
| US-CG175 | ENSG00000136305 | CIDEB | 14:24305096-24311430:-1 | protein_coding | cell death-inducing DFFA-like effector b [Source:HGNC Symbol;Acc:HGNC:1977] | cgagtattagttcgaaaggaagtac | cgccgcctactactaacgat | [48] |
| US-CG176 | ENSG00000149972 | CNTN5 | 11:99020953-100358885:1 | protein_coding | contactin 5 [Source:HGNC Symbol;Acc:HGNC:2175] | taatataattaattacggcgtgggggagc | cgcaacaaacgaaacccaaataacg | [48] |
| US-CG177 | ENSG00000174469 | CNTNAP2 | 7:146116002-148420998:1 | protein_coding | contactin associated protein like 2 [Source:HGNC Symbol;Acc:HGNC:13830] | ggtaatattcgtacggagggagaacg | gcgaaaaaaccaaacccctcccg | [48] |
| US-CG178 | ENSG00000175315 | CST6 | 11:66011841-66013505:1 | protein_coding | cystatin E/M [Source:HGNC Symbol;Acc:HGNC:2478] | tcgagtttcgttttagttttaggtc | ataaccgtcaataccgtcgaa | [16] |
| US-CG179 | ENSG00000101160 | CTSZ | 20:58995185-59007247:-1 | protein_coding | cathepsin Z [Source:HGNC Symbol;Acc:HGNC:2547] | cggagcgggatttaggagtc | gataacaaatctatccccgacgaa | [48] |
| US-CG180 | ENSG00000107562 | CXCL12 | 10:44370165-44386493:-1 | protein_coding | C-X-C motif chemokine ligand 12 [Source:HGNC Symbol;Acc:HGNC:10672] | gagatttcggttacggttagtattc | gaaaaataaaaatacgacgat | [36] |
| US-CG181 | ENSG00000138061 | CYP1B1 | 2:38066973-38109902:-1 | protein_coding | cytochrome P450 family 1 subfamily B member 1 [Source:HGNC Symbol;Acc:HGNC:2597] | gcgtttaggaagattatagagtc | ttaacgtaaaataccgaccg | [48] |
| US-CG182 | ENSG00000136848 | DAB2IP | 9:121567057-121785530:1 | protein_coding | DAB2 interacting protein [Source:HGNC Symbol;Acc:HGNC:17294] | tagtagagtttcggttgtcgttc | gactcgcaaatcgaactaa | [37] |
| US-CG183 | ENSG00000082397 | EPB41L3 | 18:5392381-5630700:-1 | protein_coding | erythrocyte membrane protein band 4.1 like 3 [Source:HGNC Symbol;Acc:HGNC:3380] | cgagtgttttttggttgttgtc | aaaaaccataaacttaacgaacgta | [48] |
| US-CG184 | ENSG00000112977 | DAP | 5:10679230-10761272:-1 | protein_coding | death associated protein [Source:HGNC Symbol;Acc:HGNC:2672] | tagttaggagttcggttgagcgcgttc | tccccgactaactacgaaaactaaaattacg | [48] |
| US-CG185 | ENSG00000035664 | DAPK2 | 15:63907036-64072033:-1 | protein_coding | death associated protein kinase 2 [Source:HGNC Symbol;Acc:HGNC:2675] | ggttcgtcgttttaatgtttgac | accgaacgtactctacccgtt | [48] |
| US-CG186 | ENSG00000167657 | DAPK3 | 19:3958453-3971123:-1 | protein_coding | death associated protein kinase 3 [Source:HGNC Symbol;Acc:HGNC:2676] | cggttataggtgagttatttattcgt | aatttcattaactacaatcgacgct | [48] |
| US-CG187 | ENSG00000107984 | DKK1 | 10:52314296-52318042:1 | protein_coding | dickkopf WNT signaling pathway inhibitor 1 [Source:HGNC Symbol;Acc:HGNC:2891] | cgaattttttttatagtcgtattc | gaaacaaatttacacgcctacg | [14][38] |
| US-CG188 | ENSG00000164741 | DLC1 | 8:13083361-13604610:-1 | protein_coding | DLC1 Rho GTPase activating protein [Source:HGNC Symbol;Acc:HGNC:2897] | tgtataggaagcgagacgttttc | accgacattacgactaacactacg | [39] |
| US-CG189 | ENSG00000008226 | DLEC1 | 3:38039205-38124025:1 | protein_coding | DLEC1, cilia and flagella associated protein [Source:HGNC Symbol;Acc:HGNC:2899] | tatagcgatgacgggattc | cgactaataacgaaattaacg | [48] |
| US-CG190 | ENSG00000142700 | DMRTA2 | 1:50417550-50423500:-1 | protein_coding | DMRT like family A2 [Source:HGNC Symbol;Acc:HGNC:13908] | gcgttgcgtaggtagtaggc | gaacctcgaaaaaataataccgtt | [48] |
| US-CG191 | ENSG00000137857 | DUOX1 | 15:45129933-45165576:1 | protein_coding | dual oxidase 1 [Source:HGNC Symbol;Acc:HGNC:3062] | attttatttcgttttattgcgtttc | gtccgatacctctacaactctacg | [48] |
| US-CG192 | ENSG00000140279 | DUOX2 | 15:45092650-45114344:-1 | protein_coding | dual oxidase 2 [Source:HGNC Symbol;Acc:HGNC:13273] | cgtggattttttaattcgc | ctaacgaaacactacgcctcg | [48] |
| US-CG193 | ENSG00000115380 | EFEMP1 | 2:55865967-55924139:-1 | protein_coding | EGF containing fibulin extracellular matrix protein 1 [Source:HGNC Symbol;Acc:HGNC:3218] | cggatttcgttagattagatttacg | cgttaaaaactcctacctatacg | [48] |
| US-CG194 | ENSG00000172889 | EGFL7 | 9:136658856-136672678:1 | protein_coding | EGF like domain multiple 7 [Source:HGNC Symbol;Acc:HGNC:20594] | tgttggtttatttggcgtcgtc | cccgtaatactctccgaaccgat | [48] |
| US-CG195 | ENSG00000142227 | EMP3 | 19:48321509-48330553:1 | protein_coding | epithelial membrane protein 3 [Source:HGNC Symbol;Acc:HGNC:3335] | cgaggttttataagacggagttc | gaacgtaataaacgaccaaacg | [48] |
| US-CG196 | ENSG00000119888 | EPCAM | 2:47345158-47387601:1 | protein_coding | epithelial cell adhesion molecule [Source:HGNC Symbol;Acc:HGNC:11529] | agatttttaacgtcgttatggagac | gttaataaatcaccgcccga | [48] |
| US-CG197 | ENSG00000140009 | ESR2 | 14:64084232-64338112:-1 | protein_coding | estrogen receptor 2 [Source:HGNC Symbol;Acc:HGNC:3468] | agatcgtcgggttttttggttc | gcttacaactacctcctaacgaa | [48] |
| US-CG198 | ENSG00000106038 | EVX1 | 7:27242700-27250493:1 | protein_coding | even-skipped homeobox 1 [Source:HGNC Symbol;Acc:HGNC:3506] | tttttattacggtttggaagatgac | gaaaaccaaaaactaacttatcg | [48] |
| US-CG199 | ENSG00000174279 | EVX2 | 2:176077472-176083913:-1 | protein_coding | even-skipped homeobox 2 [Source:HGNC Symbol;Acc:HGNC:3507] | gaaaattcgtagtattcggttcgt | acgatactttcgctacccgta | [48] |
| US-CG200 | ENSG00000064655 | EYA2 | 20:46894624-47188844:1 | protein_coding | EYA transcriptional coactivator and phosphatase 2 [Source:HGNC Symbol;Acc:HGNC:3520] | tttcggcgtaggtagtagtcgc | gacctaaaataaacgccgctaacga | [48] |
| US-CG202 | ENSG00000158161 | EYA3 | 1:27970344-28088696:-1 | protein_coding | EYA transcriptional coactivator and phosphatase 3 [Source:HGNC Symbol;Acc:HGNC:3521] | cgttaacgtcgcggtttttttcgttc | ttcacgctatactcgcccgactacg | [48] |
| US-CG203 | ENSG00000112319 | EYA4 | 6:133240598-133532120:1 | protein_coding | EYA transcriptional coactivator and phosphatase 4 [Source:HGNC Symbol;Acc:HGNC:3522] | cgccaccgactactacgaactcgta | ataaaaacggagtgggtttttcgcg | [48] |
| US-CG204 | ENSG00000121769 | FABP3 | 1:31365625-31376850:-1 | protein_coding | fatty acid binding protein 3 [Source:HGNC Symbol;Acc:HGNC:3557] | cgttgacgtaggttagtagggc | gtacaaaaactccgacgacga | [48] |
| US-CG205 | ENSG00000138829 | FBN2 | 5:128257909-128659185:-1 | protein_coding | fibrillin 2 [Source:HGNC Symbol;Acc:HGNC:3604] | ttttagtcgtcgtcgtaataggttc | gaaacacgtcctactatcctcg | [48] |
| US-CG206 | ENSG00000114279 | FGF12 | 3:192139395-192767764:-1 | protein_coding | fibroblast growth factor 12 [Source:HGNC Symbol;Acc:HGNC:3668] | ggttaggagttttagtcgtcgcga | aaataaaaattacccgccgaacg | [48] |
| US-CG207 | ENSG00000068078 | FGFR3 | 4:1793293-1808872:1 | protein_coding | fibroblast growth factor receptor 3 [Source:HGNC Symbol;Acc:HGNC:3690] | cgattttttagttttggtttgtc | aacaataaaaactttccgtctacg | [40] |
| US-CG208 | ENSG00000178919 | FOXE1 | 9:97853254-97856715:1 | protein_coding | forkhead box E1 [Source:HGNC Symbol;Acc:HGNC:3806] | tgttcgttatgttgtcgtttatttc | gatatatctataaattttcgtccctacg | [48] |
| US-CG209 | ENSG00000103241 | FOXF1 | 16:86510527-86515418:1 | protein_coding | forkhead box F1 [Source:HGNC Symbol;Acc:HGNC:3809] | tcgtaagtgcggcggtttttcggtc | aacgacgcaattcttcccgaaacga | [48] |
| US-CG210 | ENSG00000114861 | FOXP1 | 3:70952817-71583993:-1 | protein_coding | forkhead box P1 [Source:HGNC Symbol;Acc:HGNC:3823] | cggcgaggaagttttttcgttattttttc | ccacgactattacgaactcgtctcga | [48] |
| US-CG211 | ENSG00000114861 | FOXP1 | 3:70952817-71583993:-1 | protein_coding | forkhead box P1 [Source:HGNC Symbol;Acc:HGNC:3823] | cggtttaacggagagacgtcggtc | acgtaaacgaaatcaactaccccga | [48] |
| US-CG212 | ENSG00000107485 | GATA3 | 10:8045378-8075203:1 | protein_coding | GATA binding protein 3 [Source:HGNC Symbol;Acc:HGNC:4172] | acgattttcgatttttcgacggtaggagtttttc | gactatactcgcgccctctcgccga | [15][41] |
| US-CG213 | ENSG00000136574 | GATA4 | 8:11676959-11760002:1 | protein_coding | GATA binding protein 4 [Source:HGNC Symbol;Acc:HGNC:4173] | cgagaagaggaggttttacgcgtgtaagc | actatacgaataccacccgaccgaacg | [48] |
| US-CG214 | ENSG00000130700 | GATA5 | 20:62463497-62475970:-1 | protein_coding | GATA binding protein 5 [Source:HGNC Symbol;Acc:HGNC:15802] | ttcgaattcgagggagaggaatcgc | cgaaaacctccgaaacccgatcga | [14][42] |
| US-CG215 | ENSG00000168621 | GDNF | 5:37812677-37839686:-1 | protein_coding | glial cell derived neurotrophic factor [Source:HGNC Symbol;Acc:HGNC:4232] | gagatattcgagcgggaaggtc | aaaatcgaaaaaaatatacgacg | [48] |
| US-CG216 | ENSG00000151892 | GFRA1 | 10:116056925-116273467:-1 | protein_coding | GDNF family receptor alpha 1 [Source:HGNC Symbol;Acc:HGNC:4243] | ttgagcgagttcgttggttgac | tcgataacgacgacgacaactc | [48] |
| US-CG217 | ENSG00000141404 | GNAL | 18:11688956-11885685:1 | protein_coding | G protein subunit alpha L [Source:HGNC Symbol;Acc:HGNC:4388] | gatagcgtagcgttaagttcgagac | gaaccgaaatcgtaaacaccaaccgta | [48] |
| US-CG218 | ENSG00000203618 | GP1BB | 22:19722945-19724771:1 | protein_coding | glycoprotein Ib platelet subunit beta [Source:HGNC Symbol;Acc:HGNC:4440] | tattcgtcgggttgttttcgttc | taaccccgcgtctccgaat | [48] |
| US-CG219 | ENSG00000179399 | GPC5 | 13:91398607-92873682:1 | protein_coding | glypican 5 [Source:HGNC Symbol;Acc:HGNC:4453] | tgcgggtagtttgagtgtagcgtagc | ctccaaaacgaaaaaaaccgaacga | [48] |
| US-CG220 | ENSG00000161055 | SCGB3A1 | 5:180590103-180591540:-1 | protein_coding | secretoglobin family 3A member 1 [Source:HGNC Symbol;Acc:HGNC:18384] | cggagacgttagcggaatcgtttc | gcgacgaaattttcaaccctcg | [9] |
| US-CG221 | ENSG00000206503 | HLA-A | 6:29941260-29945884:1 | protein_coding | major histocompatibility complex, class I, A [Source:HGNC Symbol;Acc:HGNC:4931] | gggttcgagatttatttcgaagtcgc | ttaccgtcgtaaacgtcctaccgat | [48] |
| US-CG222 | ENSG00000234745 | HLA-B | 6:31269491-31357188:-1 | protein_coding | major histocompatibility complex, class I, B [Source:HGNC Symbol;Acc:HGNC:4932] | cgaatcgtttttttgttgttttcggc | cgacgcgactcctcaaatcctacg | [48] |
| US-CG223 | ENSG00000105991 | HOXA1 | 7:27092993-27095996:-1 | protein_coding | homeobox A1 [Source:HGNC Symbol;Acc:HGNC:5099] | tagtcgtcgtagtagttgtcgtcgt | gacttccgcaataataaatcaccgtt | [48] |
| US-CG224 | ENSG00000253293 | HOXA10 | 7:27170591-27180261:-1 | protein_coding | homeobox A10 [Source:HGNC Symbol;Acc:HGNC:5100] | ttgtcggatggagtcgtttgac | aaaacacgaaataacctacgacgct | [48] |
| US-CG225 | ENSG00000005073 | HOXA11 | 7:27181510-27185223:-1 | protein_coding | homeobox A11 [Source:HGNC Symbol;Acc:HGNC:5101] | gcgcgtcggtaattttaagttc | acgcaaaacgctacctttatacg | [48] |
| US-CG226 | ENSG00000105996 | HOXA2 | 7:27100354-27102811:-1 | protein_coding | homeobox A2 [Source:HGNC Symbol;Acc:HGNC:5103] | aagatttcgacgtttttatattcga | cgacaaaaatacgattttcttaaccg | [48] |
| US-CG227 | ENSG00000106004 | HOXA5 | 7:27141052-27143668:-1 | protein_coding | homeobox A5 [Source:HGNC Symbol;Acc:HGNC:5106] | ttgtttcgagaatttttcggatagc | cacgcaaaacacgaactaacgtc | [14][43] |
| US-CG228 | ENSG00000106004 | HOXA5 | 7:27141052-27143668:-1 | protein_coding | homeobox A5 [Source:HGNC Symbol;Acc:HGNC:5106] | gttcggtagcgatagttattacggc | aaacgtcctcctcgactccg | [14][43] |
| US-CG229 | ENSG00000122592 | HOXA7 | 7:27153716-27157936:-1 | protein_coding | homeobox A7 [Source:HGNC Symbol;Acc:HGNC:5108] | gcgttcgtagattttagtgc | cttttaaacgaaactcgccg | [48] |
| US-CG230 | ENSG00000180806 | HOXC9 | 12:53994895-54003337:1 | protein_coding | homeobox C9 [Source:HGNC Symbol;Acc:HGNC:5130] | gttattacgtttttaagtcggac | ctcgaacgaaaacaatatctacga | [48] |
| US-CG231 | ENSG00000122254 | HS3ST2 | 16:22814177-22916338:1 | protein_coding | heparan sulfate-glucosamine 3-sulfotransferase 2 [Source:HGNC Symbol;Acc:HGNC:5195] | gagtagtttatcgttaggcggc | gaaataaataaaaaaaccgaacgta | [48] |
| US-CG232 | ENSG00000116824 | CD2 | 1:116754385-116769228:1 | protein_coding | CD2 molecule [Source:HGNC Symbol;Acc:HGNC:1639] | cgttatcgtttatttcggttaagtc | gaaatcttcctcgatatcctacga | [48] |
| US-CG233 | ENSG00000135312 | HTR1B | 6:77461753-77463773:-1 | protein_coding | 5-hydroxytryptamine receptor 1B [Source:HGNC Symbol;Acc:HGNC:5287] | cgttttcggattttgaaatagac | cgcgaattaataaaaataaccg | [48] |
| US-CG234 | ENSG00000168310 | IRF2 | 4:184387729-184474550:-1 | protein_coding | interferon regulatory factor 2 [Source:HGNC Symbol;Acc:HGNC:6117] | cgggttagaaacgagcgaaagttatttc | accgaacccttaacgctcgactacg | [48] |
| US-CG235 | ENSG00000146674 | IGFBP3 | 7:45912245-45921874:-1 | protein_coding | insulin like growth factor binding protein 3 [Source:HGNC Symbol;Acc:HGNC:5472] | tagggagattttatttcgagagc | ctacaaaaaccaaaatatacccgac | [48] |
| US-CG236 | ENSG00000136634 | IL10 | 1:206767602-206772494:-1 | protein_coding | interleukin 10 [Source:HGNC Symbol;Acc:HGNC:5962] | aattttttgatttcgtgattcgtttatttc | aatctacctcccttatcgaacacga | [48] |
| US-CG237 | ENSG00000016402 | IL20RA | 6:136999971-137045180:-1 | protein_coding | interleukin 20 receptor subunit alpha [Source:HGNC Symbol;Acc:HGNC:6003] | gtcgtcgtttcgcgtattttggttc | gacgaaaaaccccgcataataaccgt | [48] |
| US-CG238 | ENSG00000185507 | IRF7 | 11:612553-615999:-1 | protein_coding | interferon regulatory factor 7 [Source:HGNC Symbol;Acc:HGNC:6122] | atttacgcggtgtaggtcgggagc | cgaaaaaaacgacgtcaaaaacgaa | [48] |
| US-CG239 | ENSG00000170561 | IRX2 | 5:2745845-2751662:-1 | protein_coding | iroquois homeobox 2 [Source:HGNC Symbol;Acc:HGNC:14359] | gcgtattgttcggaggaagattc | gaactaaacaaaaaccgcccg | [48] |
| US-CG240 | ENSG00000140015 | KCNH5 | 14:62699454-63102037:-1 | protein_coding | potassium voltage-gated channel subfamily H member 5 [Source:HGNC Symbol;Acc:HGNC:6254] | ttttcgtaattttgtagcggtggc | tacgacccgaaccgaacttaacg | [48] |
| US-CG241 | ENSG00000075035 | WSCD2 | 12:108129471-108250537:1 | protein_coding | WSC domain containing 2 [Source:HGNC Symbol;Acc:HGNC:29117] | ttcgttcgagcgttaggtttataggagttc | caacaacgacgacaacgataaccga | [48] |
| US-CG242 | ENSG00000130294 | KIF1A | 2:240713761-240821036:-1 | protein_coding | kinesin family member 1A [Source:HGNC Symbol;Acc:HGNC:888] | ggtgacgttatagttcggtttc | taccgctcgccgactactctacgct | [48] |
| US-CG243 | ENSG00000129451 | KLK10 | 19:51012739-51020175:-1 | protein_coding | kallikrein related peptidase 10 [Source:HGNC Symbol;Acc:HGNC:6358] | gtcgtttaggttggagtgtagtgacgc | gaaaccaaaaaatcgaaaccacg | [48] |
| US-CG244 | ENSG00000058085 | LAMC2 | 1:183186238-183244900:1 | protein_coding | laminin subunit gamma 2 [Source:HGNC Symbol;Acc:HGNC:6493] | taatcgaggcgtcgggtagc | ccgaactacgaacaaaaaaaacgaa | [48] |
| US-CG245 | ENSG00000106689 | LHX2 | 9:124001670-124033301:1 | protein_coding | LIM homeobox 2 [Source:HGNC Symbol;Acc:HGNC:6594] | ggtatatacggtttgatgtcgc | gctctatcccgttattaaaaacga | [48] |
| US-CG246 | ENSG00000121454 | LHX4 | 1:180230286-180278982:1 | protein_coding | LIM homeobox 4 [Source:HGNC Symbol;Acc:HGNC:21734] | cgtaaggcgaagatatagcgacgt | ctaccgcgacaacaaataatcgaa | [48] |
| US-CG247 | ENSG00000215475 | SIAH3 | 13:45777243-45851736:-1 | protein_coding | siah E3 ubiquitin protein ligase family member 3 [Source:HGNC Symbol;Acc:HGNC:30553] | gcgagagtgagtcgaggtaaatacgtcg | gacgaacgtatacaaccctttacaaacccg | [48] |
| US-CG248 | ENSG00000172005 | MAL | 2:95025677-95053996:1 | protein_coding | mal, T cell differentiation protein [Source:HGNC Symbol;Acc:HGNC:6817] | cgcgtagtattaagtagagaggttc | actccaaacgacgatttcctaacg | [48] |
| US-CG249 | ENSG00000101460 | MAP1LC3A | 20:34546854-34560345:1 | protein_coding | microtubule associated protein 1 light chain 3 alpha [Source:HGNC Symbol;Acc:HGNC:6838] | cgtagtcgtagtcgtcgtgtttagcgc | gtcacctacgacaatcgaaatcgaccga | [48] |
| US-CG250 | ENSG00000034152 | MAP2K3 | 17:21284672-21315240:1 | protein_coding | mitogen-activated protein kinase kinase 3 [Source:HGNC Symbol;Acc:HGNC:6843] | aaacgaaaggattaatcgggtac | cgacgactacgacgaaaactacga | [48] |
| US-CG251 | ENSG00000112559 | MDFI | 6:41636882-41654246:1 | protein_coding | MyoD family inhibitor [Source:HGNC Symbol;Acc:HGNC:6967] | tttgcgggggaatcgttttttgttc | cgctcacctaatacatcgaccccga | [48] |
| US-CG252 | ENSG00000106511 | MEOX2 | 7:15611212-15686812:-1 | protein_coding | mesenchyme homeobox 2 [Source:HGNC Symbol;Acc:HGNC:7014] | acgttacggcgtaaggtttgtattc | ctcttcgttaaaatatcccgcgat | [48] |
| US-CG253 | ENSG00000076242 | MLH1 | 3:36993332-37050918:1 | protein_coding | mutL homolog 1 [Source:HGNC Symbol;Acc:HGNC:7127] | tcgtcgttcgttatatatcgttcgt | acctaatctatcgccgcctcatcgta | [48] |
| US-CG254 | ENSG00000119684 | MLH3 | 14:75013764-75051532:-1 | protein_coding | mutL homolog 3 [Source:HGNC Symbol;Acc:HGNC:7128] | cgtttattcgttagttgttgtcgg | gataaaaccgattttccaaactcgaa | [48] |
| US-CG255 | ENSG00000095002 | MSH2 | 2:47402969-47663146:1 | protein_coding | mutS homolog 2 [Source:HGNC Symbol;Acc:HGNC:7325] | gatatttcggtatttcgggaggtc | ctacgattacaaatacgcgccg | [14][43] |
| US-CG256 | ENSG00000163132 | MSX1 | 4:4859666-4863936:1 | protein_coding | msh homeobox 1 [Source:HGNC Symbol;Acc:HGNC:7391] | tcgagaagttcgagaggatttc | gactacaaatacgcaaacgcc | [48] |
| US-CG257 | ENSG00000120149 | MSX2 | 5:174724533-174730893:1 | protein_coding | msh homeobox 2 [Source:HGNC Symbol;Acc:HGNC:7392] | gtaatgatttgttttcgttcgac | aaactaaaaaccttaacgcgacgct | [48] |
| US-CG258 | ENSG00000087250 | MT3 | 16:56589074-56591088:1 | protein_coding | metallothionein 3 [Source:HGNC Symbol;Acc:HGNC:7408] | cgggtttcggtagtgtata | aacgatacttttataacctcgaacg | [48] |
| US-CG259 | ENSG00000177000 | MTHFR | 1:11785723-11806920:-1 | protein_coding | methylenetetrahydrofolate reductase [Source:HGNC Symbol;Acc:HGNC:7436] | atttgcgttttacgtgattggtttc | cgttaaaaaccgtacctttatcgtc | [48] |
| US-CG260 | ENSG00000133454 | MYO18B | 22:25742144-26031041:1 | protein_coding | myosin XVIIIB [Source:HGNC Symbol;Acc:HGNC:18150] | gtcgttagtttcggatagtgaggc | gcgaaaaaacgactaaaacacgat | [48] |
| US-CG261 | ENSG00000103034 | NDRG4 | 16:58462846-58513628:1 | protein_coding | NDRG family member 4 [Source:HGNC Symbol;Acc:HGNC:14466] | gtattttagtcgcgtagaaggc | aatttaacgaatataaacgctcga | [48] |
| US-CG262 | ENSG00000010322 | NISCH | 3:52455118-52493071:1 | protein_coding | nischarin [Source:HGNC Symbol;Acc:HGNC:18006] | ttgtgtagggtatcgtcggc | gtacgaaatactcgcttacgt | [48] |
| US-CG263 | ENSG00000053438 | NNAT | 20:37521206-37523693:1 | protein_coding | neuronatin [Source:HGNC Symbol;Acc:HGNC:7860] | tattttttaaggcgcgtatgcg | atttaccgaaatccgctaatctcga | [48] |
| US-CG264 | ENSG00000122585 | NPY | 7:24284163-24291865:1 | protein_coding | neuropeptide Y [Source:HGNC Symbol;Acc:HGNC:7955] | gttttgaatttttaagttcgttcgt | ataatatctaaccatatcctccgct | [48] |
| US-CG265 | ENSG00000112333 | NR2E1 | 6:108166058-108188809:1 | protein_coding | nuclear receptor subfamily 2 group E member 1 [Source:HGNC Symbol;Acc:HGNC:7973] | tcgtatatggttgacgttagatttc | ccgtaactttatacctatcgctacg | [48] |
| US-CG266 | ENSG00000091129 | NRCAM | 7:108147623-108456717:-1 | protein_coding | neuronal cell adhesion molecule [Source:HGNC Symbol;Acc:HGNC:7994] | gttagtttcgtttcgttagggtc | aaccgccgatatcctccgt | [48] |
| US-CG267 | ENSG00000175352 | NRIP3 | 11:8980576-9004049:-1 | protein_coding | nuclear receptor interacting protein 3 [Source:HGNC Symbol;Acc:HGNC:1167] | gatagtagcgtcggatgaagtaggc | gtaactcaataaaaaccgcaacgac | [48] |
| US-CG268 | ENSG00000140538 | NTRK3 | 15:87859751-88256768:-1 | protein_coding | neurotrophic receptor tyrosine kinase 3 [Source:HGNC Symbol;Acc:HGNC:8033] | ggttattatgttcagtagatcgc | gccgccgaataaaaaccg | [48] |
| US-CG269 | ENSG00000184221 | OLIG1 | 21:33070144-33072420:1 | protein_coding | oligodendrocyte transcription factor 1 [Source:HGNC Symbol;Acc:HGNC:16983] | aaagcggtgttagtcgtattttc | cgacttaaaaacctacgaaacaacg | [48] |
| US-CG270 | ENSG00000183715 | OPCML | 11:132414977-133532519:-1 | protein_coding | opioid binding protein/cell adhesion molecule like [Source:HGNC Symbol;Acc:HGNC:8143] | gttttatgggggatcggaacgc | ccaaaaacacgacaatcgacacg | [44] |
| US-CG271 | ENSG00000143867 | OSR1 | 2:19351485-19358653:-1 | protein_coding | odd-skipped related transciption factor 1 [Source:HGNC Symbol;Acc:HGNC:8111] | tcgtttttttgacggattttatatc | gcgactacaacttctactaactcgaa | [48] |
| US-CG272 | ENSG00000180914 | OXTR | 3:8750408-8769628:-1 | protein_coding | oxytocin receptor [Source:HGNC Symbol;Acc:HGNC:8529] | tatatttttcgatacgtcggattc | ccataaccctaacgacaacgatacg | [48] |
| US-CG273 | ENSG00000147889 | CDKN2A | 9:21967753-21995301:-1 | protein_coding | cyclin dependent kinase inhibitor 2A [Source:HGNC Symbol;Acc:HGNC:1787] | cggttgattggttggttacggtcgc | ccgtaactattcgatacgttaaacaacg | [4][7][9] |
| US-CG274 | ENSG00000185345 | PRKN | 6:161347420-162727771:-1 | protein_coding | parkin RBR E3 ubiquitin protein ligase [Source:HGNC Symbol;Acc:HGNC:8607] | ttcgtcgaggcggtttttatgagaac | gcgaatccgaaaccccaatatatacgt | [48] |
| US-CG275 | ENSG00000196092 | PAX5 | 9:36833275-37034185:-1 | protein_coding | paired box 5 [Source:HGNC Symbol;Acc:HGNC:8619] | ttcgagggtaatgcgattttatttatgggtcg | aatccgaacttccgctttaaaacacccg | [48] |
| US-CG276 | ENSG00000007372 | PAX6 | 11:31784779-31818062:-1 | protein_coding | paired box 6 [Source:HGNC Symbol;Acc:HGNC:8620] | cgtttaatgttttcgattcgcgggaaaagac | cgcgacgaacaaattccctcgacg | [48] |
| US-CG277 | ENSG00000125618 | PAX8 | 2:113215997-113278950:-1 | protein_coding | paired box 8 [Source:HGNC Symbol;Acc:HGNC:8622] | ttggatttgtcgttttttcgttttc | ctaaatacgctaccgactcccgaa | [48] |
| US-CG278 | ENSG00000160191 | PDE9A | 21:42653636-42775509:1 | protein_coding | phosphodiesterase 9A [Source:HGNC Symbol;Acc:HGNC:8795] | taaaaagttcgagtgtagtcgtc | acaaacgcacgaacaatcccg | [48] |
| US-CG279 | ENSG00000139515 | PDX1 | 13:27920020-27926231:1 | protein_coding | pancreatic and duodenal homeobox 1 [Source:HGNC Symbol;Acc:HGNC:6107] | tttagttttcgattttcggttttc | gcgcataaatccttataaaactacg | [48] |
| US-CG280 | ENSG00000179094 | PER1 | 17:8140472-8156506:-1 | protein_coding | period circadian regulator 1 [Source:HGNC Symbol;Acc:HGNC:8845] | gcgtcgggaagagatttttagttaatc | cgaaaaaacgaattacataataccgaa | [48] |
| US-CG281 | ENSG00000165462 | PHOX2A | 11:72239077-72245664:-1 | protein_coding | paired like homeobox 2A [Source:HGNC Symbol;Acc:HGNC:691] | cgtttagagttggtttcgggttttc | tacgaattaaaataaaaataatccatcg | [48] |
| US-CG282 | ENSG00000164093 | PITX2 | 4:110617423-110642123:-1 | protein_coding | paired like homeodomain 2 [Source:HGNC Symbol;Acc:HGNC:9005] | tgacgttacgttattttttatac | ctccgactacacgacaaacg | [45] |
| US-CG283 | ENSG00000147596 | PRDM14 | 8:70051651-70071693:-1 | protein_coding | PR/SET domain 14 [Source:HGNC Symbol;Acc:HGNC:14001] | cggtttcgggatggttttatttc | ataatacgcgaccaaattctacgaa | [48] |
| US-CG284 | ENSG00000170955 | CAVIN3 | 11:6318946-6320647:-1 | protein_coding | caveolae associated protein 3 [Source:HGNC Symbol;Acc:HGNC:9400] | aagtcgtttcgttttgggtttggtc | cccgaaatcttcctcgatatcctacg | [48] |
| US-CG285 | ENSG00000171862 | PTEN | 10:87863113-87971930:1 | protein_coding | phosphatase and tensin homolog [Source:HGNC Symbol;Acc:HGNC:9588] | aatcggcggtagttagtaggc | cgacgaaaacaaaaactcga | [46] |
| US-CG286 | ENSG00000125384 | PTGER2 | 14:52314305-52328606:1 | protein_coding | prostaglandin E receptor 2 [Source:HGNC Symbol;Acc:HGNC:9594] | tcgggatttggtgttttattcggtac | aacgacttctccgacttcgacgat | [48] |
| US-CG287 | ENSG00000073756 | PTGS2 | 1:186671791-186680427:-1 | protein_coding | prostaglandin-endoperoxide synthase 2 [Source:HGNC Symbol;Acc:HGNC:9605] | tttgtagtgagcgttaggagtac | atacgtaaaaccgaaatccccgat | [48] |
| US-CG288 | ENSG00000077092 | RARB | 3:25174332-25597932:1 | protein_coding | retinoic acid receptor beta [Source:HGNC Symbol;Acc:HGNC:9865] | tcgagaacgcgagcgattc | gaccaatccaaccgaaacga | [9] |
| US-CG289 | ENSG00000122035 | RASL11A | 13:27270327-27273690:1 | protein_coding | RAS like family 11 member A [Source:HGNC Symbol;Acc:HGNC:23802] | cgcgagtagttagtagttcggcgaagcg | aaaaacttaatacgcccgaaaacgcgacga | [48] |
| US-CG290 | ENSG00000068028 | RASSF1 | 3:50329782-50340980:-1 | protein_coding | Ras association domain family member 1 [Source:HGNC Symbol;Acc:HGNC:9882] | agcgtttaaagttagcgaagtac | taacacgctccaaccgaatacg | [48] |
| US-CG291 | ENSG00000189431 | RASSF10 | 11:13009577-13012106:1 | protein_coding | Ras association domain family member 10 [Source:HGNC Symbol;Acc:HGNC:33984] | tattgtttcgttttgtttttcgtc | cttaacctcgtaataatcgatctcg | [48] |
| US-CG292 | ENSG00000101265 | RASSF2 | 20:4780023-4823645:-1 | protein_coding | Ras association domain family member 2 [Source:HGNC Symbol;Acc:HGNC:9883] | gtatttcgcgttagtgtttc | ttaaacccgacccgccgatcg | [48] |
| US-CG293 | ENSG00000101265 | RASSF2 | 20:4780023-4823645:-1 | protein_coding | Ras association domain family member 2 [Source:HGNC Symbol;Acc:HGNC:9883] | gtttttatcggatttgttcgttc | ccaacccgaaaaaatcgctaacgacg | [48] |
| US-CG294 | ENSG00000266094 | RASSF5 | 1:206507530-206589448:1 | protein_coding | Ras association domain family member 5 [Source:HGNC Symbol;Acc:HGNC:17609] | tacggattttatttttttcggttc | ataacgacaactttaacaacgacg | [48] |
| US-CG295 | ENSG00000169435 | RASSF6 | 4:73571550-73620631:-1 | protein_coding | Ras association domain family member 6 [Source:HGNC Symbol;Acc:HGNC:20796] | agataggcgtcgttttttgc | cttacttcgacgatttattctcgac | [48] |
| US-CG296 | ENSG00000123094 | RASSF8 | 12:25959029-26079892:1 | protein_coding | Ras association domain family member 8 [Source:HGNC Symbol;Acc:HGNC:13232] | tttcgttacgaggtttttttcggcg | ccgaacgctcgaatttcaaccg | [48] |
| US-CG297 | ENSG00000139687 | RB1 | 13:48303726-48599436:1 | protein_coding | RB transcriptional corepressor 1 [Source:HGNC Symbol;Acc:HGNC:9884] | tttttcggcgtttttttatagttc | aaacgacgacgactctactcg | [48] |
| US-CG298 | ENSG00000182732 | RGS6 | 14:71932439-72566529:1 | protein_coding | regulator of G protein signaling 6 [Source:HGNC Symbol;Acc:HGNC:10002] | gggcgtcgcgtttttatagtcgtc | gcgaaaatctatccgttccctcga | [48] |
| US-CG299 | ENSG00000185088 | RPS27L | 15:63125872-63158021:-1 | protein_coding | ribosomal protein S27 like [Source:HGNC Symbol;Acc:HGNC:18476] | cgagtattttgttgcggcgtttttttattatc | acaaacgatctacgctcgacatcaacttccg | [48] |
| US-CG300 | ENSG00000159216 | RUNX1 | 21:34787801-36004667:-1 | protein_coding | runt related transcription factor 1 [Source:HGNC Symbol;Acc:HGNC:10471] | gaacgggtttttttgcgtttgcgac | gacacccgacctaacaacgttcaacg | [48] |
| US-CG301 | ENSG00000169439 | SDC2 | 8:96493351-96611780:1 | protein_coding | syndecan 2 [Source:HGNC Symbol;Acc:HGNC:10659] | agttttcaagtttgagtcgtaatc | ctcctacccaacgctcga | [48] |
| US-CG302 | ENSG00000012171 | SEMA3B | 3:50267558-50277546:1 | protein_coding | semaphorin 3B [Source:HGNC Symbol;Acc:HGNC:10724] | tcgggtgttgtgttcgtattac | aattttatcgtcgtctaaattctcg | [48] |
| US-CG303 | ENSG00000184640 | SEPT9 | 17:77280569-77500596:1 | protein_coding | septin 9 [Source:HGNC Symbol;Acc:HGNC:7323] | ttttatttagttgcgcgttgatc | ccgaaaacgcttcctcg | [48] |
| US-CG304 | ENSG00000120057 | SFRP5 | 10:97766751-97771952:-1 | protein_coding | secreted frizzled related protein 5 [Source:HGNC Symbol;Acc:HGNC:10779] | aagatttggcgttgggcgggacgttc | actccaacccgaacctcgccgtacg | [9][22][47] |
| US-CG305 | ENSG00000164690 | SHH | 7:155799986-155812273:-1 | protein_coding | sonic hedgehog [Source:HGNC Symbol;Acc:HGNC:10848] | cgttgtgtatagcggcgggtaggagtc | aaacccgaaacgtcctcgaactcga | [48] |
| US-CG306 | ENSG00000168779 | SHOX2 | 3:158095954-158106503:-1 | protein_coding | short stature homeobox 2 [Source:HGNC Symbol;Acc:HGNC:10854] | cgtcgtcgagagaagtagggagtc | ccgaaaaaaatccgaaaaacga | [48] |
| US-CG307 | ENSG00000112246 | SIM1 | 6:100385015-100464929:-1 | protein_coding | SIM bHLH transcription factor 1 [Source:HGNC Symbol;Acc:HGNC:10882] | aataattttttaaatcggtttcgc | gaacctacgaattaaatctaccgac | [48] |
| US-CG308 | ENSG00000126778 | SIX1 | 14:60643415-60658259:-1 | protein_coding | SIX homeobox 1 [Source:HGNC Symbol;Acc:HGNC:10887] | tcggaggtaaagagatcgggtcgc | cgtaaacgaactccgaccgacga | [48] |
| US-CG309 | ENSG00000170577 | SIX2 | 2:45005161-45009430:-1 | protein_coding | SIX homeobox 2 [Source:HGNC Symbol;Acc:HGNC:10888] | ttggtttaagaatcggcggtagcg | gaaaaaccgacaaaaaccctacgaa | [48] |
| US-CG310 | ENSG00000138083 | SIX3 | 2:44941898-44946077:1 | protein_coding | SIX homeobox 3 [Source:HGNC Symbol;Acc:HGNC:10889] | aagttgcgcggtcgtttattcggttc | gccgtcccaaataatacgtaacaacga | [48] |
| US-CG311 | ENSG00000100625 | SIX4 | 14:60709528-60724348:-1 | protein_coding | SIX homeobox 4 [Source:HGNC Symbol;Acc:HGNC:10890] | tatcgaggtcgagcgagttcgc | gcttctcgacgaacgaaaaataacg | [48] |
| US-CG312 | ENSG00000177045 | SIX5 | 19:45764785-45769226:-1 | protein_coding | SIX homeobox 5 [Source:HGNC Symbol;Acc:HGNC:10891] | attatgaggtcgagcgggttcgc | gcttctcgtccgacgtaaaataacga | [48] |
| US-CG313 | ENSG00000184302 | SIX6 | 14:60508951-60512850:1 | protein_coding | SIX homeobox 6 [Source:HGNC Symbol;Acc:HGNC:10892] | cgagtcgagttcgaattttaagtcgc | cgaaacacactaccgaataacgacgat | [48] |
| US-CG314 | ENSG00000256870 | SLC5A8 | 12:101155493-101210238:-1 | protein_coding | solute carrier family 5 member 8 [Source:HGNC Symbol;Acc:HGNC:19119] | cgttcggaggtcgatatttggaggac | cgtaacgtatccataaccgcacgatcg | [48] |
| US-CG315 | ENSG00000185338 | SOCS1 | 16:11254405-11256200:-1 | protein_coding | suppressor of cytokine signaling 1 [Source:HGNC Symbol;Acc:HGNC:19383] | gtttttgagcgtgtacggggcgtac | gctaaaaacgaaaaaacaattccgctaacg | [48] |
| US-CG316 | ENSG00000156395 | SORCS3 | 10:104641101-105265235:1 | protein_coding | sortilin related VPS10 domain containing receptor 3 [Source:HGNC Symbol;Acc:HGNC:16699] | tatatattcggtagttcgagtcgcg | gtcgacaaaaataaaaaccccgtc | [48] |
| US-CG317 | ENSG00000131018 | SYNE1 | 6:152121684-152637801:-1 | protein_coding | spectrin repeat containing nuclear envelope protein 1 [Source:HGNC Symbol;Acc:HGNC:17089] | gtttcggtataaaggttcgttgagc | cgccgatacctaaaaaaactaaaaacg | [48] |
| US-CG318 | ENSG00000006128 | TAC1 | 7:97731908-97740472:1 | protein_coding | tachykinin precursor 1 [Source:HGNC Symbol;Acc:HGNC:11517] | gtttagttcgagcgtggggaaggtc | cgctatactccgaccccgaaacg | [48] |
| US-CG319 | ENSG00000102125 | TAZ | X:154411518-154421726:1 | protein_coding | tafazzin [Source:HGNC Symbol;Acc:HGNC:11577] | gaatttgtcgtcgggtttgggtcgttc | acgaccgcgctatctacttctcctacg | [48] |
| US-CG320 | ENSG00000089225 | TBX5 | 12:114353931-114408442:-1 | protein_coding | T-box 5 [Source:HGNC Symbol;Acc:HGNC:11604] | tgaatgtatcggttttatcgaac | atttaccgaaaaactccgaaccg | [48] |
| US-CG321 | ENSG00000105825 | TFPI2 | 7:93885396-93890991:-1 | protein_coding | tissue factor pathway inhibitor 2 [Source:HGNC Symbol;Acc:HGNC:11761] | tcgttgggtaaggcgttc | aaacgaacacccgaaccg | [48] |
| US-CG322 | ENSG00000092969 | TGFB2 | 1:218346235-218444619:1 | protein_coding | transforming growth factor beta 2 [Source:HGNC Symbol;Acc:HGNC:11768] | gcgtcgtagtggaaggtaggatcgaatc | aactaacgaaaaaaacgccgaaaacgcg | [48] |
| US-CG323 | ENSG00000163513 | TGFBR2 | 3:30606502-30694142:1 | protein_coding | transforming growth factor beta receptor 2 [Source:HGNC Symbol;Acc:HGNC:11773] | cggtgaggggtagttgaaagtc | cgacgtccaacccctaactctc | [48] |
| US-CG324 | ENSG00000144339 | TMEFF2 | 2:191949043-192195709:-1 | protein_coding | transmembrane protein with EGF like and two follistatin like domains 2 [Source:HGNC Symbol;Acc:HGNC:11867] | agttcggatttcgtttcggtagggggc | cgcgaaaaccacaaataaactcgctcg | [19] |
| US-CG325 | ENSG00000182985 | CADM1 | 11:115169218-115504957:-1 | protein_coding | cell adhesion molecule 1 [Source:HGNC Symbol;Acc:HGNC:5951] | agcgaggtttttcgagagtc | actaacgacgttaactacctccg | [48] |
| US-CG326 | ENSG00000026025 | VIM | 10:17228259-17237593:1 | protein_coding | vimentin [Source:HGNC Symbol;Acc:HGNC:12692] | tcgtttcgaggttttcgcgttagagac | cgactaaaactcgaccgactcgcga | [19][25] |
| US-CG327 | ENSG00000174963 | ZIC4 | 3:147386046-147406860:-1 | protein_coding | Zic family member 4 [Source:HGNC Symbol;Acc:HGNC:20393] | ggtcgggttaaacgtaaagtagtc | aaaaactaaacgacgaaaaaatacg | [48] |
| US-CG328 | ENSG00000170954 | ZNF415 | 19:53107879-53133077:-1 | protein_coding | zinc finger protein 415 [Source:HGNC Symbol;Acc:HGNC:20636] | gtatgcgtagttttttataggttcggaaac | cacaaaaaatcactcacgctcgacgc | [48] |
| US-CG329 | ENSG00000186017 | ZNF566 | 19:36445119-36489902:-1 | protein_coding | zinc finger protein 566 [Source:HGNC Symbol;Acc:HGNC:25919] | ttagcgcgtgagttggaagttgaattatcgc | cgatcgaaaaccatcttatccgaccttaatacgt | [48] |
| US-CG330 | ENSG00000161551 | ZNF577 | 19:51804816-51890950:-1 | protein_coding | zinc finger protein 577 [Source:HGNC Symbol;Acc:HGNC:28673] | tgtcgtatttcgtgttttc | acccacgcaaaccaaaccg | [48] |
| US-CG331 | ENSG00000197928 | ZNF677 | 19:53235381-53254898:-1 | protein_coding | zinc finger protein 677 [Source:HGNC Symbol;Acc:HGNC:28730] | cgacgggattgggaacgttttagggttac | cgctccgctatcgaattttaaaatcgc | [48] |
| US-CG332 | ENSG00000165556 | CDX2 | 13:27962137-27971139:-1 | protein_coding | caudal type homeobox 2 [Source:HGNC Symbol;Acc:HGNC:1806] | ttgcgtttcgacgtttttaat | cgccacaaactaacgtacg | [48] |
| US-CG333 | ENSG00000165556 | CDX2 | 13:27962137-27971139:-1 | protein_coding | caudal type homeobox 2 [Source:HGNC Symbol;Acc:HGNC:1806] | ataggatgaaggcgatggtg | actacgacgaaacccgacg | [48] |
| US-CG334 | ENSG00000165556 | CDX2 | 13:27962137-27971139:-1 | protein_coding | caudal type homeobox 2 [Source:HGNC Symbol;Acc:HGNC:1806] | ttcggaggtagaagagtcgc | cgtacataataacgaaaatccg | [48] |
| US-CG335 | ENSG00000138061 | CYP1B1 | 2:38066973-38109902:-1 | protein_coding | cytochrome P450 family 1 subfamily B member 1 [Source:HGNC Symbol;Acc:HGNC:2597] | ttcgttttgttaggtcgc | aaactaaaaatcgataaataacg | [48] |
| US-CG336 | ENSG00000138061 | CYP1B1 | 2:38066973-38109902:-1 | protein_coding | cytochrome P450 family 1 subfamily B member 1 [Source:HGNC Symbol;Acc:HGNC:2597] | tttcggagtcggttgtttc | aacaacgctacgaacatcg | [48] |
| US-CG81 | ENSG00000188157 | AGRN | 1:1020123-1056118:1 | protein_coding | agrin [Source:HGNC Symbol;Acc:HGNC:329] | gtagtgacgttagggttcgga | cccatacaaattacgacgaa | TCGA &GEO analysis |
| US-CG82 | ENSG00000174945 | AMZ1 | 7:2679522-2775500:1 | protein_coding | archaelysin family metallopeptidase 1 [Source:HGNC Symbol;Acc:HGNC:22231] | tatttggtacgtggacgaag | accttctactactaaaaacctaaccgaa | TCGA &GEO analysis |
| US-CG83 | ENSG00000174945 | AMZ1 | 7:2679522-2775500:1 | protein_coding | archaelysin family metallopeptidase 1 [Source:HGNC Symbol;Acc:HGNC:22231] | gtcgagtaaatacggcggtt | aattaatcttacgaaaactcgaac | TCGA &GEO analysis |
| US-CG84 | ENSG00000136457 | CHAD | 17:50464496-50468966:-1 | protein_coding | chondroadherin [Source:HGNC Symbol;Acc:HGNC:1909] | gggttatcgtataaagagggc | ttctaaaaacaaacgaccaacg | TCGA &GEO analysis |
| US-CG85 | ENSG00000136457 | CHAD | 17:50464496-50468966:-1 | protein_coding | chondroadherin [Source:HGNC Symbol;Acc:HGNC:1909] | ttcggggttcgggggttacg | cctaaccgcaaccgacaaccga | TCGA &GEO analysis |
| US-CG86 | ENSG00000122966 | CIT | 12:119685790-119877291:-1 | protein_coding | citron rho-interacting serine/threonine kinase [Source:HGNC Symbol;Acc:HGNC:1985] | gggaaggtagtttgtttgcg | gctaacgataacactacaacgc | TCGA &GEO analysis |
| US-CG87 | ENSG00000205560 | CPT1B | 22:50568861-50578465:-1 | protein_coding | carnitine palmitoyltransferase 1B [Source:HGNC Symbol;Acc:HGNC:2329] | tcgagttgtgagtaggggtc | aaacaaaattcgaaaacccg | TCGA &GEO analysis |
| US-CG88 | ENSG00000187553 | CYP26C1 | 10:93060798-93069540:1 | protein_coding | cytochrome P450 family 26 subfamily C member 1 [Source:HGNC Symbol;Acc:HGNC:20577] | taagggaatatgatgagttcgc | caaaccttttacgaacgaa | TCGA &GEO analysis |
| US-CG89 | ENSG00000213722 | DDAH2 | 6:31727038-31730617:-1 | protein_coding | dimethylarginine dimethylaminohydrolase 2 [Source:HGNC Symbol;Acc:HGNC:2716] | gacgttggatggtattgac | ccgcgaaaatttaaaaacg | TCGA &GEO analysis |
| US-CG90 | ENSG00000069696 | DRD4 | 11:637293-640706:1 | protein_coding | dopamine receptor D4 [Source:HGNC Symbol;Acc:HGNC:3025] | acgggttgttggttgggc | taaacgtctacaaaacgcgctc | TCGA &GEO analysis |
| US-CG91 | ENSG00000004776 | HSPB6 | 19:35754569-35758079:-1 | protein_coding | heat shock protein family B (small) member 6 [Source:HGNC Symbol;Acc:HGNC:26511] | gtagttaagtaggagggggttgggtc | caatccttaacgcactcgaaa | TCGA &GEO analysis |
| US-CG92 | ENSG00000170049 | KCNAB3 | 17:7921859-7929803:-1 | protein_coding | potassium voltage-gated channel subfamily A regulatory beta subunit 3 [Source:HGNC Symbol;Acc:HGNC:6230] | ggttttatgtagatgaggaggc | gaaccgctacgaaacccg | TCGA &GEO analysis |
| US-CG93 | ENSG00000204428 | LY6G5C | 6:31676684-31684040:-1 | protein_coding | lymphocyte antigen 6 family member G5C [Source:HGNC Symbol;Acc:HGNC:13932] | taggagaggagtaaaacgtaataaagacgg | cgaaacaaaataaaaccttcttcg | TCGA &GEO analysis |
| US-CG94 | ENSG00000143995 | MEIS1 | 2:66433452-66573869:1 | protein_coding | Meis homeobox 1 [Source:HGNC Symbol;Acc:HGNC:7000] | gttcgggataagatttcgggg | taattaaaactacgcaacccgact | TCGA &GEO analysis |
| US-CG95 | ENSG00000148826 | NKX6-2 | 10:132783179-132786052:-1 | protein_coding | NK6 homeobox 2 [Source:HGNC Symbol;Acc:HGNC:19321] | agaagaagtattcgcgttcgat | gatcatacccaacgaataaacg | TCGA &GEO analysis |
| US-CG96 | ENSG00000115507 | OTX1 | 2:63050057-63057836:1 | protein_coding | orthodenticle homeobox 1 [Source:HGNC Symbol;Acc:HGNC:8521] | gttagtagtagtagagcgggagc | gacgtaaattaaccactactttcg | TCGA &GEO analysis |
| US-CG97 | ENSG00000115507 | OTX1 | 2:63050057-63057836:1 | protein_coding | orthodenticle homeobox 1 [Source:HGNC Symbol;Acc:HGNC:8521] | tttatcgcgggtttacgtggt | gcctcgaacacgtccaacta | TCGA &GEO analysis |
| US-CG98 | ENSG00000069011 | PITX1 | 5:135027735-135034813:-1 | protein_coding | paired like homeodomain 1 [Source:HGNC Symbol;Acc:HGNC:9004] | gttggattcggattgagtggtc | aacaacttaatttatacgaaatcgaaa | TCGA &GEO analysis |
| US-CG99 | ENSG00000104881 | PPP1R13L | 19:45379634-45406349:-1 | protein_coding | protein phosphatase 1 regulatory subunit 13 like [Source:HGNC Symbol;Acc:HGNC:18838] | gtatcgtagatgttagtcgcgg | ataccctcctcgttaaactaactc | TCGA &GEO analysis |
| US-CG100 | ENSG00000204314 | PRRT1 | 6:32148359-32153083:-1 | protein_coding | proline rich transmembrane protein 1 [Source:HGNC Symbol;Acc:HGNC:13943] | cgcggatttaatgggcgt | cgtttaaccgaaatcccgaaa | TCGA &GEO analysis |
| US-CG101 | ENSG00000142949 | PTPRF | 1:43525187-43623666:1 | protein_coding | protein tyrosine phosphatase, receptor type F [Source:HGNC Symbol;Acc:HGNC:9670] | gtagaggttttatttaggcggg | cgataaccataacgacgataaaac | TCGA &GEO analysis |
| US-CG102 | ENSG00000100918 | REC8 | 14:24171853-24180257:1 | protein_coding | REC8 meiotic recombination protein [Source:HGNC Symbol;Acc:HGNC:16879] | gtttattggttaaggaaggggcgt | aaacgaaaatcgaatcgaaaacg | TCGA &GEO analysis |
| US-CG103 | ENSG00000132005 | RFX1 | 19:13961538-14007039:-1 | protein_coding | regulatory factor X1 [Source:HGNC Symbol;Acc:HGNC:9982] | ttttcgttaggaaggtagagtagcgaga | gtaataaaacgacgacgactcgaaa | TCGA &GEO analysis |
| US-CG104 | ENSG00000204618 | RNF39 | 6:30070266-30075887:-1 | protein_coding | ring finger protein 39 [Source:HGNC Symbol;Acc:HGNC:18064] | ggtcggggaattagtgtaacgta | gtaaaaacccacaaacgacc | TCGA &GEO analysis |
| US-CG105 | ENSG00000204618 | RNF39 | 6:30070266-30075887:-1 | protein_coding | ring finger protein 39 [Source:HGNC Symbol;Acc:HGNC:18064] | ggtcggggaattagtgtaacgta | gaataccgtaaaaacccacaaacg | TCGA &GEO analysis |
| US-CG106 | ENSG00000172426 | RSPH9 | 6:43645046-43672599:1 | protein_coding | radial spoke head 9 homolog [Source:HGNC Symbol;Acc:HGNC:21057] | ttagttaggcggaagggc | aaatcaacgactccgctc | TCGA &GEO analysis |
| US-CG107 | ENSG00000159263 | SIM2 | 21:36699133-36749917:1 | protein_coding | SIM bHLH transcription factor 2 [Source:HGNC Symbol;Acc:HGNC:10883] | gtaggcgtagaggggataattcg | accccgcgctaaatctacaac | TCGA &GEO analysis |
| US-CG108 | ENSG00000157637 | SLC38A10 | 17:81245000-81295547:-1 | protein_coding | solute carrier family 38 member 10 [Source:HGNC Symbol;Acc:HGNC:28237] | cgggggttattagatgatcg | aaaaacatacgttcccgacg | TCGA &GEO analysis |
| US-CG109 | ENSG00000182968 | SOX1 | 13:112067647-112070488:1 | protein_coding | SRY-box 1 [Source:HGNC Symbol;Acc:HGNC:11189] | taattaggatcgggttaaacggt | aaacgcttactaatctccgaat | TCGA &GEO analysis |
| US-CG110 | ENSG00000138162 | TACC2 | 10:121989174-122254545:1 | protein_coding | transforming acidic coiled-coil containing protein 2 [Source:HGNC Symbol;Acc:HGNC:11523] | tatatataggagtcggttgtcgggg | cgtacgaatataaacgtaaacgccg | TCGA &GEO analysis |
| US-CG111 | ENSG00000178913 | TAF7 | 5:141260225-141320821:-1 | protein_coding | TATA-box binding protein associated factor 7 [Source:HGNC Symbol;Acc:HGNC:11541] | ggtgagagtttagaataggcgg | cgactaactccccaaaacgtaa | TCGA &GEO analysis |
| US-CG112 | ENSG00000116819 | TFAP2E | 1:35573370-35595328:1 | protein_coding | transcription factor AP-2 epsilon [Source:HGNC Symbol;Acc:HGNC:30774] | tagtttagtggggagtgaattagc | ctataaaacaaaacaaaaattcctcgta | TCGA &GEO analysis |
| US-CG113 | ENSG00000182223 | ZAR1 | 4:48490252-48494389:1 | protein_coding | zygote arrest 1 [Source:HGNC Symbol;Acc:HGNC:20436] | ggttagggtataggggagttc | cgcgatcattaactatctcgaa | TCGA &GEO analysis |
| US-CG114 | ENSG00000179909 | ZNF154 | 19:57697367-57709194:-1 | protein_coding | zinc finger protein 154 [Source:HGNC Symbol;Acc:HGNC:12939] | tttatcggattagagatagtagagcg | gacattaataaaaacccgaaa | TCGA &GEO analysis |
| US-CG115 | ENSG00000187553 | CYP26C1 | 10:93060808-93069536:1 | protein_coding | cytochrome P450 family 26 subfamily C member 1 [Source:HGNC Symbol;Acc:HGNC:20577] | gacggaataggtgagtattgc | aaaaaacataataaacccgcga | TCGA &GEO analysis |
| US-CG116 | ENSG00000077549 | CAPZB | 1:19338776-19485539:-1 | protein_coding | capping actin protein of muscle Z-line subunit beta [Source:HGNC Symbol;Acc:HGNC:1491] | gagtgcgcgattagagacggg | atcgaaatacgctaaaccgcacg | TCGA &GEO analysis |
| US-CG116-2 | ENSG00000077549 | CAPZB | 1:19338776-19485539:-1 | protein_coding | capping actin protein of muscle Z-line subunit beta [Source:HGNC Symbol;Acc:HGNC:1491] | ttacggcgttgttagttggaatc | atcgaaatacgctaaaccgcacg | TCGA &GEO analysis |
| US-CG117 | ENSG00000108669 | CYTH1 | 17:78674048-78782297:-1 | protein_coding | cytohesin 1 [Source:HGNC Symbol;Acc:HGNC:9501] | tggggaaggttggcgtagagcg | acgaaacaacacacactcgaaacgtc | TCGA &GEO analysis |
| US-CG117-2 | ENSG00000108669 | CYTH1 | 17:78674048-78782297:-1 | protein_coding | cytohesin 1 [Source:HGNC Symbol;Acc:HGNC:9501] | tggcgtagagcgtagagatggacgc | acgaaacaacacacactcgaaacg | TCGA &GEO analysis |
| US-CG118 | ENSG00000079805 | DNM2 | 19:10718079-10833488:1 | protein_coding | dynamin 2 [Source:HGNC Symbol;Acc:HGNC:2974] | gatttttcggcggaatcgtattg | aaccctatcatatcacgcgaccg | TCGA &GEO analysis |
| US-CG118-2 | ENSG00000079805 | DNM2 | 19:10718079-10833488:1 | protein_coding | dynamin 2 [Source:HGNC Symbol;Acc:HGNC:2974] | cggttattgatttttcggcggaatc | aaccctatcatatcacgcgaccg | TCGA &GEO analysis |
| US-CG119 | ENSG00000113648 | H2AFY | 5:135333900-135399914:-1 | protein_coding | H2A histone family member Y [Source:HGNC Symbol;Acc:HGNC:4740] | acgtggcgggttttgtggc | gcttcaacaaaaattcactttcgaaccc | TCGA &GEO analysis |
| US-CG120 | ENSG00000054282 | SDCCAG8 | 1:243256034-243500092:1 | protein_coding | serologically defined colon cancer antigen 8 [Source:HGNC Symbol;Acc:HGNC:10671] | ttttcgggtttcgtagtcgtc | actctctcaaaacgacaatcgcc | TCGA &GEO analysis |
| US-CG120-2 | ENSG00000054282 | SDCCAG8 | 1:243256034-243500092:1 | protein_coding | serologically defined colon cancer antigen 8 [Source:HGNC Symbol;Acc:HGNC:10671] | cgttcgttttagggcgattgtc | accgcataaaaaacgtatataattcg | TCGA &GEO analysis |
| US-CG111-2 | ENSG00000178913 | TAF7 | 5:141260225-141320821:-1 | protein_coding | TATA-box binding protein associated factor 7 [Source:HGNC Symbol;Acc:HGNC:11541] | cgtaggaacgttagtgagtcgc | aaaaccgctcaaccctacgc | TCGA &GEO analysis |
| US-CG121 | ENSG00000137203 | TFAP2A | 6:10393186-10419659:-1 | protein_coding | transcription factor AP-2 alpha [Source:HGNC Symbol;Acc:HGNC:11742] | gggtagtttcggagattcgg | cctccgacgaataacaactccg | TCGA &GEO analysis |
| US-CG121-2 | ENSG00000137203 | TFAP2A | 6:10393186-10419659:-1 | protein_coding | transcription factor AP-2 alpha [Source:HGNC Symbol;Acc:HGNC:11742] | agagggtagtttcggagattc | tccgacgaataacaactccg | TCGA &GEO analysis |
| US-CG122 | ENSG00000160293 | VAV2 | 9:133761894-133992604:-1 | protein_coding | vav guanine nucleotide exchange factor 2 [Source:HGNC Symbol;Acc:HGNC:12658] | gttttgggatatggtcggaggcg | caaccgtccgaaactcgaac | TCGA &GEO analysis |
| US-CG122-2 | ENSG00000160293 | VAV2 | 9:133761894-133992604:-1 | protein_coding | vav guanine nucleotide exchange factor 2 [Source:HGNC Symbol;Acc:HGNC:12658] | ttttgggatatggtcggaggc | caaccgtccgaaactcgaac | TCGA &GEO analysis |
| US-CG351 | ENSG00000204618 | RNF39 | 6:30070266-30075887:-1 | protein_coding | ring finger protein 39 [Source:HGNC Symbol;Acc:HGNC:18064] | gtcggggaattagtgtaacg | ccgcccaacaaaataaattcg | TCGA &GEO analysis |
| US-CG352 | ENSG00000115507 | OTX1 | 2:63050057-63057836:1 | protein_coding | orthodenticle homeobox 1 [Source:HGNC Symbol;Acc:HGNC:8521] | cgattatagtggtttcgagtcg | cgaaaacacaccttaaactcg | TCGA &GEO analysis |
| US-CG353 | ENSG00000115507 | OTX1 | 2:63050057-63057836:1 | protein_coding | orthodenticle homeobox 1 [Source:HGNC Symbol;Acc:HGNC:8521] | ggtttgttaggggtttgttcg | ctacttccgcgaaataactacg | TCGA &GEO analysis |
| US-CG354 | ENSG00000115507 | OTX1 | 2:63050057-63057836:1 | protein_coding | orthodenticle homeobox 1 [Source:HGNC Symbol;Acc:HGNC:8521] | ttcgttaggtttagcgttcg | gataacgacgcctacaactacg | TCGA &GEO analysis |
| US-CG355 | ENSG00000115507 | OTX1 | 2:63050057-63057836:1 | protein_coding | orthodenticle homeobox 1 [Source:HGNC Symbol;Acc:HGNC:8521] | cgcgtatagagtcgtatttcg | aacgaacctaactatatacaacg | TCGA &GEO analysis |
| US-CG356 | ENSG00000136457 | CHAD | 17:50464496-50468966:-1 | protein_coding | chondroadherin [Source:HGNC Symbol;Acc:HGNC:1909] | gttttacgtaggagttggttcg | ctaaacgctcgaatctaccg | TCGA &GEO analysis |
| US-CG357 | ENSG00000179909 | ZNF154 | 19:57697367-57709194:-1 | protein_coding | zinc finger protein 154 [Source:HGNC Symbol;Acc:HGNC:12939] | gttaggtttgggatagggatcg | cgctaccatcaaactctacg | TCGA &GEO analysis |
| US-CG358 | ENSG00000179909 | ZNF154 | 19:57697367-57709194:-1 | protein_coding | zinc finger protein 154 [Source:HGNC Symbol;Acc:HGNC:12939] | ggatagggatcgttgttttcg | ccgcactcacctaaattaacg | TCGA &GEO analysis |
| US-CG359 | ENSG00000138162 | TACC2 | 10:121989174-122254545:1 | protein_coding | transforming acidic coiled-coil containing protein 2 [Source:HGNC Symbol;Acc:HGNC:11523] | gtatatatacgcggcgttcg | caactacgaacatccgtaacg | TCGA &GEO analysis |
| US-CG360 | ENSG00000143995 | MEIS1 | 2:66433452-66573869:1 | protein_coding | Meis homeobox 1 [Source:HGNC Symbol;Acc:HGNC:7000] | attggttcgggataagatttcg | cccgcgctaattaaaactacg | TCGA &GEO analysis |
| US-CG361 | ENSG00000143995 | MEIS1 | 2:66433452-66573869:1 | protein_coding | Meis homeobox 1 [Source:HGNC Symbol;Acc:HGNC:7000] | tggagagttggaaattttcg | aacgtaaaacccgaaccg | TCGA &GEO analysis |
| US-CG362 | ENSG00000143995 | MEIS1 | 2:66433452-66573869:1 | protein_coding | Meis homeobox 1 [Source:HGNC Symbol;Acc:HGNC:7000] | tttagaggttaggggaagcg | tctccctctactccgaaatcg | TCGA &GEO analysis |
| US-CG363 | ENSG00000140932 | CMTM2 | 16:66579448-66588275:1 | protein_coding | CKLF like MARVEL transmembrane domain containing 2 [Source:HGNC Symbol;Acc:HGNC:19173] | cgttgttttgtgtgaaagtcg | actacgaacaccgaatcacg | TCGA &GEO analysis |
| US-CG364 | ENSG00000140932 | CMTM2 | 16:66579448-66588275:1 | protein_coding | CKLF like MARVEL transmembrane domain containing 2 [Source:HGNC Symbol;Acc:HGNC:19173] | aggattcggtcgttgtcg | aatacctaatatccaccgaacg | TCGA &GEO analysis |
| US-CG365 | ENSG00000204428 | LY6G5C | 6:31676684-31684040:-1 | protein_coding | lymphocyte antigen 6 family member G5C [Source:HGNC Symbol;Acc:HGNC:13932] | tggataggagaggagtaaaacg | ccgaaacaaaataaaaccttcttcg | TCGA &GEO analysis |
| US-CG366 | ENSG00000100918 | REC8 | 14:24171853-24180257:1 | protein_coding | REC8 meiotic recombination protein [Source:HGNC Symbol;Acc:HGNC:16879] | ttggttgttagggtaaaagtcg | ccgcctactacaaaactaaatcg | TCGA &GEO analysis |
| US-CG367 | ENSG00000069011 | PITX1 | 5:135027735-135034813:-1 | protein_coding | paired like homeodomain 1 [Source:HGNC Symbol;Acc:HGNC:9004] | gggtttcggaggtttatttcg | cactcaatccgaatccaacg | TCGA &GEO analysis |
| US-CG368 | ENSG00000069011 | PITX1 | 5:135027735-135034813:-1 | protein_coding | paired like homeodomain 1 [Source:HGNC Symbol;Acc:HGNC:9004] | atgagtatgagggaggagatcg | ctacgcgaatacccttaaacg | TCGA &GEO analysis |
| US-CG369 | ENSG00000204314 | PRRT1 | 6:32148359-32153083:-1 | protein_coding | proline rich transmembrane protein 1 [Source:HGNC Symbol;Acc:HGNC:13943] | ttttaggtcggtaggtttcg | ccaaaactcgatccttttaccg | TCGA &GEO analysis |
| US-CG370 | ENSG00000204314 | PRRT1 | 6:32148359-32153083:-1 | protein_coding | proline rich transmembrane protein 1 [Source:HGNC Symbol;Acc:HGNC:13943] | gtttcgtttggtttagttttcg | actcgatctctctctactctcg | TCGA &GEO analysis |
| US-CG371 | ENSG00000159263 | SIM2 | 21:36699133-36749917:1 | protein_coding | SIM bHLH transcription factor 2 [Source:HGNC Symbol;Acc:HGNC:10883] | gggatttaggagggaaagttcg | ccgcgctaaatctacaacg | TCGA &GEO analysis |
| US-CG372 | ENSG00000159263 | SIM2 | 21:36699133-36749917:1 | protein_coding | SIM bHLH transcription factor 2 [Source:HGNC Symbol;Acc:HGNC:10883] | taggttcgtataggcgttcg | aaccgccatctatactctcg | TCGA &GEO analysis |
| US-CG373 | ENSG00000069696 | DRD4 | 11:637293-640706:1 | protein_coding | dopamine receptor D4 [Source:HGNC Symbol;Acc:HGNC:3025] | aggggcgtttatttagtttacg | caattaatcctcgcgtaaccg | TCGA &GEO analysis |
| US-CG374 | ENSG00000069696 | DRD4 | 11:637293-640706:1 | protein_coding | dopamine receptor D4 [Source:HGNC Symbol;Acc:HGNC:3025] | tatcgagcgcgttttgtagacg | gcgactcacctcgaaataaacg | TCGA &GEO analysis |
| US-CG375 | ENSG00000137203 | TFAP2A | 6:10393186-10419659:-1 | protein_coding | transcription factor AP-2 alpha [Source:HGNC Symbol;Acc:HGNC:11742] | ttacggtttcggtagtttcg | cactacttttaacgttattatccg | TCGA &GEO analysis |
| US-CG376 | ENSG00000116213 | WRAP73 | 1:3630767-3652761:-1 | protein_coding | WD repeat containing, antisense to TP73 [Source:HGNC Symbol;Acc:HGNC:12759] | cgatattcgtcgtttcgttcg | gaaactcgcccaacatatcg | TCGA &GEO analysis |
| US-CG377 | ENSG00000137166 | FOXP4 | 6:41546426-41602384:1 | protein_coding | forkhead box P4 [Source:HGNC Symbol;Acc:HGNC:20842] | gtaggtggaattcgttattttcg | acgaacgaatacgcaacg | TCGA &GEO analysis |
| US-CG378 | ENSG00000007372 | PAX6 | 11:31784779-31818062:-1 | protein_coding | paired box 6 [Source:HGNC Symbol;Acc:HGNC:8620] | gatagggagtaaagcgttcg | acgaattcgaaacccgtacg | TCGA &GEO analysis |
| US-CG379 | ENSG00000007372 | PAX6 | 11:31784779-31818062:-1 | protein_coding | paired box 6 [Source:HGNC Symbol;Acc:HGNC:8620] | gtagggttttgtgtcgttcg | cgcccactaacgataattatacg | TCGA &GEO analysis |
| US-CG380 | ENSG00000184361 | SPATA32 | 17:45254393-45262112:-1 | protein_coding | spermatogenesis associated 32 [Source:HGNC Symbol;Acc:HGNC:26349] | gaggaaggagttttggtttcg | atatcgccgcctctataacg | TCGA &GEO analysis |
| US-CG381 | ENSG00000237941 | KCNQ1DN | 11:2870033-2872105:1 | antisense | KCNQ1 downstream neighbor [Source:HGNC Symbol;Acc:HGNC:13335] | atgtatcgacggacgtatcg | ctactacaaccgcaaaaccg | TCGA &GEO analysis |
| US-CG382 | ENSG00000077327 | SPAG6 | 10:22345445-22454224:1 | protein_coding | sperm associated antigen 6 [Source:HGNC Symbol;Acc:HGNC:11215] | cgtttagtgcgtttaggtatcg | aacctaccgaaaccgaaacg | TCGA &GEO analysis |
| US-CG383 | ENSG00000204514 | ZNF814 | 19:57848731-57889074:-1 | protein_coding | zinc finger protein 814 [Source:HGNC Symbol;Acc:HGNC:33258] | ttgggttaggatacggatcg | aaccccacaattacctaaacg | TCGA &GEO analysis |
| US-CG384 | ENSG00000184640 | SEPT9 | 17:77280569-77500596:1 | protein_coding | septin 9 [Source:HGNC Symbol;Acc:HGNC:7323] | ggatagtcgcgttttgttcg | cgacctaaactatatccgttcg | TCGA &GEO analysis |
| US-CG385 | ENSG00000075826 | SEC31B | 10:100486642-100519864:-1 | protein_coding | SEC31 homolog B, COPII coat complex component [Source:HGNC Symbol;Acc:HGNC:23197] | cgtagtcgtcgtttatagcg | cgaaacgaaccaacctatacg | TCGA &GEO analysis |
| US-CG386 | ENSG00000010295 | IFFO1 | 12:6538375-6556083:-1 | protein_coding | intermediate filament family orphan 1 [Source:HGNC Symbol;Acc:HGNC:24970] | tcggaaaggtttagggttcg | gccgacgaaacaaaaccg | TCGA &GEO analysis |
| US-CG387 | ENSG00000197140 | ADAM32 | 8:39106990-39284911:1 | protein_coding | ADAM metallopeptidase domain 32 [Source:HGNC Symbol;Acc:HGNC:15479] | gtttggtgtaggagagtatacg | aacgccctctatcgaaatcg | TCGA &GEO analysis |
| US-CG389 | ENSG00000219891 | ZSCAN12P1 | 6:28091154-28093664:1 | transcribed_unprocessed_pseudogene | zinc finger and SCAN domain containing 12 pseudogene 1 [Source:HGNC Symbol;Acc:HGNC:13850] | ttagaggagtcggaagtatcg | cgaacgccacaaaacaatcg | TCGA &GEO analysis |
| US-CG390 | ENSG00000180336 | MEIOC | 17:44656404-44690308:1 | protein_coding | meiosis specific with coiled-coil domain [Source:HGNC Symbol;Acc:HGNC:26670] | gcgggaaaagtttagaagcg | ccgatacccatccaataaacg | TCGA &GEO analysis |
| US-CG391 | ENSG00000156395 | SORCS3 | 10:104641101-105265235:1 | protein_coding | sortilin related VPS10 domain containing receptor 3 [Source:HGNC Symbol;Acc:HGNC:16699] | ttagtcgcgttttcggttcg | tacgtacgcgcaaacaaacg | TCGA &GEO analysis |
| US-CG392 | ENSG00000020633 | RUNX3 | 1:24899511-24965121:-1 | protein_coding | runt related transcription factor 3 [Source:HGNC Symbol;Acc:HGNC:10473] | gaggtttagtacgcgttcg | cccgcctcctaaatctatcg | TCGA &GEO analysis |
| US-CG393 | ENSG00000167525 | PROCA1 | 17:28703197-28711854:-1 | protein_coding | protein interacting with cyclin A1 [Source:HGNC Symbol;Acc:HGNC:28600] | gaggtttcggatgaagaacg | taaccccgcctcatactaacg | TCGA &GEO analysis |
| US-CG394 | ENSG00000256463 | SALL3 | 18:78980275-79002677:1 | protein_coding | spalt like transcription factor 3 [Source:HGNC Symbol;Acc:HGNC:10527] | ttttcgtcggtcgtttcg | cgccgaaacatactactaacg | TCGA &GEO analysis |
| US-CG395 | ENSG00000227214 | HCG15 | 6:28986203-28987484:1 | antisense | HLA complex group 15 [Source:HGNC Symbol;Acc:HGNC:18361] | ttttcgtacggcgtttcg | aacgctataattcgcaaattcg | TCGA &GEO analysis |
| US-CG396 | ENSG00000204625 | HCG9 | 6:29975112-29978410:1 | lincRNA | HLA complex group 9 [Source:HGNC Symbol;Acc:HGNC:21243] | atcggtagcgtttagttttcg | ccctcgactaaaacgaaaacg | TCGA &GEO analysis |
| US-CG397 | ENSG00000144355 | DLX1 | 2:172084740-172089677:1 | protein_coding | distal-less homeobox 1 [Source:HGNC Symbol;Acc:HGNC:2914] | ggagtttcgcgaggttcg | cactctctactaccactactcg | TCGA &GEO analysis |
| US-CG398 | ENSG00000169427 | KCNK9 | 8:139600838-139704109:-1 | protein_coding | potassium two pore domain channel subfamily K member 9 [Source:HGNC Symbol;Acc:HGNC:6283] | gatttataggggtatcgggtttcg | aaaacgaccccgctaaataccg | TCGA &GEO analysis |
| US-CG399 | ENSG00000164438 | TLX3 | 5:171309284-171312134:1 | protein_coding | T cell leukemia homeobox 3 [Source:HGNC Symbol;Acc:HGNC:13532] | gatatagagtttgtcgggttcg | acgactaaatccccgttacg | TCGA &GEO analysis |
| US-CG400 | ENSG00000162105 | SHANK2 | 11:70467856-71252577:-1 | protein_coding | SH3 and multiple ankyrin repeat domains 2 [Source:HGNC Symbol;Acc:HGNC:14295] | tgagtataaaagtagagtagttcg | aaaaccaactacacgtaaacg | TCGA &GEO analysis |
| US-CG401 | ENSG00000148826 | NKX6-2 | 10:132783179-132786052:-1 | protein_coding | NK6 homeobox 2 [Source:HGNC Symbol;Acc:HGNC:19321] | ataggttcgagagaggttacg | cgctccgacgtctataataacg | TCGA &GEO analysis |
| US-CG402 | ENSG00000139865 | TTC6 | 14:37595847-38041442:1 | protein_coding | tetratricopeptide repeat domain 6 [Source:HGNC Symbol;Acc:HGNC:19739] | cggcggttttaggtaagattcg | aaccacgaatctaaccgtaccg | TCGA &GEO analysis |
| US-CG403 | ENSG00000139800 | ZIC5 | 13:99962964-99971909:-1 | protein_coding | Zic family member 5 [Source:HGNC Symbol;Acc:HGNC:20322] | ggacgtgtggttttagcg | aacaataacctcgactactcg | TCGA &GEO analysis |
| US-CG404 | ENSG00000168243 | GNG4 | 1:235547687-235650754:-1 | protein_coding | G protein subunit gamma 4 [Source:HGNC Symbol;Acc:HGNC:4407] | gtaacgtcgggttagttttcg | gaaaacctccgaccaaaacg | TCGA &GEO analysis |
| US-CG405 | ENSG00000183654 | MARCHF11 | 5:16067139-16180762:-1 | protein_coding | membrane associated ring-CH-type finger 11 [Source:HGNC Symbol;Acc:HGNC:33609] | cggttatagtttcgggtttcg | gcgaaacgaataccctaaacg | TCGA &GEO analysis |
| US-CG406 | ENSG00000183654 | MARCHF11 | 5:16067139-16180762:-1 | protein_coding | membrane associated ring-CH-type finger 11 [Source:HGNC Symbol;Acc:HGNC:33609] | ttagttcgttcgttcggagtcg | cgcgacgaaaataacgaaacg | TCGA &GEO analysis |
| US-CG407 | ENSG00000176635 | HORMAD2 | 22:30080174-30177075:1 | protein_coding | HORMA domain containing 2 [Source:HGNC Symbol;Acc:HGNC:28383] | tcgggttagtgtgtagttcg | gtccacgactaacctaaacg | TCGA &GEO analysis |
| US-CG408 | ENSG00000180053 | NKX2-6 | 8:23702451-23706598:-1 | protein_coding | NK2 homeobox 6 [Source:HGNC Symbol;Acc:HGNC:32940] | cgttattcggttttggattcg | ccctaaacttcccgtcttatcg | TCGA &GEO analysis |
| US-CG409 | ENSG00000189134 | NKAPL | 6:28259320-28260958:1 | protein_coding | NFKB activating protein like [Source:HGNC Symbol;Acc:HGNC:21584] | ttgataggaagcggaagtcg | ataaaccgcgcctcaacg | TCGA &GEO analysis |
| US-CG410 | ENSG00000274520 | Six3os1 | 2:44932320-44932406:1 | misc_RNA |  | cgtttataggttttagaggttagcg | cttctaatcgcgcgtaaacacg | TCGA &GEO analysis |
| US-CG411 | ENSG00000143869 | GDF7 | 2:20666664-20679245:1 | protein_coding | growth differentiation factor 7 [Source:HGNC Symbol;Acc:HGNC:4222] | cgggattcgcgttattatcg | gactaacgaaaaccaactaccg | TCGA &GEO analysis |
| US-CG412 | ENSG00000152977 | ZIC1 | 3:147393422-147510293:1 | protein_coding | Zic family member 1 [Source:HGNC Symbol;Acc:HGNC:12872] | cggtaataaagatcgcgttcg | cccttatcacaattattaaaattccg | TCGA &GEO analysis |
| US-CG413 | ENSG00000234377 | RNF219-AS1 | 13:77919689-78617328:1 | antisense | RNF219 antisense RNA 1 [Source:HGNC Symbol;Acc:HGNC:42700] | cggtttaaggtcgttgatttcg | cttccaactacccgaaatcg | TCGA &GEO analysis |
| US-CG414 | ENSG00000179059 | ZFP42 | 4:187995771-188005050:1 | protein_coding | ZFP42 zinc finger protein [Source:HGNC Symbol;Acc:HGNC:30949] | ggtggttgtagtttgattagatcg | aataccacctcctcccaaacg | TCGA &GEO analysis |
| US-CG415 | ENSG00000164418 | GRIK2 | 6:101181257-102070083:1 | protein_coding | glutamate ionotropic receptor kainate type subunit 2 [Source:HGNC Symbol;Acc:HGNC:4580] | gttaatgggtttgggaagcg | aacgttcgactcctaaattcg | TCGA &GEO analysis |
| US-CG416 | ENSG00000234602 | MCIDAS | 5:55219614-55227315:-1 | protein_coding | multiciliate differentiation and DNA synthesis associated cell cycle protein [Source:HGNC Symbol;Acc:HGNC:40050] | gaaataggaggagatcgtttcg | cgtactacaattcccacacg | TCGA &GEO analysis |
| US-CG417 | ENSG00000119042 | SATB2 | 2:199269500-199471266:-1 | protein_coding | SATB homeobox 2 [Source:HGNC Symbol;Acc:HGNC:21637] | ttggaggtgagtagttagcg | caataactcacctcgactcg | TCGA &GEO analysis |
| US-CG418 | ENSG00000283193 | MIR1243 | 4:113106863-113106955:1 | miRNA | microRNA 1243 [Source:HGNC Symbol;Acc:HGNC:35304] | gcggattgggatagtatagtcg | gaaaccgactcgaacttacg | TCGA &GEO analysis |
| US-CG419 | ENSG00000255150 | EID3 | 12:104303739-104305205:1 | protein_coding | EP300 interacting inhibitor of differentiation 3 [Source:HGNC Symbol;Acc:HGNC:32961] | gcgttttcggttttaattgtcg | ctactcctctcctttctcttcg | TCGA &GEO analysis |
| US-CG420 | ENSG00000187391 | MAGI2 | 7:78017057-79453574:-1 | protein_coding | membrane associated guanylate kinase, WW and PDZ domain containing 2 [Source:HGNC Symbol;Acc:HGNC:18957] | gttaggacgttcgtagagttcg | gcgccaaactcctatacg | TCGA &GEO analysis |
| US-CG421 | ENSG00000130675 | MNX1 | 7:156994051-157010651:-1 | protein_coding | motor neuron and pancreas homeobox 1 [Source:HGNC Symbol;Acc:HGNC:4979] | aagagcgatattaaaggaagacg | aattccgcgacgactaaacg | TCGA &GEO analysis |
| US-CG422 | ENSG00000107807 | TLX1 | 10:101130505-101137789:1 | protein_coding | T cell leukemia homeobox 1 [Source:HGNC Symbol;Acc:HGNC:5056] | atttgcgagttggagaagcg | actcacctccactttatccg | TCGA &GEO analysis |
| US-CG423 | ENSG00000267343 | ZNF833P | 19:11680944-11686259:1 | transcribed_unprocessed_pseudogene | zinc finger protein 833, pseudogene [Source:HGNC Symbol;Acc:HGNC:33819] | tcgtattggtagtaggagtcg | gccacgatctattcctaccg | TCGA &GEO analysis |
| US-CG424 | ENSG00000165462 | PHOX2A | 11:72239077-72245664:-1 | protein_coding | paired like homeobox 2A [Source:HGNC Symbol;Acc:HGNC:691] | ttcgattcggtttggttcg | cgccgaactaactacaaacg | TCGA &GEO analysis |
| US-CG425 | ENSG00000112319 | EYA4 | 6:133240598-133532120:1 | protein_coding | EYA transcriptional coactivator and phosphatase 4 [Source:HGNC Symbol;Acc:HGNC:3522] | ggcggttttacgagttcg | acgcgaaaaccgaaaacg | TCGA &GEO analysis |
| US-CG426 | ENSG00000166573 | GALR1 | 18:77250549-77277896:1 | protein_coding | galanin receptor 1 [Source:HGNC Symbol;Acc:HGNC:4132] | agtcgtatagtgtattgttgcg | cttaccgctccaaatcttcg | TCGA &GEO analysis |
| US-CG427 | ENSG00000245870 | LINC00682 | 4:41872747-41882955:-1 | lincRNA | long intergenic non-protein coding RNA 682 [Source:HGNC Symbol;Acc:HGNC:44466] | gggttgagcgatagatttacg | cgaactcgaatccaaaaccg | TCGA &GEO analysis |
| US-CG428 | ENSG00000149084 | HSD17B12 | 11:43680558-43856617:1 | protein_coding | hydroxysteroid 17-beta dehydrogenase 12 [Source:HGNC Symbol;Acc:HGNC:18646] | ggtttggagaaatggagatacg | tatctccgaaccctaaaaccg | TCGA &GEO analysis |
| US-CG429 | ENSG00000237988 | OR2I1P | 6:29550407-29557721:1 | protein_coding | olfactory receptor family 2 subfamily I member 1 pseudogene [Source:HGNC Symbol;Acc:HGNC:8258] | ggcgacggagatattatcgag | ccgcatacaacaaacaactcg | TCGA &GEO analysis |
| US-CG430 | ENSG00000186766 | FOXI2 | 10:127737235-127741186:1 | protein_coding | forkhead box I2 [Source:HGNC Symbol;Acc:HGNC:32448] | cgtgaagttagacgaggttacg | ccttcctcctttatacgatccg | TCGA &GEO analysis |
| US-CG431 | ENSG00000101438 | SLC32A1 | 20:38724462-38729372:1 | protein_coding | solute carrier family 32 member 1 [Source:HGNC Symbol;Acc:HGNC:11018] | cgtagtttagttcgcgtttcg | cataaccctcgcaactaacg | TCGA &GEO analysis |
| US-CG432 | ENSG00000274618 | HIST1H4F | 6:26240426-26240737:1 | protein_coding | histone cluster 1 H4 family member f [Source:HGNC Symbol;Acc:HGNC:4783] | gcgttatcgtaaagtgttgcg | cctcataaataaaacccgaaatacg | TCGA &GEO analysis |
| US-CG433 | ENSG00000078399 | HOXA9 | 7:27162435-27175180:-1 | protein_coding | homeobox A9 [Source:HGNC Symbol;Acc:HGNC:5109] | agttacggcgaggtaaacg | aaacaaccaaatcgcattatcg | TCGA &GEO analysis |
| US-CG434 | ENSG00000169594 | BNC1 | 15:83255903-83284714:-1 | protein_coding | basonuclin 1 [Source:HGNC Symbol;Acc:HGNC:1081] | ttcgggagaggtaaatatcg | cgcaactaaaacgaaaccg | TCGA &GEO analysis |
| US-CG435 | ENSG00000104899 | AMH | 19:2249309-2252073:1 | protein_coding | anti-Mullerian hormone [Source:HGNC Symbol;Acc:HGNC:464] | tgcggttggttttagttcgat | ccgcgtaaacgataaacacg | TCGA &GEO analysis |

**Supplementary Table S2. MSP result of 204 primer pairs for 176 candidate genes in 5 cell lines**

| ***ID*** | ***Gene*** | ***TM*** | ***Human Genome DNA*** | ***Universal Methylated DNA*** | ***SV-HUC-1*** | ***5637*** | ***SW780*** | ***T24*** | ***TCCSUP*** | ***SELECTED*** |
| --- | --- | --- | --- | --- | --- | --- | --- | --- | --- | --- |
| US-CG00 | ACTB | 55 | 5 | 5 | 5 | 5 | 5 | 5 | 5 | ***YES*** |
| US-CG01 | ABCC13 | 55 | 4 | 4 | 4 | 4 | 4 | 4 | 0 | ***YES*** |
| US-CG02 | ABCC6 | 55 | 2 | 4 | 1 | 3 | 1 | 2 | 1 | ***No*** |
| US-CG03 | ABCC8 | 55 | 0 | 2 | 0 | 1 | 0 | 1 | 1 | ***No*** |
| US-CG04 | ALX4 | 55 | 0 | 4 | 1 | 4 | 2 | 3 | 3 | ***No*** |
| US-CG05 | APC | 55 | 0 | 3 | 0 | 0 | 0 | 0 | 0 | ***No*** |
| US-CG06 | BCAR3 | 55 | 0 | 5 | 0 | 0 | 0 | 0 | 0 | ***No*** |
| US-CG07 | BCL2 | 55 | 0 | 3 | 0 | 2 | 2 | 1 | 0 | ***No*** |
| US-CG08 | GDF10 | 55 | 0 | 4 | 0 | 0 | 0 | 1 | 1 | ***No*** |
| US-CG09 | BNIP3 | 55 | 0 | 3 | 0 | 0 | 0 | 0 | 2 | ***No*** |
| US-CG10 | BRCA1 | 55 | 0 | 4 | 0 | 0 | 0 | 0 | 0 | ***No*** |
| US-CG100 | PRRT1 | 60 | 1 | 5 | 5 | 4 | 4 | 4 | 5 | ***YES*** |
| US-CG101 | PTPRF | 60 | 0 | 5 | 4 | 4 | 3 | 3 | 3 | ***YES*** |
| US-CG102 | REC8 | 60 | 1 | 5 | 5 | 5 | 5 | 5 | 5 | ***YES*** |
| US-CG103 | RFX1 | 60 | 2 | 3 | 3 | 3 | 3 | 3 | 3 | ***No*** |
| US-CG104 | RNF39 | 60 | 4 | 4 | 4 | 4 | 4 | 4 | 4 | ***No*** |
| US-CG105 | RNF39 | 60 | 5 | 5 | 5 | 5 | 4 | 4 | 5 | ***No*** |
| US-CG106 | RSPH9 | 60 | 2 | 2 | 2 | 2 | 2 | 2 | 2 | ***No*** |
| US-CG107 | SIM2 | 60 | 1 | 5 | 5 | 5 | 5 | 5 | 5 | ***YES*** |
| US-CG108 | SLC38A10 | 60 | 0 | 4 | 4 | 4 | 2 | 3 | 3 | ***No*** |
| US-CG109 | SOX1 | 60 | 1 | 4 | 0 | 3 | 2 | 3 | 1 | ***YES*** |
| US-CG11 | BRCA2 | 55 | 1 | 4 | 2 | 2 | 2 | 1 | 2 | ***No*** |
| US-CG110 | TACC2 | 60 | 0 | 5 | 5 | 5 | 5 | 5 | 5 | ***No*** |
| US-CG111 | TAF7 | 60 | 5 | 5 | 5 | 5 | 5 | 5 | 5 | ***No*** |
| US-CG111-2 | TAF7 | 60 | 5 | 5 | 5 | 4 | 4 | 5 | 5 | ***No*** |
| US-CG112 | TFAP2E | 60 | 0 | 4 | 4 | 3 | 2 | 3 | 3 | ***No*** |
| US-CG113 | ZAR1 | 60 | 1 | 5 | 3 | 4 | 3 | 3 | 3 | ***No*** |
| US-CG114 | ZNF154 | 60 | 0 | 0 | 0 | 0 | 0 | 0 | 0 | ***YES*** |
| US-CG115 | CYP26C1 | 60 | 1 | 3 | 1 | 2 | 1 | 1 | 2 | ***No*** |
| US-CG116 | CAPZB | 60 | 5 | 5 | 5 | 5 | 5 | 5 | 5 | ***No*** |
| US-CG116-2 | CAPZB | 60 | 5 | 5 | 5 | 5 | 5 | 5 | 5 | ***No*** |
| US-CG117 | CYTH1 | 65 | 5 | 5 | 5 | 5 | 5 | 5 | 5 | ***No*** |
| US-CG117-2 | CYTH1 | 60 | 5 | 5 | 5 | 5 | 5 | 5 | 5 | ***No*** |
| US-CG118 | DNM2 | 60 | 5 | 5 | 5 | 5 | 4 | 4 | 5 | ***No*** |
| US-CG118-2 | DNM2 | 63 | 5 | 5 | 5 | 5 | 4 | 4 | 5 | ***No*** |
| US-CG119 | H2AFY | 62 | 5 | 5 | 5 | 5 | 5 | 5 | 5 | ***No*** |
| US-CG12 | CBR1 | 55 | 0 | 3 | 0 | 0 | 0 | 0 | 0 | ***No*** |
| US-CG120 | SDCCAG8 | 62 | 0 | 4 | 4 | 4 | 4 | 4 | 4 | ***YES*** |
| US-CG120-2 | SDCCAG8 | 60 | 0 | 5 | 5 | 5 | 5 | 4 | 5 | ***YES*** |
| US-CG121 | TFAP2A | 58 | 1 | 4 | 4 | 3 | 3 | 3 | 3 | ***YES*** |
| US-CG121-2 | TFAP2A | 55 | 1 | 4 | 4 | 3 | 3 | 3 | 3 | ***YES*** |
| US-CG122 | VAV2 | 60 | 5 | 5 | 5 | 5 | 4 | 2 | 5 | ***No*** |
| US-CG122-2 | VAV2 | 60 | 4 | 4 | 4 | 4 | 3 | 2 | 4 | ***No*** |
| US-CG123 | ALX4 | 65 | 0 | 4 | 0 | 0 | 0 | 1 | 2 | ***No*** |
| US-CG124 | APC | 65 | 0 | 5 | 0 | 0 | 0 | 0 | 0 | ***No*** |
| US-CG125 | GDF10 | 65 | 0 | 3 | 0 | 0 | 0 | 3 | 0 | ***No*** |
| US-CG126 | BRCA1 | 60 | 3 | 3 | 3 | 3 | 1 | 3 | 3 | ***No*** |
| US-CG127 | BRCA2 | 60 | 0 | 1 | 0 | 0 | 0 | 0 | 0 | ***No*** |
| US-CG128 | CCNA1 | 60 | 0 | 4 | 0 | 4 | 0 | 0 | 2 | ***No*** |
| US-CG129 | DAPK1 | 60 | 0 | 3 | 0 | 0 | 0 | 2 | 0 | ***No*** |
| US-CG13 | CBR3 | 55 | 0 | 5 | 0 | 0 | 0 | 0 | 0 | ***No*** |
| US-CG130 | EDNRB | 60 | 2 | 3 | 1 | 1 | 2 | 2 | 3 | ***No*** |
| US-CG131 | GSTP1 | 55 | 0 | 2 | 0 | 0 | 0 | 0 | 0 | ***No*** |
| US-CG133 | MEIS1 | 60 | 1 | 5 | 0 | 0 | 0 | 0 | 0 | ***No*** |
| US-CG134 | MGMT | 65 | 0 | 4 | 0 | 1 | 1 | 2 | 0 | ***No*** |
| US-CG135 | OTX1 | 60 | 0 | 5 | 5 | 2 | 4 | 4 | 4 | ***YES*** |
| US-CG136 | PCDH10 | 55 | 2 | 5 | 0 | 0 | 4 | 3 | 0 | ***No*** |
| US-CG137 | RASSF1 | 60 | 0 | 2 | 0 | 2 | 0 | 2 | 2 | ***No*** |
| US-CG138 | SFRP4 | 65 | 0 | 3 | 0 | 0 | 0 | 1 | 0 | ***YES*** |
| US-CG139 | OGG1 | 60 | 0 | 4 | 0 | 0 | 0 | 0 | 0 | ***No*** |
| US-CG14 | CCNA1 | 55 | 1 | 5 | 0 | 5 | 1 | 1 | 5 | ***No*** |
| US-CG140 | ABCB1 | 65 | 0 | 5 | 0 | 5 | 5 | 1 | 0 | ***No*** |
| US-CG142 | ADAM12 | 65 | 1 | 4 | 0 | 0 | 1 | 0 | 0 | ***No*** |
| US-CG143 | ADAMTS1 | 55 | 0 | 2 | 0 | 0 | 0 | 0 | 0 | ***No*** |
| US-CG144 | ADAMTS15 | 60 | 0 | 5 | 0 | 0 | 0 | 0 | 0 | ***No*** |
| US-CG145 | ADAMTS8 | 65 | 0 | 4 | 0 | 0 | 0 | 1 | 0 | ***No*** |
| US-CG147 | ALDH1A3 | 65 | 0 | 4 | 0 | 0 | 0 | 0 | 3 | ***No*** |
| US-CG148 | ALPL | 60 | 0 | 4 | 0 | 0 | 2 | 2 | 0 | ***No*** |
| US-CG15 | CDH1 | 55 | 1 | 5 | 0 | 0 | 0 | 5 | 1 | ***No*** |
| US-CG154 | BARHL2 | 60 | 0 | 5 | 0 | 5 | 4 | 4 | 4 | ***YES*** |
| US-CG155 | ZMYND10 | 55 | 0 | 3 | 3 | 0 | 0 | 3 | 1 | ***No*** |
| US-CG159 | BNC1 | 55 | 0 | 4 | 1 | 3 | 3 | 0 | 4 | ***YES*** |
| US-CG16 | CDH13 | 55 | 1 | 5 | 1 | 0 | 0 | 0 | 5 | ***YES*** |
| US-CG160 | BOLL | 60 | 2 | 2 | 2 | 2 | 2 | 2 | 2 | ***No*** |
| US-CG162 | BVES | 55 | 0 | 5 | 0 | 0 | 1 | 3 | 0 | ***No*** |
| US-CG166 | CD34 | **65** | 0 | 3 | 0 | 1 | 1 | 2 | 1 | ***No*** |
| US-CG17 | CDKN1C | 55 | 0 | 3 | 0 | 0 | 0 | 0 | 0 | ***No*** |
| US-CG170 | CDH13 | 55 | 0 | 5 | 0 | 0 | 0 | 0 | 5 | ***No*** |
| US-CG171 | CDX2 | 60 | 0 | 5 | 0 | 0 | 0 | 2 | 0 | ***No*** |
| US-CG177 | CNTNAP2 | 65 | 0 | 5 | 0 | 0 | 0 | 0 | 0 | ***No*** |
| US-CG18 | CFTR | 55 | 2 | 5 | 1 | 5 | 5 | 5 | 5 | ***YES*** |
| US-CG180 | CXCL12 | 55 | 0 | 1 | 0 | 0 | 0 | 0 | 0 | ***No*** |
| US-CG181 | CYP1B1 | 55 | 0 | 3 | 0 | 1 | 1 | 0 | 0 | ***No*** |
| US-CG19 | COX20 | 55 | 1 | 5 | 0 | 0 | 0 | 0 | 0 | ***No*** |
| US-CG190 | DMRTA2 | 60 | 0 | 5 | 1 | 4 | 3 | 3 | 3 | ***YES*** |
| US-CG191 | DUOX1 | 60 | 0 | 1 | 1 | 0 | 1 | 0 | 0 | ***No*** |
| US-CG20 | DAPK1 | 55 | 0 | 4 | 0 | 0 | 0 | 2 | 0 | ***No*** |
| US-CG203 | EYA4 | 65 | 0 | 5 | 0 | 5 | 0 | 3 | 3 | ***YES*** |
| US-CG206 | FGF12 | 60 | 0 | 5 | 0 | 0 | 0 | 5 | 1 | ***No*** |
| US-CG21 | DRG1 | 55 | 0 | 5 | 0 | 0 | 0 | 0 | 0 | ***No*** |
| US-CG213 | GATA4 | 65 | 1 | 5 | 4 | 3 | 2 | 3 | 2 | ***YES*** |
| US-CG214 | GATA5 | 65 | 0 | 4 | 0 | 1 | 1 | 3 | 2 | ***No*** |
| US-CG215 | GDNF | 60 | 0 | 2 | 0 | 0 | 1 | 1 | 0 | ***No*** |
| US-CG216 | GFRA1 | 60 | 0 | 4 | 0 | 2 | 0 | 4 | 3 | ***No*** |
| US-CG217 | GNAL | 60 | 5 | 5 | 5 | 4 | 2 | 4 | 1 | ***YES*** |
| US-CG219 | GPC5 | 65 | 2 | 3 | 3 | 2 | 1 | 2 | 2 | ***NO*** |
| US-CG22 | GREM1 | 55 | 0 | 3 | 0 | 3 | 2 | 2 | 3 | ***YES*** |
| US-CG225 | HOXA11 | 60 | 0 | 5 | 0 | 0 | 2 | 2 | 0 | ***No*** |
| US-CG23 | EDNRB | 55 | 3 | 4 | 2 | 4 | 3 | 3 | 3 | ***No*** |
| US-CG231 | HS3ST2 | 60 | 0 | 1 | 0 | 0 | 0 | 0 | 0 | ***No*** |
| US-CG233 | HTR1B | 55 | 1 | 5 | 1 | 1 | 1 | 4 | 4 | ***No*** |
| US-CG237 | IL20RA | 60 | 0 | 5 | 0 | 0 | 0 | 0 | 1 | ***No*** |
| US-CG239 | IRX2 | 60 | 1 | 4 | 0 | 3 | 3 | 0 | 4 | ***YES*** |
| US-CG24 | FADD | 55 | 0 | 2 | 0 | 0 | 0 | 0 | 0 | ***No*** |
| US-CG240 | KCNH5 | 60 | 0 | 4 | 0 | 0 | 0 | 3 | 2 | ***No*** |
| US-CG242 | KIF1A | 65 | 0 | 2 | 0 | 0 | 0 | 1 | 0 | ***No*** |
| US-CG246 | LHX4 | 60 | 0 | 4 | 0 | 0 | 0 | 0 | 0 | ***No*** |
| US-CG248 | MAL | 60 | 0 | 4 | 4 | 2 | 2 | 3 | 3 | ***YES*** |
| US-CG249 | MAP1LC3A | 65 | 0 | 5 | 2 | 5 | 3 | 5 | 5 | ***YES*** |
| US-CG25 | GALC | 55 | 0 | 4 | 0 | 0 | 0 | 3 | 2 | ***YES*** |
| US-CG251 | MDFI | 65 | 0 | 3 | 3 | 2 | 3 | 3 | 3 | ***YES*** |
| US-CG256 | MSX1 | 60 | 0 | 4 | 0 | 0 | 0 | 0 | 1 | ***No*** |
| US-CG26 | GSTP1 | 55 | 0 | 3 | 0 | 0 | 0 | 0 | 1 | ***No*** |
| US-CG264 | NPY | 55 | 2 | 5 | 2 | 4 | 2 | 2 | 4 | ***No*** |
| US-CG265 | NR2E1 | 60 | 0 | 3 | 0 | 2 | 2 | 2 | 0 | ***No*** |
| US-CG266 | NRCAM | 55 | 1 | 5 | 0 | 0 | 0 | 3 | 0 | ***No*** |
| US-CG27 | FOXA2 | 55 | 1 | 4 | 0 | 3 | 0 | 4 | 0 | ***YES*** |
| US-CG270 | OPCML | 55 | 3 | 5 | 5 | 1 | 0 | 5 | 2 | ***No*** |
| US-CG271 | OSR1 | 60 | 0 | 3 | 2 | 3 | 2 | 2 | 2 | ***No*** |
| US-CG273 | CDKN2A | 55 | 0 | 3 | 0 | 0 | 0 | 0 | 1 | ***No*** |
| US-CG275 | PAX5 | 65 | 2 | 4 | 3 | 3 | 3 | 3 | 3 | ***YES*** |
| US-CG277 | PAX8 | 55 | 0 | 2 | 1 | 2 | 2 | 0 | 2 | ***No*** |
| US-CG279 | PDX1 | 55 | 0 | 2 | 0 | 0 | 0 | 2 | 0 | ***No*** |
| US-CG28 | TMEFF2 | 55 | 0 | 3 | 0 | 0 | 4 | 0 | 4 | ***No*** |
| US-CG281 | PHOX2A | 60 | 1 | 4 | 3 | 4 | 0 | 4 | 4 | ***YES*** |
| US-CG282 | PITX2 | 55 | 0 | 4 | 0 | 0 | 3 | 3 | 0 | ***No*** |
| US-CG283 | PRDM14 | 60 | 0 | 3 | 1 | 2 | 1 | 2 | 0 | ***No*** |
| US-CG286 | PTGER2 | 65 | 0 | 4 | 2 | 1 | 3 | 3 | 3 | ***No*** |
| US-CG29 | TERT | 55 | 0 | 2 | 0 | 2 | 1 | 2 | 0 | ***No*** |
| US-CG30 | ICAM1 | 55 | 0 | 4 | 0 | 0 | 0 | 0 | 0 | ***No*** |
| US-CG300 | RUNX1 | 65 | 5 | 5 | 5 | 5 | 5 | 5 | 5 | ***YES*** |
| US-CG306 | SHOX2 | 60 | 0 | 4 | 0 | 2 | 0 | 3 | 3 | ***NO*** |
| US-CG308 | SIX1 | 65 | 1 | 5 | 0 | 0 | 0 | 0 | 0 | ***NO*** |
| US-CG31 | ITGA4 | 55 | 0 | 4 | 0 | 2 | 3 | 3 | 2 | ***YES*** |
| US-CG310 | SIX3 | 62 | 0 | 5 | 5 | 5 | 3 | 4 | 4 | ***YES*** |
| US-CG316 | SORCS3 | 65 | 0 | 3 | 0 | 3 | 1 | 3 | 2 | ***NO*** |
| US-CG318 | TAC1 | 65 | 0 | 5 | 0 | 0 | 1 | 3 | 0 | ***NO*** |
| US-CG32 | LAMA3 | 55 | 0 | 2 | 0 | 0 | 0 | 0 | 0 | ***No*** |
| US-CG320 | TBX5 | 60 | 0 | 2 | 0 | 1 | 0 | 1 | 0 | ***NO*** |
| US-CG327 | ZIC4 | 60 | 0 | 3 | 0 | 2 | 1 | 2 | 1 | ***YES*** |
| US-CG33 | LITAF | 55 | 0 | 4 | 0 | 0 | 0 | 0 | 0 | ***No*** |
| US-CG34 | MAGEA1 | 55 | 5 | 5 | 5 | 4 | 5 | 5 | 5 | ***No*** |
| US-CG35 | ABCB1 | 55 | 0 | 1 | 0 | 1 | 1 | 0 | 0 | ***No*** |
| US-CG352 | OTX1 | 55 | 1 | 3 | 1 | 3 | 3 | 3 | 2 | ***YES*** |
| US-CG36 | MGMT | 55 | 0 | 5 | 0 | 1 | 0 | 0 | 0 | ***No*** |
| US-CG37 | APBA2 | 55 | 3 | 3 | 3 | 3 | 3 | 2 | 3 | ***YES*** |
| US-CG38 | APBA1 | 55 | 1 | 4 | 3 | 1 | 0 | 3 | 1 | ***No*** |
| US-CG39 | MT1A | 55 | 1 | 4 | 4 | 1 | 4 | 4 | 4 | ***YES*** |
| US-CG40 | MTSS1 | 55 | 0 | 3 | 1 | 0 | 0 | 0 | 0 | ***No*** |
| US-CG41 | MYOD1 | 55 | 0 | 4 | 0 | 4 | 1 | 2 | 1 | ***YES*** |
| US-CG42 | OCLN | 55 | 0 | 4 | 0 | 0 | 0 | 0 | 0 | ***No*** |
| US-CG43 | CDKN2A | 55 | 5 | 4 | 4 | 1 | 3 | 3 | 0 | ***No*** |
| US-CG44 | CDKN2A | 55 | 5 | 4 | 4 | 1 | 3 | 3 | 0 | ***No*** |
| US-CG45 | DISP3 | 55 | 1 | 3 | 0 | 3 | 1 | 3 | 2 | ***No*** |
| US-CG46 | RASSF1 | 55 | 0 | 3 | 0 | 3 | 0 | 3 | 3 | ***YES*** |
| US-CG47 | RPRM | 55 | 0 | 3 | 0 | 0 | 3 | 0 | 0 | ***No*** |
| US-CG48 | RUNX3 | 55 | 0 | 3 | 0 | 0 | 3 | 0 | 0 | ***No*** |
| US-CG49 | SALL3 | 55 | 1 | 3 | 0 | 3 | 3 | 3 | 4 | ***YES*** |
| US-CG50 | SERPINB5 | 55 | 4 | 4 | 0 | 0 | 0 | 0 | 0 | ***YES*** |
| US-CG51 | SLC29A1 | 55 | 0 | 2 | 3 | 0 | 3 | 0 | 0 | ***No*** |
| US-CG52 | STAT1 | 55 | 0 | 4 | 0 | 0 | 0 | 0 | 0 | ***No*** |
| US-CG53 | TIMP3 | 55 | 0 | 4 | 0 | 0 | 0 | 0 | 0 | ***No*** |
| US-CG54 | PYCARD | 55 | 0 | 4 | 2 | 2 | 4 | 0 | 4 | ***No*** |
| US-CG55 | TNFRSF10A | 55 | 0 | 3 | 0 | 0 | 0 | 0 | 0 | ***No*** |
| US-CG56 | TNFRSF10C | 55 | 1 | 2 | 3 | 1 | 1 | 3 | 1 | ***No*** |
| US-CG57 | TNFRSF10D | 55 | 1 | 4 | 1 | 1 | 0 | 1 | 3 | ***No*** |
| US-CG58 | TNFRSF21 | 55 | 0 | 2 | 0 | 0 | 0 | 0 | 0 | ***No*** |
| US-CG59 | WWOX | 55 | 1 | 3 | 0 | 0 | 0 | 0 | 0 | ***No*** |
| US-CG61 | CRYBG1 | 55 | 1 | 4 | 1 | 1 | 1 | 1 | 1 | ***No*** |
| US-CG62 | CALCA | 55 | 0 | 4 | 1 | 1 | 2 | 3 | 0 | ***YES*** |
| US-CG63 | CCND2 | 55 | 0 | 3 | 0 | 2 | 1 | 1 | 0 | ***YES*** |
| US-CG65 | DCC | 55 | 0 | 0 | 0 | 0 | 0 | 0 | 0 | ***No*** |
| US-CG66 | DKK3 | 55 | 0 | 3 | 1 | 3 | 3 | 0 | 3 | ***YES*** |
| US-CG67 | ESR1 | 55 | 1 | 4 | 0 | 0 | 0 | 4 | 0 | ***No*** |
| US-CG68 | FHIT | 55 | 1 | 4 | 0 | 0 | 0 | 4 | 0 | ***No*** |
| US-CG69 | HIC1 | 55 | 0 | 4 | 0 | 0 | 1 | 0 | 0 | ***No*** |
| US-CG70 | HOXA9 | 55 | 1 | 4 | 4 | 0 | 4 | 4 | 0 | ***YES*** |
| US-CG71 | MT1G | 55 | 0 | 3 | 3 | 2 | 2 | 3 | 0 | ***YES*** |
| US-CG72 | PCDH10 | 55 | 3 | 4 | 3 | 3 | 3 | 3 | 0 | ***YES*** |
| US-CG73 | PENK | 55 | 1 | 3 | 0 | 3 | 1 | 3 | 1 | ***YES*** |
| US-CG74 | SFRP1 | 55 | 3 | 4 | 3 | 4 | 4 | 4 | 4 | ***No*** |
| US-CG75 | sFRP2 | 55 | 1 | 4 | 1 | 4 | 3 | 3 | 2 | ***YES*** |
| US-CG77 | TCF21 | 55 | 3 | 4 | 3 | 3 | 3 | 3 | 3 | ***No*** |
| US-CG78 | TMEFF2 | 55 | 1 | 4 | 0 | 1 | 3 | 0 | 3 | ***No*** |
| US-CG79 | VIM | 55 | 0 | 4 | 0 | 0 | 2 | 3 | 0 | ***No*** |
| US-CG80 | WIF1 | 55 | 0 | 2 | 0 | 0 | 1 | 2 | 0 | ***No*** |
| US-CG81 | AGRN | 60 | 0 | 5 | 5 | 5 | 4 | 5 | 5 | ***YES*** |
| US-CG82 | AMZ1 | 60 | 5 | 5 | 5 | 5 | 4 | 4 | 4 | ***No*** |
| US-CG83 | AMZ1 | 60 | 2 | 4 | 4 | 2 | 2 | 0 | 2 | ***No*** |
| US-CG84 | CHAD | 60 | 1 | 4 | 4 | 4 | 4 | 4 | 3 | ***YES*** |
| US-CG85 | CHAD | 60 | 2 | 5 | 5 | 5 | 5 | 5 | 5 | ***YES*** |
| US-CG86 | CIT | 60 | 5 | 5 | 5 | 5 | 5 | 5 | 5 | ***No*** |
| US-CG87 | CPT1B | 60 | 4 | 4 | 4 | 4 | 3 | 4 | 4 | ***No*** |
| US-CG88 | CYP26C1 | 60 | 0 | 0 | 0 | 0 | 0 | 0 | 0 | ***No*** |
| US-CG89 | DDAH2 | 60 | 2 | 2 | 1 | 1 | 1 | 1 | 1 | ***No*** |
| US-CG90 | DRD4 | 60 | 1 | 5 | 5 | 5 | 4 | 4 | 4 | ***YES*** |
| US-CG91 | HSPB6 | 60 | 5 | 5 | 5 | 5 | 5 | 4 | 5 | ***No*** |
| US-CG92 | KCNAB3 | 60 | 5 | 5 | 5 | 5 | 5 | 5 | 5 | ***No*** |
| US-CG93 | LY6G5C | 60 | 2 | 3 | 2 | 2 | 2 | 2 | 3 | ***No*** |
| US-CG94 | MEIS1 | 60 | 1 | 5 | 5 | 4 | 4 | 4 | 4 | ***YES*** |
| US-CG95 | NKX6-2 | 60 | 1 | 5 | 1 | 4 | 5 | 4 | 5 | ***YES*** |
| US-CG96 | OTX1 | 60 | 1 | 5 | 5 | 5 | 5 | 5 | 5 | ***YES*** |
| US-CG97 | OTX1 | 60 | 1 | 5 | 5 | 5 | 4 | 4 | 4 | ***YES*** |
| US-CG98 | PITX1 | 60 | 0 | 2 | 2 | 1 | 1 | 1 | 1 | ***No*** |
| US-CG99 | PPP1R13L | 60 | 1 | 5 | 5 | 5 | 5 | 5 | 5 | ***No*** |

**Supplementary Table S3. MSP result of 85 primer pairs for 69 candidate genes by SYBR green qMSP in 5 cell lines**

| ***ID*** | ***Gene*** | ***TM*** | ***NTC*** | ***Human Genome DNA*** | ***Universal Methylated DNA*** | ***SV-HUC-1*** | ***5637*** | ***SW780*** | ***T24*** | ***TCCSUP*** | ***SELECTED*** |
| --- | --- | --- | --- | --- | --- | --- | --- | --- | --- | --- | --- |
| US-CG00 | ACTB | 55 | 42.00 | 24.83 | 25.26 | 25.30 | 26.67 | 27.18 | 28.97 | 26.05 | ***YES*** |
| US-CG01 | ABCC13 | 60 | 42.00 | 28.88 | 28.18 | 28.30 | 28.84 | 29.69 | 29.99 | 40.00 | ***No*** |
| US-CG100 | PRRT1 | 64 | 42.00 | 34.14 | 27.99 | 29.63 | 30.50 | 30.48 | 30.95 | 28.95 | ***No*** |
| US-CG101 | PTPRF | 60 | 34.98 | 30.75 | 30.14 | 30.25 | 31.84 | 33.73 | 32.69 | 31.89 | ***No*** |
| US-CG102 | REC8 | 60 | 42.00 | 35.50 | 28.99 | 28.73 | 29.80 | 30.23 | 31.36 | 29.98 | ***YES*** |
| US-CG107 | SIM2 | 60 | 42.00 | 30.48 | 26.92 | 27.18 | 27.57 | 29.00 | 29.35 | 28.28 | ***YES*** |
| US-CG109 | SOX1 | 60 | 42.00 | 32.64 | 26.91 | 34.25 | 28.30 | 30.92 | 29.18 | 31.19 | ***YES*** |
| US-CG114 | ZNF154 | 60 | 42.00 | 40.00 | 29.81 | 31.80 | 31.46 | 32.31 | 33.19 | 32.08 | ***YES*** |
| US-CG120 | SDCCAG8 | 62 | 42.00 | 35.83 | 27.79 | 27.32 | 28.03 | 29.05 | 29.18 | 27.36 | ***No*** |
| US-CG120-2 | SDCCAG8 | 60 | 42.00 | 35.98 | 28.21 | 28.66 | 28.62 | 30.19 | 29.72 | 29.16 | ***No*** |
| US-CG121 | TFAP2A | 58 | 29.82 | 26.21 | 25.73 | 25.65 | 27.61 | 28.16 | 27.85 | 27.34 | ***No*** |
| US-CG121-2 | TFAP2A | 55 | 31.99 | 30.79 | 26.75 | 26.15 | 28.63 | 29.18 | 29.06 | 28.54 | ***No*** |
| US-CG135 | OTX1 | 60 | 42.00 | 40.00 | 28.46 | 29.47 | 34.47 | 31.71 | 30.68 | 30.97 | ***YES*** |
| US-CG138 | sFRP4 | 55 | 42.00 | 29.87 | 27.00 | 35.90 | 29.60 | 40.00 | 29.68 | 36.50 | ***No*** |
| US-CG154 | BARHL2 | 60 | 42.00 | 42.00 | 30.68 | 38.21 | 31.29 | 32.30 | 32.08 | 31.71 | ***YES*** |
| US-CG159 | BNC1 | 55 | 42.00 | 36.50 | 30.44 | 33.50 | 31.48 | 32.46 | 40.00 | 31.44 | ***No*** |
| US-CG16 | CDH13 | 60 | 36.09 | 34.20 | 27.79 | 26.98 | 40.00 | 40.00 | 35.87 | 40.00 | ***No*** |
| US-CG18 | CFTR | 60 | 42.00 | 40.00 | 38.01 | 40.00 | 40.00 | 40.00 | 40.00 | 40.00 | ***No*** |
| US-CG190 | DMRTA2 | 60 | 42.00 | 38.14 | 29.36 | 32.56 | 30.43 | 30.95 | 31.00 | 30.34 | ***YES*** |
| US-CG203 | EYA4 | 60 | 42.00 | 36.59 | 27.91 | 40.00 | 29.21 | 32.96 | 30.68 | 28.11 | ***No*** |
| US-CG213 | GATA4 | 65 | 42.00 | 35.82 | 28.79 | 29.14 | 31.03 | 32.08 | 30.94 | 32.68 | ***No*** |
| US-CG217 | GNAL | 60 | 42.00 | 28.79 | 29.27 | 29.71 | 31.85 | 32.95 | 32.00 | 34.29 | ***No*** |
| US-CG22 | GREM1 | 60 | 42.00 | 38.46 | 30.09 | 40.00 | 31.46 | 31.63 | 32.12 | 35.15 | ***No*** |
| US-CG239 | IRX2 | 60 | 42.00 | 32.62 | 28.99 | 40.00 | 31.06 | 31.19 | 42.00 | 29.03 | ***YES*** |
| US-CG248 | MAL | 60 | 42.00 | 34.92 | 28.99 | 29.02 | 31.22 | 30.94 | 31.05 | 30.65 | ***No*** |
| US-CG249 | MAP1LC3A | 60 | 40.00 | 39.06 | 28.65 | 31.08 | 29.53 | 30.68 | 29.82 | 29.99 | ***No*** |
| US-CG25 | GALC | 60 | 42.00 | 38.43 | 28.37 | 42.00 | 40.00 | 42.00 | 30.27 | 33.71 | ***No*** |
| US-CG251 | MDFI | 65 | 42.00 | 42.00 | 28.15 | 28.91 | 34.59 | 29.75 | 30.75 | 29.49 | ***No*** |
| US-CG27 | FOXA2 | 60 | 42.00 | 35.04 | 28.64 | 28.02 | 40.00 | 40.00 | 29.85 | 29.56 | ***No*** |
| US-CG275 | PAX5 | 65 | 42.00 | 36.68 | 27.73 | 30.83 | 29.15 | 31.53 | 30.08 | 30.30 | ***No*** |
| US-CG281 | PHOX2A | 60 | 37.77 | 38.34 | 33.55 | 33.65 | 33.31 | 36.00 | 34.10 | 33.45 | ***No*** |
| US-CG300 | RUNX1 | 65 | 42.00 | 27.17 | 27.84 | 28.19 | 28.58 | 29.89 | 30.66 | 29.14 | ***No*** |
| US-CG303 | SEPT9 | 60 | 42.00 | 42.00 | 30.27 | 29.65 | 31.09 | 33.11 | 32.28 | 32.62 | ***YES*** |
| US-CG31 | ITGA4 | 60 | 42.00 | 39.38 | 29.85 | 34.17 | 31.58 | 29.61 | 30.43 | 33.72 | ***No*** |
| US-CG310 | SIX3 | 62 | 42.00 | 35.27 | 27.47 | 28.35 | 28.32 | 29.85 | 29.51 | 29.63 | ***No*** |
| US-CG327 | ZIC4 | 60 | 42.00 | 38.82 | 32.66 | 40.00 | 33.83 | 34.78 | 34.63 | 36.09 | ***YES*** |
| US-CG332 | CDX2 | 60 | 42.00 | 42.00 | 27.24 | 28.19 | 34.13 | 30.20 | 29.59 | 42.00 | ***No*** |
| US-CG333 | CDX2 | 60 | 42.00 | 33.04 | 27.73 | 29.44 | 28.55 | 31.30 | 31.33 | 33.58 | ***No*** |
| US-CG334 | CDX2 | 60 | 42.00 | 40.00 | 30.61 | 38.65 | 40.00 | 39.58 | 37.18 | 42.00 | ***No*** |
| US-CG335 | CYP1B1 | 55 | 42.00 | 40.00 | 32.02 | 40.00 | 32.16 | 34.21 | 36.57 | 42.00 | ***No*** |
| US-CG336 | CYP1B1 | 60 | 42.00 | 37.00 | 28.67 | 31.53 | 29.83 | 32.14 | 30.12 | 38.39 | ***No*** |
| US-CG352 | OTX1 | 58 | 38.05 | 33.79 | 28.90 | 29.24 | 34.18 | 31.11 | 30.65 | 30.69 | ***No*** |
| US-CG353 | OTX1 | 58 | 38.64 | 34.89 | 29.09 | 29.60 | 30.79 | 31.57 | 31.13 | 30.60 | ***No*** |
| US-CG354 | OTX1 | 60 | 37.45 | 36.71 | 28.09 | 27.97 | 29.13 | 31.02 | 29.88 | 29.83 | ***No*** |
| US-CG355 | OTX1 | 60 | 37.45 | 36.71 | 28.09 | 27.97 | 29.13 | 31.02 | 29.88 | 29.83 | ***No*** |
| US-CG357 | ZNF154 | 60 | 42.00 | 32.20 | 27.09 | 28.17 | 28.19 | 29.49 | 29.51 | 28.58 | ***YES*** |
| US-CG358 | ZNF154 | 58 | 42.00 | 33.19 | 28.60 | 28.97 | 29.79 | 30.09 | 30.72 | 29.59 | ***YES*** |
| US-CG359 | TACC2 | 58 | 35.75 | 36.24 | 31.18 | 24.48 | 32.54 | 33.51 | 33.53 | 32.09 | ***No*** |
| US-CG362 | MEIS1 | 60 | 42.00 | 33.78 | 27.07 | 27.72 | 28.58 | 30.11 | 29.17 | 34.48 | ***YES*** |
| US-CG363 | CMTM2 | 60 | 42.00 | 40.00 | 31.64 | 31.51 | 33.25 | 36.29 | 33.94 | 32.91 | ***YES*** |
| US-CG365 | LY6G5C | 60 | 42.00 | 30.02 | 29.58 | 30.69 | 30.91 | 32.70 | 31.72 | 30.52 | ***YES*** |
| US-CG369 | PRRT1 | 58 | 36.25 | 34.65 | 28.71 | 29.58 | 29.82 | 30.74 | 31.53 | 29.54 | ***No*** |
| US-CG37 | APBA2 | 60 | 42.00 | 26.49 | 29.96 | 28.13 | 28.82 | 30.51 | 33.56 | 29.51 | ***No*** |
| US-CG371 | SIM2 | 58 | 40.00 | 34.09 | 30.68 | 32.26 | 34.30 | 35.05 | 35.90 | 34.53 | ***No*** |
| US-CG374 | DRD4 | 60 | 42.00 | 36.74 | 28.23 | 28.25 | 29.10 | 31.86 | 30.08 | 29.55 | ***YES*** |
| US-CG375 | TFAP2A | 60 | 42.00 | 35.58 | 28.31 | 27.24 | 29.78 | 31.24 | 30.10 | 30.57 | ***No*** |
| US-CG379 | PAX6 | 60 | 33.50 | 33.79 | 27.34 | 27.46 | 27.94 | 30.55 | 28.59 | 27.82 | ***No*** |
| US-CG380 | SPATA32 | 60 | 42.00 | 38.18 | 28.98 | 28.97 | 29.36 | 30.75 | 30.54 | 30.00 | ***YES*** |
| US-CG381 | KCNQ1DN | 60 | 40.00 | 37.70 | 31.75 | 32.60 | 32.59 | 34.81 | 33.19 | 32.69 | ***No*** |
| US-CG383 | ZNF814 | 60 | 42.00 | 32.78 | 26.54 | 26.77 | 28.06 | 27.98 | 28.50 | 27.32 | ***YES*** |
| US-CG384 | SEPT9 | 60 | 42.00 | 31.69 | 28.95 | 28.81 | 29.15 | 30.11 | 29.83 | 29.66 | ***YES*** |
| US-CG387 | ADAM32 | 60 | 42.00 | 34.93 | 29.21 | 29.22 | 30.64 | 31.51 | 31.54 | 30.88 | ***YES*** |
| US-CG39 | MT1A | 60 | 42.00 | 36.68 | 28.67 | 40.00 | 30.89 | 27.92 | 30.45 | 29.85 | ***No*** |
| US-CG392 | RUNX3 | 60 | 42.00 | 26.33 | 27.05 | 27.76 | 28.26 | 29.37 | 28.79 | 30.33 | ***YES*** |
| US-CG393 | PROCA1 | 60 | 42.00 | 42.00 | 34.98 | 36.09 | 36.51 | 36.18 | 36.92 | 36.73 | ***No*** |
| US-CG395 | HCG15 | 60 | 42.00 | 42.00 | 28.79 | 29.80 | 29.77 | 31.96 | 30.34 | 29.51 | ***YES*** |
| US-CG41 | MYOD1 | 65 | 42.00 | 42.00 | 38.85 | 35.13 | 29.90 | 28.02 | 40.00 | 28.96 | ***No*** |
| US-CG46 | RASSF1 | 60 | 42.00 | 42.00 | 33.15 | 39.50 | 40.00 | 35.61 | 34.64 | 37.39 | ***No*** |
| US-CG49 | SALL3 | 60 | 42.00 | 35.93 | 29.63 | 40.00 | 30.27 | 28.08 | 30.35 | 30.23 | ***No*** |
| US-CG50 | SERPINB5 | 65 | 42.00 | 31.95 | 33.29 | 26.79 | 23.91 | 24.66 | 42.00 | 25.73 | ***No*** |
| US-CG62 | CALCA | 60 | 42.00 | 39.46 | 28.67 | 34.75 | 35.65 | 33.15 | 31.10 | 42.00 | ***YES*** |
| US-CG63 | CCND2 | 60 | 42.00 | 38.70 | 28.12 | 42.00 | 31.46 | 34.58 | 35.54 | 42.00 | ***No*** |
| US-CG66 | DKK3 | 60 | 42.00 | 40.00 | 32.49 | 40.00 | 34.31 | 36.92 | 42.00 | 35.57 | ***No*** |
| US-CG70 | HOXA9 | 60 | 42.00 | 34.24 | 28.70 | 31.97 | 37.08 | 30.89 | 30.96 | 40.00 | ***No*** |
| US-CG71 | MT1G | 62 | 42.00 | 42.00 | 31.33 | 32.30 | 36.48 | 37.59 | 33.04 | 42.00 | ***No*** |
| US-CG72 | PCDH10 | 65 | 42.00 | 40.00 | 32.16 | 40.00 | 37.41 | 31.78 | 33.55 | 40.00 | ***No*** |
| US-CG73 | PENK | 62 | 42.00 | 36.71 | 29.90 | 40.00 | 31.36 | 40.00 | 31.67 | 40.00 | ***YES*** |
| US-CG75 | sFRP2 | 62 | 42.00 | 36.87 | 28.45 | 39.11 | 30.04 | 31.50 | 31.31 | 34.94 | ***No*** |
| US-CG81 | AGRN | 60 | 42.00 | 36.80 | 26.92 | 26.70 | 27.83 | 29.01 | 27.96 | 27.89 | ***No*** |
| US-CG84 | CHAD | 60 | 42.00 | 32.78 | 28.28 | 28.46 | 28.78 | 29.73 | 30.60 | 29.26 | ***YES*** |
| US-CG85 | CHAD | 62 | 35.12 | 30.65 | 26.96 | 28.21 | 28.33 | 29.23 | 30.32 | 29.59 | ***YES*** |
| US-CG90 | DRD4 | 65 | 42.00 | 42.00 | 32.14 | 31.86 | 33.05 | 34.46 | 34.24 | 32.93 | ***YES*** |
| US-CG94 | MEIS1 | 64 | 42.00 | 38.00 | 28.92 | 29.35 | 30.72 | 31.15 | 31.26 | 31.73 | ***YES*** |
| US-CG95 | NKX6-2 | 60 | 42.00 | 34.46 | 27.94 | 33.16 | 29.45 | 30.11 | 29.93 | 29.02 | ***YES*** |
| US-CG96 | OTX1 | 60 | 42.00 | 35.86 | 29.04 | 29.48 | 30.79 | 30.87 | 31.69 | 30.17 | ***YES*** |
| US-CG97 | OTX1 | 60 | 42.00 | 33.29 | 26.32 | 26.35 | 27.83 | 28.23 | 28.22 | 27.66 | ***YES*** |

**Supplementary Table S4. MSP result of 32 primer pairs for 23 candidate genes by SYBR green qMSP in 10 pairs of BC and normal tissues**

| ***ID*** | ***GENE*** | ***Cancer No*** | ***Contral No*** | ***Cut-off value*** | ***TP*** | ***FP*** | ***FN*** | ***TN*** | ***TPR(SEN)*** | ***TNR(SPE)*** | ***SELECTED*** |
| --- | --- | --- | --- | --- | --- | --- | --- | --- | --- | --- | --- |
| US-CG102 | REC8 | 10 | 10 | 33.7 | 5 | 1 | 5 | 9 | 50.0 | 90.0 | ***No*** |
| US-CG107 | SIM2 | 10 | 10 | 32.4 | 6 | 1 | 4 | 9 | 60.0 | 90.0 | ***No*** |
| US-CG109 | SOX1 | 10 | 10 | 32.9 | 3 | 1 | 7 | 9 | 30.0 | 90.0 | ***No*** |
| US-CG114 | ZNF154 | 10 | 10 | 34.8 | 5 | 1 | 5 | 9 | 50.0 | 90.0 | ***No*** |
| US-CG135 | OTX1 | 10 | 10 | 32.9 | 7 | 1 | 3 | 9 | 70.0 | 90.0 | ***No*** |
| US-CG154 | BARHL2 | 10 | 10 | 35.2 | 7 | 1 | 3 | 9 | 70.0 | 90.0 | ***No*** |
| US-CG190 | DMRTA2 | 10 | 10 | 37.2 | 9 | 1 | 1 | 9 | ***90.0*** | ***90.0*** | ***Yes*** |
| US-CG239 | IRX2 | 10 | 10 | 35.6 | 2 | 1 | 8 | 9 | 20.0 | 90.0 | ***No*** |
| US-CG303 | SEPT9 | 10 | 10 | 32.8 | 5 | 1 | 5 | 9 | 50.0 | 90.0 | ***No*** |
| US-CG327 | ZIC4 | 10 | 10 | 34.8 | 5 | 1 | 5 | 9 | 50.0 | 90.0 | ***No*** |
| US-CG357 | ZNF154 | 10 | 10 | 33.0 | 7 | 1 | 3 | 9 | 70.0 | 90.0 | ***No*** |
| US-CG358 | ZNF154 | 10 | 10 | 31.5 | 3 | 1 | 7 | 9 | 30.0 | 90.0 | ***No*** |
| US-CG362 | MEIS1 | 10 | 10 | 33.5 | 7 | 1 | 3 | 9 | 70.0 | 90.0 | ***No*** |
| US-CG363 | CMTM2 | 10 | 10 | 37.2 | 9 | 1 | 1 | 9 | ***90.0*** | ***90.0*** | ***Yes*** |
| US-CG365 | LY6G5C | 10 | 10 | 31.1 | 1 | 1 | 9 | 9 | 10.0 | 90.0 | ***No*** |
| US-CG373 | DRD4 | 10 | 10 | 35.5 | 9 | 1 | 1 | 9 | ***90.0*** | ***90.0*** | ***Yes*** |
| US-CG374 | DRD4 | 10 | 10 | 34.6 | 7 | 1 | 3 | 9 | 70.0 | 90.0 | ***No*** |
| US-CG380 | SPATA32 | 10 | 10 | 36.8 | 3 | 1 | 7 | 9 | 30.0 | 90.0 | ***No*** |
| US-CG383 | ZNF814 | 10 | 10 | 34.8 | 5 | 1 | 5 | 9 | 50.0 | 90.0 | ***No*** |
| US-CG384 | SEPT9 | 10 | 10 | 33.2 | 6 | 1 | 4 | 9 | 60.0 | 90.0 | ***No*** |
| US-CG387 | ADAM32 | 10 | 10 | 36.5 | 5 | 1 | 5 | 9 | 50.0 | 90.0 | ***No*** |
| US-CG392 | RUNX3 | 10 | 10 | 30.0 | 1 | 1 | 9 | 9 | 10.0 | 90.0 | ***No*** |
| US-CG395 | HCG15 | 10 | 10 | 33.5 | 5 | 1 | 5 | 9 | 50.0 | 90.0 | ***No*** |
| US-CG62 | CALCA | 10 | 10 | 37.0 | 2 | 1 | 8 | 9 | 20.0 | 90.0 | ***No*** |
| US-CG73 | PENK | 10 | 10 | 35.3 | 9 | 1 | 1 | 9 | ***90.0*** | ***90.0*** | ***Yes*** |
| US-CG84 | CHAD | 10 | 10 | 33.7 | 9 | 1 | 1 | 9 | ***90.0*** | ***90.0*** | ***Yes*** |
| US-CG85 | CHAD | 10 | 10 | 36.6 | 7 | 1 | 3 | 9 | 70.0 | 90.0 | ***No*** |
| US-CG90 | DRD4 | 10 | 10 | 34.8 | 5 | 1 | 5 | 9 | 50.0 | 90.0 | ***No*** |
| US-CG94 | MEIS1 | 10 | 10 | 33.0 | 9 | 1 | 1 | 9 | ***90.0*** | ***90.0*** | ***Yes*** |
| US-CG95 | NKX6-2 | 10 | 10 | 34.3 | 6 | 1 | 4 | 9 | 60.0 | 90.0 | ***No*** |
| US-CG96 | OTX1 | 10 | 10 | 33.9 | 6 | 1 | 4 | 9 | 60.0 | 90.0 | ***No*** |
| US-CG97 | OTX1 | 10 | 10 | 33.2 | 6 | 1 | 4 | 9 | 60.0 | 90.0 | ***No*** |

**Supplementary Table S5. Performance of top six biomarkers by SYBR green qMSP**

| **Gene** | **Specificity (%)** | **Sensitivity (%)** | **95% CI** | **AUC (95% CI)** |
| --- | --- | --- | --- | --- |
| *PENK* | 95 | 55.00 | 15.00–80.00 | 0.961 (0.847 to 0.997) |
| *DMRTA2* | 95 | 80.56 | 52.78–92.85 | 0.918 (0.832 to 0.969) |
| *CHAD* | 95 | 72.97 | 27.03–90.54 | 0.933 (0.855 to 0.972) |
| *CMTM2* | 95 | 37.50 | 12.50–62.50 | 0.714 (0.521 to 0.867) |
| DRD4 | 95 | 33.33 | 13.89–55.56 | 0.651 (0.521 to 0.773) |
| *MEISI* | 95 | 75.68 | 51.35–89.19 | 0.881 (0.771 to 0.945) |

**Supplementary Table S6. CT values obtained from urine-based methylaton test for two biomarkers: *DMRTA2* and *PENK***

| **Sample ID** | **Age** | **Sex** | **Group** | **TNM** | **CT.*ACTB**** | **CT.*DMRTA2*** | **CT.*PENK*** |
| --- | --- | --- | --- | --- | --- | --- | --- |
| B001 | 75 | Female | Bladder cancer | TaM0N0 | 18.24 | 30.2 | 31.67 |
| B003 | 99 | Male | Bladder cancer | TaN0M0 | 18.68 | 32 | 33.93 |
| B005 | 77 | Male | Bladder cancer | T1N0M0 | 18.51 | 30.06 | 30.67 |
| B012 | 41 | Male | Bladder cancer | T3bN0M0 | 17.88 | 30.27 | 29.97 |
| B014 | 71 | Male | Bladder cancer | T3aN1M0 | 18.67 | 32.53 | 34.74 |
| B020 | 72 | Male | Bladder cancer | T1N0M0 | 18.08 | 31.71 | 33.1 |
| B021 | 70 | Male | Bladder cancer | T1N0M0 | 19.75 | 34.63 | 36.28 |
| B024 | 69 | Male | Bladder cancer | T4aN0M0 | 18.66 | 34.96 | 35.46 |
| B025 | 61 | Male | Bladder cancer | T4aN0M0 | 21.09 | 37.22 | 45 |
| B026 | 67 | Male | Bladder cancer | T1N0M0 | 18.12 | 34.16 | 34.56 |
| B027 | 63 | Male | Bladder cancer | T1N0M0 | 19.03 | 34.01 | 35.5 |
| B029 | 78 | Male | Bladder cancer | T2N0M0 | 18.72 | 28.73 | 30.79 |
| B030 | 74 | Male | Bladder cancer | T1N0M0 | 23.16 | 36.23 | 38.14 |
| B031 | 62 | Male | Bladder cancer | T2aN0M0 | 18.11 | 29.06 | 32.71 |
| B032 | 79 | Male | Bladder cancer | TaN0M0 | 19.5 | 30.08 | 32.58 |
| B034 | 86 | Male | Bladder cancer | TaN0M0 | 18.47 | 36.85 | 35.95 |
| B037 | 76 | Female | Bladder cancer | NA | 18.66 | 34.94 | 35.87 |
| B038 | 73 | Male | Bladder cancer | T1N0M0 | 18.76 | 31.61 | 34.87 |
| B041 | 77 | Male | Bladder cancer | T1N0M0 | 23.07 | 35.75 | 36.12 |
| B043 | 67 | Male | Bladder cancer | TaN0M0 | 18.61 | 29.32 | 32.15 |
| B044 | 52 | Male | Bladder cancer | T2bN0M0 | 19.13 | 34.83 | 34.95 |
| B046 | 72 | Female | Bladder cancer | T1N0M0 | 18.52 | 29.87 | 30.51 |
| B051 | 82 | Male | Bladder cancer | TIN0M0 | 20.46 | 32.11 | 35.69 |
| B052 | 66 | Female | Bladder cancer | NA | 19 | 35.58 | 33.83 |
| B057 | 70 | Female | Bladder cancer | T3bN2M0 | 18.08 | 37.5 | 39.56 |
| B060 | 60 | Male | Bladder cancer | TaN0M0 | 18.7 | 31.95 | 30.13 |
| B062 | 79 | Male | Bladder cancer | T1N0M0 | 20.32 | 31.85 | 32.49 |
| B063 | 77 | Female | Bladder cancer | NA | 24.25 | 36.94 | 39.54 |
| B064 | 66 | Male | Bladder cancer | T1N0M0 | 18.19 | 31.17 | 30.07 |
| B067 | 62 | Male | Bladder cancer | T2aN0M0 | 18.23 | 29.11 | 32.99 |
| B068 | 80 | Male | Bladder cancer | T2M0M0 | 17.96 | 29.54 | 34.76 |
| B071 | 50 | Male | Bladder cancer | T1N0M0 | 17.99 | 29.24 | 29.54 |
| B076 | 89 | Male | Bladder cancer | TaN0M0 | 21.13 | 33.03 | 34.07 |
| B077 | 70 | Male | Bladder cancer | T1N0M0 | 19.8 | 34.57 | 36.7 |
| B081 | 64 | Male | Bladder cancer | T2bN0M0 | 17.8 | 30.59 | 31.98 |
| B082 | 66 | Male | Bladder cancer | TaN0M0 | 18.09 | 35.16 | 31.32 |
| B084 | 67 | Male | Bladder cancer | T1N0M0 | 17.75 | 29.7 | 34.2 |
| B086 | 84 | Male | Bladder cancer | T1N0M0 | 18.5 | 32.5 | 32.89 |
| B087 | 78 | Male | Bladder cancer | NA | 20.64 | 37.17 | 38.7 |
| B090 | 64 | Male | Bladder cancer | NA | 18.48 | 29.86 | 32.09 |
| B091 | 81 | Female | Bladder cancer | T2bN0M0 | 17.79 | 29.53 | 30.18 |
| B094 | 63 | Male | Bladder cancer | TaN0M0 | 20.31 | 45 | 45 |
| B095 | 70 | Male | Bladder cancer | TaN0M0 | 18.54 | 33.21 | 34.1 |
| B096 | 79 | Male | Bladder cancer | T1N0M0 | 17.98 | 29.41 | 31.22 |
| B099 | 70 | Male | Bladder cancer | TaN0M0 | 19.96 | 35.01 | 35.51 |
| B102 | 63 | Male | Bladder cancer | T1N0M0 | 34.5 | 45 | 45 |
| B103 | 63 | Male | Bladder cancer | TaN0M0 | 18.6 | 36.5 | 37.39 |
| B104 | 66 | Male | Bladder cancer | T1N0M0 | 18.46 | 31.05 | 32.31 |
| B106 | 52 | Female | Bladder cancer | T2N0M0 | 20.85 | 31.66 | 35.11 |
| B110 | 54 | Male | Bladder cancer | T2bN0M0 | 18.06 | 35.08 | 35.6 |
| B111 | 66 | Male | Bladder cancer | T2N2M0 | 18.61 | 30.01 | 31.53 |
| B112 | 78 | Male | Bladder cancer | T4aN0M0 | 17.68 | 28.75 | 30.89 |
| B113 | 54 | Male | Bladder cancer | TaM0N0 | 20.82 | 36.11 | 45 |
| B118 | 66 | Male | Bladder cancer | T1N0M0 | 18.09 | 33.2 | 33.27 |
| B122 | 66 | Male | Bladder cancer | T1N0M0 | 18.07 | 30.28 | 31.34 |
| B127 | 68 | Male | Bladder cancer | T2bN0M0 | 17.99 | 29.79 | 32.08 |
| B133 | 67 | Male | Bladder cancer | T1N0M0 | 22.51 | 34.94 | 36.77 |
| B135 | 81 | Male | Bladder cancer | T1N0M0 | 18.64 | 29.19 | 33.74 |
| B137 | 61 | Male | Bladder cancer | T4aN0M0 | 19.74 | 35.86 | 38.22 |
| B138 | 55 | Male | Bladder cancer | T1N0M0 | 18.11 | 29.92 | 34.21 |
| B141 | 56 | Male | Bladder cancer | TaN0M0 | 18.6 | 31.99 | 32.2 |
| B143 | 62 | Male | Bladder cancer | TaN0M0 | 18.72 | 32.98 | 33.48 |
| B145 | 73 | Male | Bladder cancer | T2N0M0 | 21.53 | 31.16 | 32.72 |
| B149 | 78 | Male | Bladder cancer | NA | 26.66 | 45 | 45 |
| B150 | 73 | Male | Bladder cancer | T1N0M0 | 18.72 | 33.26 | 34.32 |
| B151 | 81 | Male | Bladder cancer | T3aN0M0 | 17.99 | 28.87 | 35.98 |
| B154 | 67 | Male | Bladder cancer | T1N0M0 | 18.54 | 30.71 | 35.1 |
| B157 | 58 | Male | Bladder cancer | T1N0M0 | 18.09 | 31.03 | 33.75 |
| B158 | 72 | Male | Bladder cancer | T1N0M0 | 19.86 | 34.47 | 36.68 |
| B160 | 74 | Female | Bladder cancer | TaN0M0 | 18.65 | 30.64 | 30.92 |
| B163 | 78 | Male | Bladder cancer | T2bN0M0 | 18.48 | 30.67 | 30.96 |
| B164 | 56 | Male | Bladder cancer | TaN0M0 | 16.91 | 29.37 | 29.42 |
| B165 | 75 | Female | Bladder cancer | TaN0M0 | 18.12 | 29.78 | 29.83 |
| B166 | 44 | Male | Bladder cancer | T3N1M0 | 18.68 | 30.48 | 31.75 |
| B168 | 52 | Male | Bladder cancer | T2bN0M0 | 18.76 | 32.68 | 32.85 |
| B174 | 74 | Male | Bladder cancer | TaN0M0 | 15.49 | 28.14 | 28.81 |
| B175 | 70 | Male | Bladder cancer | NA | 18.03 | 30.48 | 30.88 |
| B176 | 66 | Male | Bladder cancer | T1N0M0 | 18.16 | 32.92 | 33.68 |
| B178 | 70 | Male | Bladder cancer | TaN0M0 | 18.06 | 28.83 | 31.15 |
| B179 | 69 | Male | Bladder cancer | T2aN0M0 | 18.05 | 30.97 | 36.39 |
| B183 | 58 | Male | Bladder cancer | TaN0M0 | 18.2 | 36.31 | 35.56 |
| B184 | 69 | Male | Bladder cancer | T2bN0M0 | 18.89 | 32.83 | 35.18 |
| B191 | 74 | Male | Bladder cancer | T1N0M0 | 18.34 | 36.1 | 36.67 |
| B193 | 59 | Female | Bladder cancer | TaN0M0 | 18.6 | 32.28 | 34.4 |
| B194 | 66 | Male | Bladder cancer | T4aN0M0 | 18.15 | 32.69 | 33.68 |
| B197 | 78 | Male | Bladder cancer | TaN0M0 | 17.56 | 36.99 | 37.63 |
| B198 | 54 | Male | Bladder cancer | T1N0M0 | 26.74 | 45 | 45 |
| B199 | 73 | Male | Bladder cancer | T1N0M0 | 29.62 | 45 | 45 |
| B200 | 62 | Male | Bladder cancer | TaN0M0 | 18.02 | 29.86 | 31.81 |
| B201 | 66 | Male | Bladder cancer | T1N0M0 | 17.87 | 34.54 | 32.61 |
| B206 | 79 | Male | Bladder cancer | TaN0M0 | 18.56 | 32.61 | 33.54 |
| B214 | 62 | Female | Bladder cancer | NA | 18.68 | 30.96 | 32.1 |
| B215 | 65 | Male | Bladder cancer | TaN0M0 | 18.2 | 33.5 | 34.28 |
| B218 | 88 | Male | Bladder cancer | TaN0M0 | 18.55 | 31.52 | 32.67 |
| B221 | 84 | Male | Bladder cancer | T2N0M0 | 18.11 | 31.15 | 32.89 |
| B222 | 62 | Male | Bladder cancer | NA | 18.03 | 29.16 | 29.61 |
| B225 | 70 | Male | Bladder cancer | TaN0M0 | 18.66 | 33.59 | 34.44 |
| B228 | 59 | Male | Bladder cancer | T2aN0M0 | 18.5 | 31.71 | 39.8 |
| B230 | 78 | Male | Bladder cancer | TaN0M0 | 18.59 | 29.57 | 32.29 |
| B232 | 72 | Male | Bladder cancer | NA | 23.83 | 45 | 45 |
| B002 | 64 | Female | Control |  | 23.97 | 45 | 45 |
| B004 | 81 | Male | Control |  | 17.88 | 37.56 | 45 |
| B006 | 29 | Male | Control |  | 18.76 | 36.85 | 37.46 |
| B007 | 43 | Female | Control |  | 20.42 | 38.6 | 45 |
| B008 | 49 | Male | Control |  | 27.86 | 45 | 45 |
| B009 | 54 | Female | Control |  | 20.46 | 45 | 45 |
| B010 | 60 | Male | Control |  | 18.95 | 37.06 | 45 |
| B011 | 49 | Male | Control |  | 20.75 | 45 | 45 |
| B013 | 69 | Male | Control |  | 18.01 | 37.73 | 36.06 |
| B015 | 45 | Female | Control |  | 18.8 | 38.47 | 38.43 |
| B016 | 51 | Male | Control |  | 18.14 | 38.5 | 45 |
| B017 | 57 | Male | Control |  | 29.04 | 45 | 45 |
| B018 | 60 | Male | Control |  | 17.23 | 37.33 | 38.27 |
| B019 | 60 | Male | Control |  | 18.15 | 37.8 | 45 |
| B022 | 47 | Female | Control |  | 19 | 38.62 | 39.73 |
| B023 | 56 | Male | Control |  | 20.57 | 37.42 | 36.81 |
| B028 | 52 | Male | Control |  | 22.63 | 45 | 45 |
| B033 | 38 | Male | Control |  | 18.43 | 37.68 | 38.15 |
| B035 | 59 | Male | Control |  | 18.18 | 37.69 | 45 |
| B036 | 0 | Female | Control |  | 18.48 | 45 | 45 |
| B039 | 0 | NA | Control |  | 19.02 | 37.92 | 38.69 |
| B040 | 80 | Male | Control |  | 18.44 | 37.99 | 45 |
| B042 | 43 | Male | Control |  | 18.54 | 38.66 | 45 |
| B045 | 33 | Female | Control |  | 18.82 | 37.17 | 45 |
| B047 | 52 | Male | Control |  | 18.73 | 34.94 | 35 |
| B048 | 49 | Male | Control |  | 20.74 | 38.62 | 45 |
| B049 | 63 | Female | Control |  | 18.97 | 37.95 | 45 |
| B050 | 44 | Male | Control |  | 19.05 | 45 | 45 |
| B053 | 42 | Female | Control |  | 18.46 | 37.7 | 45 |
| B054 | 47 | Male | Control |  | 18.76 | 38.19 | 45 |
| B055 | 47 | Male | Control |  | 18.53 | 45 | 45 |
| B056 | 32 | Male | Control |  | 28.1 | 45 | 45 |
| B058 | 40 | Male | Control |  | 23.94 | 45 | 45 |
| B059 | 58 | Female | Control |  | 18.42 | 45 | 45 |
| B061 | 45 | Female | Control |  | 19.35 | 45 | 39.7 |
| B065 | 78 | Male | Control |  | 19.75 | 38.4 | 45 |
| B066 | 72 | Male | Control |  | 17.81 | 37.47 | 45 |
| B069 | 73 | Female | Control |  | 18.66 | 35.93 | 35.54 |
| B070 | 68 | Male | Control |  | 19.52 | 45 | 39.92 |
| B072 | 61 | Male | Control |  | 18.9 | 34.15 | 34.55 |
| B073 | 58 | Female | Control |  | 18.99 | 37.57 | 40.13 |
| B074 | 50 | Female | Control |  | 22.63 | 45 | 45 |
| B075 | 26 | Female | Control |  | 18.65 | 38.92 | 45 |
| B078 | 76 | Female | Control |  | 18.45 | 37.89 | 45 |
| B079 | 43 | Male | Control |  | 18.53 | 36.84 | 37.73 |
| B080 | 48 | Female | Control |  | 18.96 | 38.46 | 39.19 |
| B083 | 44 | Male | Control |  | 18.57 | 34.56 | 36.53 |
| B085 | 29 | Female | Control |  | 18.55 | 36.49 | 45 |
| B088 | 69 | Male | Control |  | 20.05 | 37.26 | 39.04 |
| B089 | 63 | Female | Control |  | 17.78 | 38.57 | 40.51 |
| B092 | 69 | Male | Control |  | 17.92 | 36.53 | 37.16 |
| B093 | 80 | Male | Control |  | 17.75 | 45 | 38.65 |
| B097 | 26 | Female | Control |  | 19.44 | 38.29 | 45 |
| B098 | 55 | Male | Control |  | 21.85 | 40.71 | 45 |
| B100 | 50 | Male | Control |  | 19.34 | 45 | 45 |
| B101 | 0 | Female | Control |  | 19.49 | 37.42 | 40.9 |
| B105 | 57 | Male | Control |  | 20.56 | 39.18 | 40.13 |
| B107 | 69 | Male | Control |  | 18.8 | 37.49 | 37.54 |
| B108 | 74 | Male | Control |  | 18.71 | 32.95 | 34.69 |
| B109 | 45 | Male | Control |  | 19.04 | 45 | 45 |
| B114 | 80 | Male | Control |  | 18.7 | 37.55 | 45 |
| B115 | 65 | Male | Control |  | 19.34 | 36.66 | 38.24 |
| B116 | 74 | Male | Control |  | 18.86 | 38.06 | 37.79 |
| B117 | 51 | Male | Control |  | 22.72 | 45 | 45 |
| B119 | 53 | Male | Control |  | 21.17 | 45 | 45 |
| B120 | 73 | Male | Control |  | 18.17 | 37.51 | 45 |
| B121 | 54 | Male | Control |  | 28.97 | 45 | 45 |
| B123 | 50 | Female | Control |  | 19.73 | 38.4 | 37.32 |
| B124 | 68 | Male | Control |  | 23.63 | 45 | 38.81 |
| B125 | 82 | Male | Control |  | 16.21 | 36.57 | 37.76 |
| B126 | 51 | Male | Control |  | 25.22 | 45 | 45 |
| B128 | 45 | Male | Control |  | 22.82 | 41 | 45 |
| B129 | 62 | Male | Control |  | 18.03 | 38.15 | 45 |
| B130 | 41 | Male | Control |  | 19.04 | 45 | 45 |
| B131 | 46 | Female | Control |  | 18.83 | 37.49 | 37.2 |
| B132 | 60 | Female | Control |  | 21.66 | 45 | 45 |
| B134 | 65 | Male | Control |  | 18.18 | 36.51 | 38.18 |
| B136 | 44 | Female | Control |  | 22.73 | 39.81 | 45 |
| B139 | 73 | Male | Control |  | 20.71 | 45 | 45 |
| B140 | 63 | Male | Control |  | 21.19 | 45 | 45 |
| B142 | 74 | Male | Control |  | 19.6 | 38.08 | 38.09 |
| B144 | 0 |  | Control |  | 20.42 | 45 | 45 |
| B146 | 34 | Male | Control |  | 21.57 | 45 | 45 |
| B147 | 27 | Female | Control |  | 18.48 | 35.75 | 45 |
| B148 | 77 | Female | Control |  | 34.5 | 45 | 45 |
| B152 | 70 | Male | Control |  | 25.85 | 45 | 45 |
| B153 | 40 | Female | Control |  | 25.27 | 45 | 45 |
| B155 | 82 | Male | Control |  | 22.18 | 45 | 45 |
| B156 | 62 | Male | Control |  | 23.73 | 45 | 45 |
| B159 | 43 | Female | Control |  | 18.08 | 37.63 | 39.55 |
| B161 | 44 | Female | Control |  | 18.98 | 38.63 | 41.75 |
| B162 | 28 | Female | Control |  | 18.64 | 37.24 | 45 |
| B167 | 62 | Female | Control |  | 18.57 | 39.23 | 37.81 |
| B169 | 48 | Female | Control |  | 18.68 | 45 | 45 |
| B170 | 59 | Male | Control |  | 18.79 | 38.03 | 38.32 |
| B171 | 56 | Male | Control |  | 18.14 | 36.66 | 38.05 |
| B172 | 43 | Male | Control |  | 18.55 | 37.79 | 45 |
| B173 | 48 | Male | Control |  | 17.62 | 36.53 | 38.11 |
| B177 | 48 | Female | Control |  | 18.5 | 33.25 | 34.33 |
| B180 | 32 | Female | Control |  | 25.81 | 45 | 45 |
| B181 | 81 | Male | Control |  | 20.53 | 45 | 45 |
| B182 | 32 | Male | Control |  | 22.8 | 45 | 45 |
| B185 | 45 | Female | Control |  | 19.21 | 39.64 | 45 |
| B186 | 49 | Male | Control |  | 18.93 | 39.11 | 45 |
| B187 | 47 | Female | Control |  | 18.73 | 36.91 | 45 |
| B188 | 65 | Female | Control |  | 18.76 | 37.67 | 37.68 |
| B189 | 71 | Male | Control |  | 17.94 | 34.49 | 36.22 |
| B190 | 63 | Male | Control |  | 27.46 | 45 | 45 |
| B192 | 71 | Male | Control |  | 34.5 | 45 | 45 |
| B195 | 51 | Male | Control |  | 25.14 | 45 | 45 |
| B196 | 61 | Male | Control |  | 22.75 | 45 | 45 |
| B202 | 73 | Male | Control |  | 18.75 | 36.74 | 35.71 |
| B203 | 53 | Male | Control |  | 22.52 | 42.77 | 45 |
| B204 | 49 | Male | Control |  | 21.08 | 45 | 45 |
| B205 | 43 | Male | Control |  | 17.99 | 37.17 | 45 |
| B207 | 41 | Male | Control |  | 21.17 | 40.73 | 45 |
| B208 | 71 | Male | Control |  | 18.03 | 35.71 | 35.94 |
| B209 | 67 | Male | Control |  | 19.95 | 38.52 | 40.53 |
| B210 | 62 | Female | Control |  | 19.04 | 37.23 | 37.76 |
| B211 | 42 | Female | Control |  | 19.3 | 37.97 | 45 |
| B212 | 52 | Male | Control |  | 22.68 | 45 | 45 |
| B213 | 52 | Male | Control |  | 18.55 | 37.81 | 45 |
| B216 | 51 | Female | Control |  | 18.57 | 38.54 | 45 |
| B217 | 59 | Male | Control |  | 19.55 | 38.01 | 37.35 |
| B219 | 74 | Male | Control |  | 18.25 | 45 | 45 |
| B220 | 53 | Female | Control |  | 23.72 | 45 | 45 |
| B223 | 64 | Male | Control |  | 27.06 | 45 | 45 |
| B224 | 70 | Male | Control |  | 24.62 | 45 | 45 |
| B226 | 75 | Male | Control |  | 17.71 | 37.68 | 38.34 |
| B227 | 29 | Male | Control |  | 20.52 | 45 | 45 |
| B229 | 64 | Male | Control |  | 18.61 | 38.43 | 45 |
| B231 | 68 | Female | Control |  | 18.73 | 37.37 | 37.8 |
| B233 | 80 | Male | Control |  | 19.21 | 36.85 | 45 |
| B234 | 56 | Female | Control |  | 18.86 | 37.67 | 39.17 |
| B235 | 48 | Female | Control |  | 18.65 | 37.29 | 39.78 |
| B236 | 45 | Female | Control |  | 34.5 | 45 | 45 |
| B237 | 49 | Female | Control |  | 19 | 39.98 | 40.06 |

*****Reference gene was *ACTB*.

**Supplementary Table S7. Clinical features of the discovery cohort**

|  | **BC (*n* = 137)** | ***Non-BC (interfering cancers (*n* = 80))** | **benign tumors of bladder (*n* = 28)** | **Postoperative (*n* = 30)** | | **Normal (*n* = 202)** |
| --- | --- | --- | --- | --- | --- | --- |
|  |  |  |  | healthy (*n* = 8) | recurring cancers (*n* = 22) |  |
| **Age, years** |  |  |  |  | |  |
| Median (range) | 67 (41–99) | 62 (43–82) | 61 (42–85) | 62 (56–82) | 66 (44–82) | 62 (41–85) |
| <60 | 27 (19.7%) | 28 (35.0%) | 13 (46.4%) | 3 (37.5%) | 6 (27.3%) | 85 (42.1%) |
| ≥60 | 110 (80.3%) | 52 (65.0%) | 15 (53.6%) | 5 (62.5%) | 16 (72.7%) | 117 (57.9%) |
| **Sex, n (%)** |  |  |  |  |  |  |
| Male | 117 (78.4%) | 51 (63.8%) | 23 (66.7%) | 6 (75.0%) | 19 (86.4%) | 133 (59.1%) |
| Female | 20 (21.6%) | 29 (36.2%) | 5 (33.3%) | 2 (25.0%) | 3 (13.6%) | 69 (40.9%) |
| **Stage, n (%)** |  |  |  |  |  |  |
| Ta/T1 | 84 (61.3%) | NA | NA | NA | 15 (68.2%) | NA |
| T2/T3/T4 | 44 (32.1%) | NA | NA | NA | 5 (22.7%) | NA |
| NA | 9 (6.6%) | NA | NA | NA | 2 (9.1%) | NA |
| **Grade, n (%)** |  |  | NA |  |  |  |
| low | 47 (34.3%) | NA | NA | NA | 9(40.9%) | NA |
| High | 78 (56.9%) | NA | NA | NA | 11(50.0%) | NA |
| NA | 12 (8.8%) | NA | NA | NA | 2(9.1%) | NA |
| *Non-BC (interfering cancers (*n* = 80)): carcinomas of renal pelvis (*n* = 23), carcinomas of ureter (*n* = 13), renal carcinomas (*n* = 31), prostate cancers (*n* = 13) | | | | | | |

**Supplementary Table S8. CT values obtained from qMSP of *DMRTA2* for urine samples of the discovery set**

| **Sample ID** | **Age** | **Sex** | **Group** | **TNM** | **CT.*ACTB*** | **CT.*DMRTA2*** |
| --- | --- | --- | --- | --- | --- | --- |
| UA002 | 61 | Male | Bladder cancer | T4aN0M0 | 28.75 | 35.84 |
| UA006 | 66 | Male | Bladder cancer | T1N0M0 | 28.13 | 39.57 |
| UA053 | 69 | Male | Bladder cancer | T2aN0M0 | 28.32 | 32.56 |
| UA056 | 70 | Male | Bladder cancer | TaN0M0 | 28.22 | 30.12 |
| UA060 | 79 | Male | Bladder cancer | TaN0M0 | 28.56 | 35.59 |
| UA073 | 55 | Male | Bladder cancer | T1N0M0 | 28.56 | 31.14 |
| UA074 | 67 | Male | Bladder cancer | T1N0M0 | 31.78 | 33.97 |
| UA075 | 61 | Male | Bladder cancer | T4aN0M0 | 30.04 | 43 |
| UA077 | 66 | Male | Bladder cancer | T4aN0M0 | 28.06 | 33.58 |
| UA078 | 62 | Male | Bladder cancer | TaN0M0 | 28.1 | 31.52 |
| UA091 | 78 | Male | Bladder cancer | TaN0M0 | 28.91 | 33.45 |
| UA092 | 67 | Male | Bladder cancer | T1N0M0 | 28.3 | 30.84 |
| UA094 | 79 | Male | Bladder cancer | T1N0M0 | 29.76 | 32.03 |
| UA097 | 79 | Male | Bladder cancer | T1N0M0 | 27.98 | 29.95 |
| UA104 | 70 | Female | Bladder cancer | T3bN2M0 | 28.1 | 40 |
| UA119 | 73 | Male | Bladder cancer | T1N0M0 | 28.44 | 34.87 |
| UA122 | 66 | Male | Bladder cancer | T1N0M0 | 28.33 | 31.13 |
| UA124 | 56 | Male | Bladder cancer | TaN0M0 | 28.05 | 32.98 |
| UA130 | 81 | Male | Bladder cancer | T1N0M0 | 28.33 | 34.24 |
| UA134 | 67 | Male | Bladder cancer | TaN0M0 | 28.59 | 31.8 |
| UA135 | 86 | Male | Bladder cancer | TaN0M0 | 28.44 | 39.03 |
| UA137 | 73 | Male | Bladder cancer | T2N0M0 | 32.29 | 32.5 |
| UA139 | 78 | Male | Bladder cancer | T4aN0M0 | 28.05 | 29.72 |
| UA147 | 63 | Male | Bladder cancer | TaN0M0 | 29.54 | 43 |
| UA149 | 50 | Male | Bladder cancer | T1N0M0 | 28.53 | 29.79 |
| UA154 | 66 | Male | Bladder cancer | T1N0M0 | 28.56 | 32.96 |
| UA156 | 71 | Male | Bladder cancer | T4aN0M0 | 28.76 | 40 |
| UA157 | 82 | Male | Bladder cancer | TaN0M0 | 28.78 | 30.82 |
| UA159 | 72 | Male | Bladder cancer | TaN0M0 | 27.91 | 33.31 |
| UA162 | 72 | Female | Bladder cancer | T2N0M0 | 28.62 | 32.98 |
| UA168 | 62 | Male | Bladder cancer | T2bN0M0 | 27.95 | 32.13 |
| UA171 | 69 | Male | Bladder cancer | T1N0M0 | 28.6 | 32.46 |
| UA173 | 60 | Male | Bladder cancer | TaN0M0 | 28.65 | 29.73 |
| UA174 | 74 | Male | Bladder cancer | NA | 27.92 | 34.35 |
| UA191 | 56 | Male | Bladder cancer | TaN0M0 | 28.12 | 38.89 |
| UA199 | 47 | Male | Bladder cancer | TaN0M0 | 28.14 | 43 |
| UD0009 | 68 | Male | Bladder cancer | T2bN0M0 | 28.04 | 30.51 |
| UD0011 | 81 | Male | Bladder cancer | T3aN0M0 | 28.51 | 29.86 |
| UD0012 | 46 | Male | Bladder cancer | T3aN0M0 | 28.24 | 34.73 |
| UD0014 | 66 | Male | Bladder cancer | T1N0M0 | 28.1 | 33 |
| UD0015 | 66 | Male | Bladder cancer | T1N0M0 | 28.1 | 35.08 |
| UD0018 | 82 | Male | Bladder cancer | T1N0M0 | 30.28 | 32.72 |
| UD0028 | 67 | Male | Bladder cancer | T1N0M0 | 28 | 34.13 |
| UD0030 | 78 | Male | Bladder cancer | T2N0M0 | 28.89 | 29.15 |
| UD0038 | 80 | Male | Bladder cancer | T2N0M0 | 28.1 | 29.98 |
| UD0039 | 58 | Male | Bladder cancer | TaN0M0 | 28.1 | 38.1 |
| UD0043 | 79 | Male | Bladder cancer | NA | 28.09 | 37.96 |
| UD0044 | 52 | Male | Bladder cancer | T2N0MO | 28.3 | 31.73 |
| UD0046 | 50 | Male | Bladder cancer | T1N0M0 | 28.23 | 34.72 |
| UD0047 | 64 | Male | Bladder cancer | T2aN0M0 | 28.22 | 30.82 |
| UD0049 | 81 | Male | Bladder cancer | T1N0M0 | 28.21 | 37.92 |
| UD0051 | 61 | Male | Bladder cancer | T2N0M0 | 28.48 | 30.37 |
| UD0054 | 68 | Male | Bladder cancer | TaN0M0 | 28.24 | 32.92 |
| UD0056 | 70 | Female | Bladder cancer | T1N0M0 | 28.22 | 30.56 |
| UD0057 | 76 | Male | Bladder cancer | T1N0M0 | 28.5 | 29.43 |
| UD0059 | 62 | Male | Bladder cancer | T2bN2M0 | 28.09 | 33.83 |
| UD0061 | 88 | Male | Bladder cancer | TaN0M0 | 28.33 | 31.98 |
| UD0063 | 63 | Male | Bladder cancer | T2N0M0 | 28.04 | 31.91 |
| UD0070 | 69 | Male | Bladder cancer | T1N0M0 | 28.15 | 33.65 |
| UD0072 | 68 | Male | Bladder cancer | TaN0M0 | 30.84 | 37.95 |
| UD0076 | 56 | Male | Bladder cancer | T1N1M0 | 28.34 | 30.65 |
| UD0089 | 57 | Male | Bladder cancer | TaN0M0 | 28.21 | 40 |
| UD0095 | 84 | Male | Bladder cancer | T2N0M0 | 28.2 | 31.45 |
| UD0099 | 54 | Male | Bladder cancer | T2bN0M0 | 28.44 | 37.32 |
| UD0105 | 64 | Male | Bladder cancer | T3aN0M0 | 29.01 | 37.93 |
| UD0109 | 62 | Female | Bladder cancer | NA | 28.21 | 31.06 |
| UD0110 | 74 | Male | Bladder cancer | T1N0M0 | 28.21 | 36.71 |
| UD0135 | 70 | Male | Bladder cancer | TaN0M0 | 28.1 | 33.93 |
| UD0142 | 70 | Male | Bladder cancer | T1N0M0 | 28.83 | 35.62 |
| UD0161 | 66 | Male | Bladder cancer | T2N2M0 | 28.5 | 30.24 |
| UD0162 | 70 | Male | Bladder cancer | TaN0M0 | 28.68 | 35.73 |
| UD0164 | 63 | Male | Bladder cancer | TaN0M0 | 28.27 | 37.75 |
| UD0167 | 75 | Female | Bladder cancer | TaN0M0 | 28.06 | 29.81 |
| UD0175 | 52 | Male | Bladder cancer | T2bN0M0 | 28.23 | 32.63 |
| UD0221 | 62 | Male | Bladder cancer | T2aN0M0 | 28.01 | 29.04 |
| UD0225 | 78 | Male | Bladder cancer | NA | 29.14 | 35.42 |
| UD0237 | 89 | Male | Bladder cancer | TaN0M0 | 30.52 | 33.62 |
| UD0244 | 41 | Male | Bladder cancer | T3bN0M0 | 28.17 | 31.07 |
| UD0250 | 77 | Male | Bladder cancer | T1N0M0 | 32.31 | 35.15 |
| UD0257 | 72 | Female | Bladder cancer | T1N0M0 | 28.2 | 30.32 |
| UD0293 | 71 | Male | Bladder cancer | T3aN1M0 | 28.51 | 33.07 |
| UD0298 | 44 | Male | Bladder cancer | T3N1M0 | 28.46 | 30.69 |
| UD0303 | 62 | Male | Bladder cancer | TaN0M0 | 28.44 | 35.85 |
| UD0308 | 69 | Male | Bladder cancer | T4aN0M0 | 28.06 | 34.62 |
| UD0314 | 60 | Male | Bladder cancer | TaN0M0 | 28.58 | 34.01 |
| UD0330 | 77 | Male | Bladder cancer | T1N0M0 | 28.42 | 30.3 |
| UD0348 | 74 | Male | Bladder cancer | T1N0M0 | 31.24 | 35.19 |
| UD0349 | 84 | Male | Bladder cancer | T1N0M0 | 28.63 | 34.56 |
| UD0350 | 52 | Female | Bladder cancer | T2N0M0 | 29.35 | 30.71 |
| UD0355 | 64 | Male | Bladder cancer | T2bN0M0 | 28.32 | 32.34 |
| UD0366 | 73 | Male | Bladder cancer | T1N0M0 | 28.59 | 31.6 |
| UD0367 | 59 | Male | Bladder cancer | T2aN0M0 | 28.51 | 31.82 |
| UD0380 | 67 | Male | Bladder cancer | T2bN3M0 | 28.55 | 31.72 |
| UD0401 | 82 | Male | Bladder cancer | T2N0M0 | 28.31 | 28.95 |
| UD0407 | 56 | Male | Bladder cancer | T2bN0M0 | 28.48 | 33.89 |
| UD0413 | 45 | Female | Bladder cancer | TaN0M0 | 31.13 | 43 |
| UD0439 | 62 | Male | Bladder cancer | T1N0M0 | 28.61 | 34.62 |
| UD0440 | 56 | Female | Bladder cancer | T1N0M0 | 28.6 | 35.23 |
| UD0458 | 82 | Male | Bladder cancer | T1N0M0 | 28.1 | 32.21 |
| UD0473 | 52 | Female | Bladder cancer | T1N0M0 | 28.58 | 32.06 |
| UD0477 | 55 | Male | Bladder cancer | T3N1M0 | 28.61 | 32.72 |
| UD0486 | 72 | Male | Bladder cancer | T3N2M0 | 28.06 | 30.82 |
| UD0489 | 75 | Male | Bladder cancer | T2bN0M0 | 28.47 | 31.85 |
| UD0503 | 71 | Male | Bladder cancer | T1N0M0 | 28.45 | 31.65 |
| UD0521 | 51 | Female | Bladder cancer | T1N0M0 | 28.47 | 30.78 |
| UD0540 | 58 | Male | Bladder cancer | T1N0M0 | 28.09 | 33.86 |
| UD0550 | 63 | Male | Bladder cancer | T1N0M0 | 28.23 | 35.08 |
| UD0564 | 78 | Male | Bladder cancer | T1N0M0 | 28.28 | 29.53 |
| UD0569 | 62 | Female | Bladder cancer | TaN0M0 | 29.95 | 43 |
| UD0600 | 65 | Female | Bladder cancer | T1N0M0 | 29.52 | 31.18 |
| UD0617 | 80 | Male | Bladder cancer | T1N0M0 | 28.22 | 29.83 |
| UD0619 | 72 | Male | Bladder cancer | TaN0M0 | 28.13 | 30.76 |
| UD0625 | 63 | Male | Bladder cancer | T1N0M0 | 28.08 | 31.72 |
| UD0646 | 64 | Male | Bladder cancer | T1N0M0 | 30.24 | 32.26 |
| UD0651 | 66 | Male | Bladder cancer | T2bN0M0 | 28.54 | 35.2 |
| UD0653 | 71 | Female | Bladder cancer | T1N0M0 | 28.49 | 35.03 |
| UD0666 | 72 | Male | Bladder cancer | T1N0M0 | 28.7 | 30.21 |
| UD0698 | 67 | Male | Bladder cancer | T1N0M0 | 27.92 | 29.58 |
| UD0701 | 65 | Male | Bladder cancer | T4aN0M0 | 28.53 | 30.18 |
| UD0702 | 64 | Female | Bladder cancer | T1N0M0 | 28.59 | 29.94 |
| UD0748 | 73 | Male | Bladder cancer | TaN0M0 | 28.54 | 35.76 |
| UD0798 | 76 | Female | Bladder cancer | T1N0M0 | 28.44 | 33.12 |
| UD0804 | 77 | Male | Bladder cancer | T2N0M0 | 28.29 | 29.37 |
| UD0812 | 52 | Male | Bladder cancer | TaN0M0 | 28.16 | 40 |
| UD0827 | 58 | Male | Bladder cancer | T1N0M0 | 28.3 | 43 |
| UD0850 | 79 | Male | Bladder cancer | T1N0M0 | 28.04 | 28.1 |
| UD0860 | 70 | Male | Bladder cancer | T1N0M0 | 28.13 | 30.29 |
| UG003 | 59 | Female | Bladder cancer | TaN0M0 | 28.31 | 32.06 |
| UG025 | 66 | Female | Bladder cancer | NA | 28.12 | 36.92 |
| UG046 | 76 | Female | Bladder cancer | NA | 28.58 | 35.91 |
| UG053 | 65 | Male | Bladder cancer | TaN0M0 | 28.17 | 34.23 |
| UG059 | 78 | Male | Bladder cancer | T2bN0M0 | 28.24 | 30.49 |
| UG064 | 69 | Male | Bladder cancer | T2bN0M0 | 28.95 | 33.85 |
| UG072 | 69 | Male | Bladder cancer | NA | 28.51 | 29.15 |
| UG074 | 64 | Female | Bladder cancer | NA | 28.49 | 31.08 |
| UG080 | 99 | Male | Bladder cancer | TaN0M0 | 28.48 | 34.45 |
| UG083 | 70 | Male | Bladder cancer | NA | 29.78 | 33.02 |
| UA057 | 69 | Male | Control |  | 29.46 | 40 |
| UA058 | 51 | Male | Control |  | 27.92 | 40 |
| UA108 | 43 | Male | Control |  | 28.32 | 38.23 |
| UA109 | 41 | Male | Control |  | 29.66 | 37.92 |
| UA114 | 72 | Male | Control |  | 28.33 | 40 |
| UA117 | 44 | Female | Control |  | 28.79 | 43 |
| UA182 | 57 | Male | Control |  | 30.65 | 38.33 |
| UA188 | 71 | Male | Control |  | 28.67 | 43 |
| UA194 | 85 | Male | Control |  | 28.67 | 40 |
| UD0040 | 49 | Female | Control |  | 28.2 | 43 |
| UD0041 | 73 | Female | Control |  | 28.24 | 37.72 |
| UD0050 | 47 | Male | Control |  | 28.09 | 43 |
| UD0073 | 59 | Male | Control |  | 28.17 | 43 |
| UD0108 | 80 | Male | Control |  | 29.05 | 40 |
| UD0124 | 57 | Male | Control |  | 28.53 | 36.14 |
| UD0126 | 69 | Male | Control |  | 28.25 | 38.28 |
| UD0200 | 53 | Male | Control |  | 30.25 | 43 |
| UD0236 | 80 | Male | Control |  | 28.31 | 43 |
| UD0238 | 75 | Male | Control |  | 28.06 | 43 |
| UD0259 | 71 | Male | Control |  | 28.51 | 36.61 |
| UD0263 | 54 | Female | Control |  | 29.51 | 40 |
| UD0265 | 68 | Male | Control |  | 29.58 | 43 |
| UD0266 | 67 | Male | Control |  | 28.57 | 43 |
| UD0269 | 56 | Male | Control |  | 29.62 | 36.91 |
| UD0270 | 65 | Male | Control |  | 28.58 | 35.92 |
| UD0274 | 51 | Female | Control |  | 28.49 | 43 |
| UD0275 | 64 | Male | Control |  | 28.22 | 40 |
| UD0276 | 51 | Male | Control |  | 31.48 | 43 |
| UD0281 | 62 | Female | Control |  | 28.19 | 43 |
| UD0285 | 53 | Female | Control |  | 31.41 | 43 |
| UD0289 | 51 | Male | Control |  | 31.97 | 43 |
| UD0300 | 57 | Male | Control |  | 33.3 | 43 |
| UD0301 | 74 | Male | Control |  | 28.22 | 43 |
| UD0316 | 63 | Female | Control |  | 27.61 | 43 |
| UD0318 | 80 | Male | Control |  | 29.51 | 38.16 |
| UD0323 | 49 | Male | Control |  | 28.78 | 43 |
| UD0325 | 43 | Male | Control |  | 28.8 | 43 |
| UD0326 | 43 | Female | Control |  | 29.98 | 40 |
| UD0328 | 55 | Male | Control |  | 30.91 | 43 |
| UD0331 | 76 | Female | Control |  | 28.28 | 40 |
| UD0336 | 74 | Male | Control |  | 29.16 | 37.07 |
| UD0340 | 48 | Female | Control |  | 30.69 | 43 |
| UD0346 | 80 | Male | Control |  | 28.53 | 40 |
| UD0353 | 46 | Female | Control |  | 34.55 | 43 |
| UD0354 | 54 | Female | Control |  | 28.54 | 39.06 |
| UD0357 | 60 | Male | Control |  | 28.41 | 43 |
| UD0359 | 67 | Female | Control |  | 32.76 | 43 |
| UD0363 | 56 | Male | Control |  | 32.08 | 43 |
| UD0364 | 81 | Male | Control |  | 31.78 | 43 |
| UD0372 | 61 | Male | Control |  | 28.74 | 40 |
| UD0382 | 51 | Male | Control |  | 28.52 | 40 |
| UD0383 | 67 | Male | Control |  | 28.29 | 40 |
| UD0386 | 51 | Female | Control |  | 29.6 | 43 |
| UD0390 | 70 | Male | Control |  | 29.42 | 40 |
| UD0391 | 55 | Male | Control |  | 28.53 | 43 |
| UD0392 | 59 | Female | Control |  | 28.76 | 43 |
| UD0393 | 60 | Male | Control |  | 31.42 | 43 |
| UD0394 | 55 | Male | Control |  | 29.01 | 40 |
| UD0398 | 43 | Female | Control |  | 28.89 | 40 |
| UD0400 | 53 | Female | Control |  | 29.18 | 43 |
| UD0402 | 62 | Female | Control |  | 28.83 | 40 |
| UD0405 | 50 | Female | Control |  | 28.53 | 38.19 |
| UD0408 | 72 | Male | Control |  | 28.75 | 37.86 |
| UD0411 | 72 | Male | Control |  | 28.21 | 39.05 |
| UD0416 | 67 | Male | Control |  | 28.46 | 37.79 |
| UD0422 | 77 | Female | Control |  | 28.23 | 40 |
| UD0425 | 65 | Male | Control |  | 29.72 | 40 |
| UD0426 | 50 | Female | Control |  | 28.27 | 40 |
| UD0430 | 62 | Male | Control |  | 28.22 | 40 |
| UD0433 | 75 | Female | Control |  | 28.33 | 40 |
| UD0434 | 52 | Male | Control |  | 32.52 | 43 |
| UD0436 | 81 | Male | Control |  | 28.47 | 40 |
| UD0437 | 68 | Female | Control |  | 29.28 | 40 |
| UD0441 | 72 | Male | Control |  | 28.72 | 40 |
| UD0444 | 63 | Male | Control |  | 28.27 | 43 |
| UD0446 | 68 | Male | Control |  | 30.61 | 40 |
| UD0447 | 69 | Male | Control |  | 28.29 | 40 |
| UD0449 | 60 | Female | Control |  | 31.63 | 43 |
| UD0454 | 60 | Male | Control |  | 28.71 | 40 |
| UD0459 | 54 | Female | Control |  | 28.43 | 43 |
| UD0465 | 50 | Female | Control |  | 29.01 | 43 |
| UD0467 | 45 | Male | Control |  | 28.23 | 34.41 |
| UD0470 | 62 | Female | Control |  | 27.75 | 43 |
| UD0474 | 63 | Male | Control |  | 28.43 | 43 |
| UD0478 | 55 | Male | Control |  | 28.17 | 43 |
| UD0488 | 69 | Female | Control |  | 29.14 | 40 |
| UD0490 | 79 | Male | Control |  | 28.5 | 36.14 |
| UD0491 | 64 | Female | Control |  | 28.51 | 40 |
| UD0493 | 54 | Male | Control |  | 28.08 | 43 |
| UD0496 | 69 | Female | Control |  | 28.84 | 43 |
| UD0504 | 44 | Male | Control |  | 31.22 | 43 |
| UD0511 | 50 | Male | Control |  | 28.27 | 43 |
| UD0514 | 55 | Male | Control |  | 29.77 | 43 |
| UD0515 | 55 | Female | Control |  | 28.16 | 40 |
| UD0516 | 54 | Male | Control |  | 34.89 | 43 |
| UD0517 | 78 | Male | Control |  | 28.14 | 40 |
| UD0525 | 76 | Male | Control |  | 28.52 | 43 |
| UD0531 | 72 | Male | Control |  | 29.22 | 40 |
| UD0534 | 71 | Female | Control |  | 28.54 | 40 |
| UD0535 | 67 | Male | Control |  | 28.02 | 43 |
| UD0536 | 57 | Male | Control |  | 31.59 | 43 |
| UD0538 | 68 | Male | Control |  | 32.62 | 43 |
| UD0539 | 77 | Male | Control |  | 28.21 | 40 |
| UD0541 | 49 | Male | Control |  | 28.31 | 39.61 |
| UD0544 | 67 | Female | Control |  | 28.2 | 38.44 |
| UD0545 | 54 | Male | Control |  | 28.22 | 43 |
| UD0551 | 56 | Male | Control |  | 28.3 | 43 |
| UD0554 | 55 | Male | Control |  | 28.62 | 34.31 |
| UD0563 | 76 | Male | Control |  | 28.2 | 36.63 |
| UD0566 | 77 | Male | Control |  | 27.86 | 38.27 |
| UD0570 | 68 | Male | Control |  | 32.43 | 43 |
| UD0576 | 56 | Female | Control |  | 28.18 | 38.13 |
| UD0579 | 63 | Female | Control |  | 28.08 | 43 |
| UD0580 | 67 | Male | Control |  | 27.86 | 40 |
| UD0584 | 67 | Male | Control |  | 27.93 | 35.24 |
| UD0588 | 51 | Male | Control |  | 28.08 | 43 |
| UD0591 | 51 | Male | Control |  | 27.86 | 43 |
| UD0596 | 48 | Female | Control |  | 30.21 | 43 |
| UD0597 | 80 | Male | Control |  | 31.06 | 40 |
| UD0602 | 61 | Male | Control |  | 28 | 40 |
| UD0603 | 55 | Female | Control |  | 28.14 | 38.49 |
| UD0606 | 61 | Female | Control |  | 28.32 | 40 |
| UD0607 | 63 | Male | Control |  | 27.81 | 43 |
| UD0608 | 66 | Female | Control |  | 28.81 | 40 |
| UD0611 | 65 | Female | Control |  | 28.18 | 34.23 |
| UD0612 | 63 | Male | Control |  | 28.43 | 31.84 |
| UD0621 | 72 | Male | Control |  | 28.02 | 38.54 |
| UD0627 | 70 | Male | Control |  | 27.96 | 40 |
| UD0631 | 69 | Female | Control |  | 27.99 | 32.27 |
| UD0636 | 73 | Male | Control |  | 28.04 | 40 |
| UD0641 | 57 | Male | Control |  | 33.32 | 43 |
| UD0642 | 75 | Male | Control |  | 28.59 | 33.89 |
| UD0647 | 62 | Female | Control |  | 29.47 | 43 |
| UD0649 | 73 | Male | Control |  | 29.13 | 40 |
| UD0652 | 52 | Female | Control |  | 28.22 | 40 |
| UD0656 | 53 | Male | Control |  | 28.96 | 43 |
| UD0662 | 74 | Male | Control |  | 33.92 | 43 |
| UD0663 | 70 | Male | Control |  | 28.26 | 40 |
| UD0667 | 68 | Female | Control |  | 28.23 | 40 |
| UD0669 | 50 | Female | Control |  | 28.41 | 43 |
| UD0672 | 69 | Male | Control |  | 28.53 | 39.07 |
| UD0676 | 55 | Male | Control |  | 28.72 | 40 |
| UD0677 | 66 | Male | Control |  | 29.18 | 43 |
| UD0680 | 70 | Male | Control |  | 28.43 | 40 |
| UD0683 | 59 | Male | Control |  | 29.2 | 43 |
| UD0690 | 73 | Male | Control |  | 28.47 | 43 |
| UD0692 | 70 | Male | Control |  | 29.61 | 40 |
| UD0696 | 63 | Male | Control |  | 28.24 | 40 |
| UD0700 | 71 | Male | Control |  | 28.31 | 35.77 |
| UD0703 | 70 | Male | Control |  | 28.64 | 40 |
| UD0709 | 54 | Male | Control |  | 28.69 | 43 |
| UD0711 | 73 | Male | Control |  | 34.83 | 43 |
| UD0712 | 69 | Male | Control |  | 28.07 | 43 |
| UD0714 | 45 | Female | Control |  | 28.98 | 43 |
| UD0715 | 50 | Female | Control |  | 28.67 | 37.97 |
| UD0726 | 52 | Male | Control |  | 28.31 | 40 |
| UD0730 | 74 | Male | Control |  | 29.76 | 40 |
| UD0731 | 56 | Male | Control |  | 28.5 | 40 |
| UD0732 | 76 | Male | Control |  | 29.55 | 43 |
| UD0734 | 66 | Female | Control |  | 30.62 | 40 |
| UD0735 | 75 | Female | Control |  | 28.04 | 38.71 |
| UD0742 | 46 | Male | Control |  | 28.23 | 43 |
| UD0743 | 55 | Female | Control |  | 29.2 | 40 |
| UD0745 | 55 | Male | Control |  | 28.78 | 43 |
| UD0746 | 50 | Male | Control |  | 28.6 | 43 |
| UD0749 | 61 | Male | Control |  | 28.46 | 43 |
| UD0758 | 50 | Male | Control |  | 28.96 | 43 |
| UD0759 | 61 | Male | Control |  | 29.19 | 43 |
| UD0762 | 43 | Female | Control |  | 28.43 | 40 |
| UD0763 | 80 | Male | Control |  | 28.48 | 40 |
| UD0765 | 59 | Male | Control |  | 28.46 | 43 |
| UD0767 | 42 | Male | Control |  | 28.5 | 43 |
| UD0768 | 58 | Female | Control |  | 27.99 | 38.53 |
| UD0778 | 62 | Male | Control |  | 28.33 | 40 |
| UD0781 | 69 | Male | Control |  | 29.46 | 40 |
| UD0784 | 49 | Female | Control |  | 29.17 | 43 |
| UD0792 | 70 | Male | Control |  | 29.73 | 38.09 |
| UD0795 | 45 | Female | Control |  | 28.48 | 40 |
| UD0796 | 69 | Male | Control |  | 30.25 | 43 |
| UD0799 | 67 | Female | Control |  | 28.68 | 40 |
| UD0805 | 62 | Female | Control |  | 28.1 | 40 |
| UD0811 | 68 | Female | Control |  | 27.98 | 43 |
| UD0814 | 54 | Female | Control |  | 28.07 | 39.47 |
| UD0817 | 60 | Female | Control |  | 28.13 | 40 |
| UD0819 | 53 | Male | Control |  | 28.25 | 43 |
| UD0822 | 71 | Male | Control |  | 27.97 | 37.3 |
| UD0826 | 68 | Female | Control |  | 28.21 | 40 |
| UD0828 | 67 | Female | Control |  | 28.24 | 40 |
| UD0830 | 51 | Female | Control |  | 28.64 | 40 |
| UD0832 | 54 | Male | Control |  | 28.12 | 40 |
| UD0835 | 51 | Female | Control |  | 34.63 | 43 |
| UD0836 | 52 | Female | Control |  | 27.86 | 38.33 |
| UD0839 | 63 | Female | Control |  | 28.08 | 43 |
| UD0846 | 52 | Female | Control |  | 28.53 | 43 |
| UD0847 | 62 | Female | Control |  | 28.02 | 43 |
| UD0851 | 66 | Male | Control |  | 27.87 | 37.34 |
| UG004 | 63 | Female | Control |  | 28.14 | 43 |
| UG066 | 74 | Male | Control |  | 28.16 | 43 |
| UG067 | 70 | Male | Control |  | 28.27 | 43 |
| UG091 | 67 | Male | Control |  | 30.13 | 40 |
| UG092 | 68 | Male | Control |  | 31.67 | 43 |
| UG093 | 50 | Male | Control |  | 29.16 | 40 |
| UA054 | 71 | Male | prostate cancers | pMIc | 28.28 | 40 |
| UA143 | 62 | Male | Renal carcinomas | T1bN0M0 | 28.19 | 40 |
| UA160 | 43 | Female | Renal carcinomas | T1bN0M0 | 31.63 | 43 |
| UA166 | 55 | Female | Renal carcinomas | T1bN0M0 | 29.28 | 43 |
| UA167 | 55 | Male | Renal carcinomas | T1aN0M0 | 29.9 | 43 |
| UD0086 | 55 | Female | Renal carcinomas | T1aN0M0 | 28.63 | 39.03 |
| UD0088 | 61 | Female | Renal carcinomas | T2aN0M0 | 28.28 | 40 |
| UD0090 | 61 | Female | Renal carcinomas | T1aN0M0 | 30.02 | 37.05 |
| UD0092 | 72 | Male | Renal carcinomas | T1bN0M0 | 30.88 | 43 |
| UD0107 | 60 | Male | Renal carcinomas | T1bN0M0 | 29.72 | 43 |
| UD0116 | 66 | Male | Renal carcinomas | T1aN0M0 | 28.76 | 40 |
| UD0118 | 64 | Male | Renal carcinomas | T1bN0M0 | 29.75 | 40 |
| UD0122 | 51 | Male | Renal carcinomas | T3aN0M0 | 28.1 | 43 |
| UD0153 | 52 | Female | Renal carcinomas | T2aN0M0 | 28.29 | 40 |
| UD0166 | 65 | Female | Renal carcinomas | T2aN0M0 | 28.72 | 40 |
| UD0193 | 50 | Male | Renal carcinomas | T1aN0M0 | 33.29 | 43 |
| UD0203 | 52 | Female | Renal carcinomas | T2bN0M0 | 30.53 | 43 |
| UD0207 | 56 | Female | Renal carcinomas | T1aN0M0 | 33.8 | 43 |
| UD0216 | 48 | Male | Renal carcinomas | T2aN0M0 | 28.28 | 40 |
| UD0229 | 46 | Female | Renal carcinomas | T1aN0M0 | 28.09 | 37.74 |
| UD0248 | 71 | Female | Renal carcinomas | T3aN0M0 | 30.02 | 39.1 |
| UD0271 | 59 | Female | Renal carcinomas | T1bN0M0 | 29.61 | 40 |
| UD0272 | 49 | Female | Renal carcinomas | T1aN0M0 | 30.7 | 43 |
| UD0282 | 54 | Female | Renal carcinomas | T1bN0M0 | 31.53 | 43 |
| UD0283 | 60 | Male | Renal carcinomas | T1bN0M0 | 30.61 | 43 |
| UD0286 | 56 | Male | Renal carcinomas | T1aN0M0 | 32.14 | 43 |
| UD0295 | 49 | Female | Renal carcinomas | T2aN0M0 | 28.8 | 43 |
| UD0299 | 68 | Female | Renal carcinomas | T1aN0M0 | 28.25 | 40 |
| UD0302 | 53 | Female | Renal carcinomas | T1bN0M0 | 29.49 | 43 |
| UD0347 | 65 | Female | Renal carcinomas | T1aN0M0 | 28.45 | 39.28 |
| UD0365 | 61 | Female | Renal carcinomas | T1bN0M0 | 29.72 | 43 |
| UD0371 | 61 | Male | Renal carcinomas | T1bN0M0 | 29.13 | 40 |
| UA142 | 50 | Male | Carcinomas of renal pelvis | T4N0M0 | 29.07 | 35.83 |
| UA185 | 80 | Male | Carcinomas of renal pelvis | T3NXM0 | 28.12 | 34.16 |
| UD0023 | 71 | Male | Carcinomas of renal pelvis | T3N0M0 | 28.15 | 30.98 |
| UD0042 | 66 | Female | Carcinomas of renal pelvis | T4N0M0 | 28.16 | 34.46 |
| UD0062 | 54 | Male | Carcinomas of renal pelvis | T3N0M0 | 28.12 | 36.79 |
| UD0067 | 64 | Female | Carcinomas of renal pelvis | T1N0M0 | 28.24 | 37.28 |
| UD0079 | 60 | Male | Carcinomas of renal pelvis | T3N0M0 | 28.06 | 32.87 |
| UD0113 | 62 | Male | Carcinomas of renal pelvis | T2N0M0 | 28.22 | 30.13 |
| UD0146 | 69 | Male | Carcinomas of renal pelvis | T3N0M0 | 28.47 | 29.89 |
| UD0251 | 68 | Male | Carcinomas of renal pelvis | T3N2M0 | 28.29 | 32.56 |
| UD0253 | 82 | Female | Carcinomas of renal pelvis | T3N0M0 | 28.62 | 40 |
| UD0290 | 61 | Male | Carcinomas of renal pelvis | T3N0M0 | 28.14 | 37.3 |
| UD0384 | 64 | Male | Carcinomas of renal pelvis | T3N1M0 | 28.86 | 43 |
| UD0462 | 81 | Male | Carcinomas of renal pelvis | T3N0M0 | 28.43 | 29.65 |
| UD0494 | 71 | Female | Carcinomas of renal pelvis | T3N0M0 | 28.26 | 32.91 |
| UD0532 | 78 | Male | Carcinomas of renal pelvis | T2N0M0 | 28.58 | 31.33 |
| UD0632 | 78 | Male | Carcinomas of renal pelvis | T1aN0M0 | 28.23 | 32.77 |
| UD0724 | 78 | Female | Carcinomas of renal pelvis | T1N0M0 | 28.27 | 30.84 |
| UD0727 | 65 | Male | Carcinomas of renal pelvis | T1N0M0 | 29.13 | 43 |
| UD0728 | 52 | Female | Carcinomas of renal pelvis | T3N0M0 | 28.93 | 35.68 |
| UD0729 | 57 | Male | Carcinomas of renal pelvis | T2N0M0 | 28.51 | 32.77 |
| UD0737 | 79 | Male | Carcinomas of renal pelvis | T3N2M | 28.75 | 29.96 |
| UG048 | 52 | Female | Carcinomas of renal pelvis | T3aN1M0 | 28.26 | 36.65 |
| UD0075 | 49 | Male | carcinomas of ureter | T2N0M0 | 34.66 | 43 |
| UD0156 | 63 | Male | carcinomas of ureter | T2N0M0 | 28.06 | 43 |
| UD0177 | 69 | Male | carcinomas of ureter | T2N0M0 | 27.99 | 29.11 |
| UD0246 | 65 | Male | carcinomas of ureter | T4N0M0 | 28.02 | 31.12 |
| UD0414 | 68 | Female | carcinomas of ureter | T1N0M0 | 28.31 | 30.18 |
| UD0526 | 59 | Female | carcinomas of ureter | T2N2M0 | 28.05 | 32.36 |
| UD0593 | 70 | Male | carcinomas of ureter | TaN0M0 | 28.33 | 40 |
| UD0635 | 61 | Male | carcinomas of ureter | T2N0M0 | 33.65 | 43 |
| UD0664 | 57 | Male | carcinomas of ureter | TaN0M0 | 28.21 | 37.04 |
| UD0806 | 66 | Female | carcinomas of ureter | T1N0M0 | 28.25 | 35.82 |
| UD0820 | 66 | Male | carcinomas of ureter | T2N0M0 | 27.83 | 40 |
| UG078 | 60 | Male | carcinomas of ureter | T1N0M0 | 29.19 | 34.65 |
| UG085 | 55 | Male | carcinomas of ureter | T2aN0M0 | 28.27 | 32.66 |
| UA161 | 45 | Male | Benign tumors of the bladder | NA | 28.3 | 38.43 |
| UA183 | 66 | Female | Benign tumors of the bladder | NA | 28.29 | 40 |
| UD0007 | 58 | Male | Benign tumors of the bladder | NA | 28.25 | 40 |
| UD0048 | 42 | Male | Benign tumors of the bladder | NA | 30.51 | 43 |
| UD0077 | 59 | Male | Benign tumors of the bladder | NA | 34.47 | 43 |
| UD0087 | 72 | Male | Benign tumors of the bladder | NA | 28.01 | 43 |
| UD0091 | 48 | Male | Benign tumors of the bladder | NA | 29.12 | 43 |
| UD0160 | 80 | Female | Benign tumors of the bladder | NA | 30.1 | 37.03 |
| UD0181 | 60 | Male | Benign tumors of the bladder | NA | 28.15 | 40 |
| UD0230 | 42 | Male | Benign tumors of the bladder | NA | 28.24 | 43 |
| UD0319 | 46 | Male | Benign tumors of the bladder | NA | 29.92 | 40 |
| UD0335 | 56 | Male | Benign tumors of the bladder | NA | 29.1 | 40 |
| UD0589 | 68 | Male | Benign tumors of the bladder | NA | 31.29 | 43 |
| UD0594 | 54 | Male | Benign tumors of the bladder | NA | 28.94 | 34.78 |
| UD0628 | 70 | Male | Benign tumors of the bladder | NA | 27.83 | 36.51 |
| UD0654 | 48 | Male | Benign tumors of the bladder | NA | 28.82 | 40 |
| UD0665 | 63 | Male | Benign tumors of the bladder | NA | 28.47 | 43 |
| UD0689 | 77 | Female | Benign tumors of the bladder | NA | 30.45 | 43 |
| UD0691 | 65 | Male | Benign tumors of the bladder | NA | 28.28 | 43 |
| UD0708 | 59 | Male | Benign tumors of the bladder | NA | 29.7 | 32.5 |
| UD0740 | 62 | Male | Benign tumors of the bladder | NA | 31.33 | 43 |
| UD0775 | 76 | Male | Benign tumors of the bladder | NA | 30.09 | 40 |
| UD0794 | 52 | Female | Benign tumors of the bladder | NA | 28.11 | 37.28 |
| UD0824 | 62 | Male | Benign tumors of the bladder | NA | 32.66 | 43 |
| UG010 | 66 | Male | Benign tumors of the bladder | NA | 32.06 | 43 |
| UG043 | 85 | Male | Benign tumors of the bladder | NA | 28.31 | 39.47 |
| UG052 | 61 | Female | Benign tumors of the bladder | NA | 28.08 | 37.36 |
| UG098 | 53 | Male | Benign tumors of the bladder | NA | 28.79 | 43 |
| UA054 | 71 | Male | prostate cancers | pMIc | 28.28 | 40 |
| UA176 | 75 | Male | prostate cancers | pMIc | 32.53 | 43 |
| UA177 | 71 | Male | prostate cancers | pMIc | 28.76 | 37.44 |
| UA180 | 64 | Male | prostate cancers | pMIc | 28.8 | 40 |
| UA184 | 75 | Male | prostate cancers | T3bN0M0 | 30.07 | 37.07 |
| UD0024 | 64 | Male | prostate cancers | T3bN0M0 | 28.53 | 33 |
| UD0158 | 47 | Male | prostate cancers | TxN1M0 | 31.17 | 43 |
| UD0245 | 80 | Male | prostate cancers | T3bN0M0 | 30.62 | 43 |
| UD0247 | 61 | Male | prostate cancers | T2cN1M0 | 32.23 | 40 |
| UD0254 | 79 | Male | prostate cancers | T2cN0M0 | 28.18 | 43 |
| UD0255 | 68 | Male | prostate cancers | T3bN1M0 | 30.04 | 43 |
| UD0260 | 81 | Male | prostate cancers | T3bN0M0 | 34.28 | 43 |
| UD0261 | 59 | Male | prostate cancers | T4NxM0 | 32.78 | 43 |
| UD0017 | 68 | Male | Postoperative patients（Recurring cancers） | T4aN2M0 | 28.21 | 40 |
| UD0020 | 66 | Male | Postoperative patients（Recurring cancers） | T1N0M0 | 28.07 | 32.31 |
| UD0026 | 71 | Male | Postoperative patients（Recurring cancers） | T2N0M0 | 28.16 | 37.73 |
| UD0080 | 69 | Male | Postoperative patients（Recurring cancers） | T1N0M0 | 28.73 | 29.04 |
| UD0085 | 60 | Male | Postoperative patients（Recurring cancers） | T1N0M0 | 28.5 | 31.81 |
| UD0094 | 74 | Male | Postoperative patients（Recurring cancers） | NA | 28.41 | 33.51 |
| UD0098 | 61 | Male | Postoperative patients（Recurring cancers） | NA | 30.49 | 31.79 |
| UD0103 | 53 | Male | Postoperative patients（Recurring cancers） | T2N0M0 | 28.23 | 32.24 |
| UD0243 | 67 | Male | Postoperative patients（Recurring cancers） | T1N0M0 | 29.53 | 34.75 |
| UD0267 | 60 | Male | Postoperative patients（Recurring cancers） | T1N0M0 | 28.16 | 31.31 |
| UD0268 | 65 | Male | Postoperative patients（Recurring cancers） | T1N0M0 | 28.5 | 29.92 |
| UD0288 | 81 | Male | Postoperative patients（Recurring cancers） | T1N0M0 | 28.62 | 30.2 |
| UD0312 | 57 | Male | Postoperative patients（Recurring cancers） | T1N0M0 | 28.34 | 37.28 |
| UD0361 | 50 | Female | Postoperative patients（Recurring cancers） | TaN0M0 | 28.47 | 35.08 |
| UD0374 | 74 | Male | Postoperative patients（Recurring cancers） | T1N0M0 | 28.32 | 30.85 |
| UD0463 | 59 | Male | Postoperative patients（Recurring cancers） | T4aN3M0 | 28.44 | 34.28 |
| UD0559 | 49 | Female | Postoperative patients（Recurring cancers） | T1N0M0 | 28.55 | 29.86 |
| UD0560 | 82 | Male | Postoperative patients（Recurring cancers） | T3N0M0 | 27.99 | 34.15 |
| UD0634 | 71 | Male | Postoperative patients（Recurring cancers） | TaN0M0 | 28.85 | 34.93 |
| UD0638 | 62 | Female | Postoperative patients（Recurring cancers） | TaN0M0 | 29.64 | 40 |
| UD0838 | 80 | Male | Postoperative patients（Recurring cancers） | T1N0M0 | 28.47 | 43 |
| UG077 | 44 | Male | Postoperative patients（Recurring cancers） | T1N0M0 | 28.6 | 31.35 |
| UD0097 | 56 | Male | Postoperative patients（healthy） | TaN2M0 | 28.1 | 38.33 |
| UD0585 | 57 | Female | Postoperative patients（healthy） | NA | 27.93 | 36.42 |
| UG016 | 59 | Male | Postoperative patients（healthy） | NA | 28.24 | 37.94 |
| UG022 | 60 | Male | Postoperative patients（healthy） | NA | 28.14 | 36.9 |
| UD0022 | 63 | Male | Postoperative patients（healthy） | TaN0M0 | 28.09 | 43 |
| UD0143 | 63 | Female | Postoperative patients（healthy） | NA | 29.01 | 43 |
| UD0429 | 66 | Male | Postoperative patients（healthy） | NA | 28.53 | 43 |
| UD0239 | 82 | Male | Postoperative patients（healthy） | NA | 28.11 | 30.36 |

*****Reference gene is *ACTB*.

**Supplementary Table S9. Clinical features of the validation cohort**

|  | **BC *(n* = 79)** | ***Non-BC (interfering cancers (*n* = 107))** | **benign tumors of bladder (*n* = 22)** | **postoperative (*n* = 8)** | | **Normal (*n* = 304)** |
| --- | --- | --- | --- | --- | --- | --- |
|  |  |  |  | healthy (*n* = 0) | recurring cancers (*n* = 8) |  |
| **Age, years** |  |  |  |  | |  |
| Median (range) | 69 (27–92) | 66 (41–85) | 58 (25–79) | NA | 68 (63–74) | 60 (38–89) |
| <60 | 17 (21.5%) | 29 (27.1%) | 12 (54.5%) | NA | 0 (0%) | 148 (21.5%) |
| ≥60 | 62 (78.5%) | 78 (72.9%) | 10 (45.5%) | NA | 8 (100%) | 156 |
| **Sex, n (%)** |  |  |  |  |  |  |
| Male | 62 (78.4%) | 83 (77.5%) | 19 (86.4%) | NA | 7 (87.5%) | 173 (56.9%) |
| Female | 17 (21.6%) | 24 (22.5%) | 3 (13.6%) | NA | 1 (12.5%) | 131 (43.1%) |
| **Stage, n (%)** |  |  |  | NA |  |  |
| Ta/T1 | 43 (54.4%) | NA | NA | NA | 1 (12.5%) | NA |
| T2/T3/T4 | 18 (22.8%) | NA | NA | NA | 1 (12.5%) | NA |
| NA | 18 (22.8%) | NA | NA | NA | 6 (75.0%) | NA |
| **Grade, n (%)** |  |  | NA |  |  |  |
| low | 37 (46.9%) | NA | NA | NA | 0 (0%) | NA |
| High | 34 (43.0%) | NA | NA | NA | 7 (87.5%) | NA |
| NA | 8 (10.1%) | NA | NA | NA | 1 (12.5%) | NA |
| *Non-BC (interfering cancers (*n* = 107)): carcinomas of renal pelvis (*n* = 6) carcinomas of ureter (*n* = 12), renal carcinomas (*n* = 40), prostate cancers (*n* = 49) | | | | | | |

**Supplementary Table S10. CT values obtained from qMSP of *DMRTA2* for urine samples of the validation set**

| **Sample ID** | **Age** | **Sex** | **Group** | **TNM** | **CT.*ACTB**** | **CT.*DMRTA2*** |
| --- | --- | --- | --- | --- | --- | --- |
| UC296 | 69 | Male | Bladder cancer | NA | 28.67 | 43 |
| UC082 | 50 | Female | Bladder cancer | T2bN0M0 | 28.79 | 43 |
| UC078 | 35 | Female | Bladder cancer | TaN0M0 | 31.18 | 43 |
| UD1091 | 62 | Female | Bladder cancer | T1N0M0 | 31.61 | 43 |
| UA248 | 65 | Male | Bladder cancer | TaN0M0 | 29.77 | 40 |
| UC096 | 54 | Male | Bladder cancer | TaN0M0 | 30.22 | 40 |
| UC069 | 84 | Male | Bladder cancer | TaN0M0 | 30.44 | 40 |
| UA275 | 73 | Male | Bladder cancer | TaN0M0 | 27.9 | 39.99 |
| UC315 | 70 | Male | Bladder cancer | NA | 28.57 | 39.09 |
| UD1111 | 57 | Male | Bladder cancer | T1N0M0 | 28.59 | 38.96 |
| UA280 | 68 | Male | Bladder cancer | TaN0M0 | 27.86 | 38.7 |
| UC121 | 85 | Male | Bladder cancer | NA | 28.21 | 38.36 |
| UA307 | 36 | Male | Bladder cancer | TaN0M0 | 27.8 | 37.83 |
| UC092 | 54 | Female | Bladder cancer | TaN0M0 | 28.25 | 37.66 |
| UC062 | 59 | Male | Bladder cancer | T1N0M0 | 28.55 | 37.2 |
| UD0891 | 85 | Male | Bladder cancer | T1N0M0 | 28.12 | 37.12 |
| UA293 | 75 | Male | Bladder cancer | TaN0M0 | 30.98 | 37.11 |
| UC045 | 77 | Male | Bladder cancer | T2N0M0 | 28.18 | 36.79 |
| UC018 | 66 | Male | Bladder cancer | NA | 28.07 | 36.77 |
| UA238 | 73 | Male | Bladder cancer | TaN0M0 | 29.46 | 36.54 |
| UC068 | 54 | Male | Bladder cancer | T1N0M0 | 29.81 | 36.42 |
| UA306 | 81 | Male | Bladder cancer | T2N0M0 | 28.14 | 36.2 |
| UA243 | 65 | Male | Bladder cancer | TaN0M0 | 27.95 | 36.16 |
| UA278 | 58 | Male | Bladder cancer | TaN0M0 | 27.96 | 35.82 |
| UC387 | 73 | Male | Bladder cancer | NA | 29.97 | 35.82 |
| UD0912 | 71 | Female | Bladder cancer | T2bN0M0 | 29.1 | 35.76 |
| UD1034 | 62 | Male | Bladder cancer | T2N0M0 | 28.65 | 35.63 |
| UC083 | 57 | Male | Bladder cancer | TaN0M0 | 29.71 | 35.51 |
| UD0210 | 63 | Male | Bladder cancer | T1N0M0 | 28.48 | 34.8 |
| UD0084 | 27 | Female | Bladder cancer | T4N1M0 | 28.03 | 34.59 |
| UC370 | 84 | Male | Bladder cancer | NA | 28.75 | 34.46 |
| UD1011 | 66 | Male | Bladder cancer | T1N0M0 | 28.82 | 34.17 |
| UD1088 | 69 | Male | Bladder cancer | T1N0M0 | 29.23 | 33.94 |
| UA004 | 73 | Male | Bladder cancer | T1N0M0 | 30.42 | 33.92 |
| UC135 | 88 | Male | Bladder cancer | NA | 28.17 | 33.83 |
| UD1045 | 76 | Male | Bladder cancer | T3N1M0 | 28.59 | 33.83 |
| UC188 | 65 | Male | Bladder cancer | NA | 28.75 | 33.43 |
| UC401 | 79 | Male | Bladder cancer | NA | 28.84 | 33.25 |
| UD1057 | 60 | Female | Bladder cancer | T4N0M0 | 28.56 | 33.19 |
| UD1080 | 53 | Male | Bladder cancer | T1N0M0 | 32.49 | 33.06 |
| UC280 | 81 | Male | Bladder cancer | NA | 28.61 | 32.94 |
| UD1106 | 55 | Female | Bladder cancer | T4N1M0 | 28.58 | 32.92 |
| UD1146 | 73 | Male | Bladder cancer | T1N0M0 | 28.41 | 32.83 |
| UC070 | 66 | Male | Bladder cancer | TaN0M0 | 27.85 | 32.67 |
| UD1118 | 43 | Male | Bladder cancer | T1N0M0 | 28.89 | 32.61 |
| UC350 | 87 | Male | Bladder cancer | NA | 28.77 | 32.48 |
| UC405 | 88 | Female | Bladder cancer | NA | 28.69 | 32.45 |
| UD0922 | 88 | Female | Bladder cancer | T1N0M0 | 28.99 | 32.31 |
| UC087 | 44 | Female | Bladder cancer | TaN0M0 | 28.64 | 31.68 |
| UA218 | 53 | Male | Bladder cancer | T3bN0M0 | 28.46 | 31.64 |
| UA141 | 74 | Male | Bladder cancer | TaN0M0 | 28.28 | 31.25 |
| UD0961 | 77 | Male | Bladder cancer | T2N0M0 | 28.22 | 30.96 |
| UC156 | 68 | Male | Bladder cancer | NA | 29.42 | 30.9 |
| UD0060 | 74 | Male | Bladder cancer | NA | 28.06 | 30.88 |
| UD0925 | 76 | Male | Bladder cancer | T1N0M0 | 28.55 | 30.65 |
| UC101 | 75 | Female | Bladder cancer | TaN0M0 | 28.43 | 30.6 |
| UD1149 | 65 | Male | Bladder cancer | T1N0M0 | 28.6 | 30.5 |
| UD1015 | 81 | Female | Bladder cancer | T2bN0M0 | 29.63 | 30.29 |
| UD0941 | 64 | Female | Bladder cancer | T2aN0M0 | 28.43 | 30.15 |
| UA305 | 69 | Male | Bladder cancer | T1N0M0 | 28.56 | 30.11 |
| UC297 | 84 | Female | Bladder cancer | NA | 28.77 | 29.96 |
| UA295 | 81 | Male | Bladder cancer | T2N0M1 | 28.42 | 29.86 |
| UD1083 | 72 | Male | Bladder cancer | T1N0M0 | 28.55 | 29.81 |
| UC108 | 61 | Male | Bladder cancer | TaN0M0 | 28.04 | 29.8 |
| UC288 | 71 | Male | Bladder cancer | NA | 28.76 | 29.79 |
| UD0004 | 62 | Male | Bladder cancer | NA | 28.24 | 29.66 |
| UD0965 | 65 | Male | Bladder cancer | T2N0M0 | 28.44 | 29.58 |
| UD0995 | 75 | Male | Bladder cancer | T1N0M0 | 28.18 | 29.55 |
| UA213 | 80 | Male | Bladder cancer | TaN0M0 | 28.55 | 29.51 |
| UA255 | 77 | Female | Bladder cancer | T1N0M0 | 28.57 | 29.51 |
| UD1007 | 63 | Male | Bladder cancer | T2aN0M0 | 28.87 | 29.44 |
| UD1071 | 72 | Male | Bladder cancer | T2N1M0 | 28.11 | 29.37 |
| UC095 | 61 | Male | Bladder cancer | T1N0M0 | 28.01 | 29.34 |
| UD0892 | 92 | Female | Bladder cancer | T1N0M0 | 28.44 | 29.2 |
| UA303 | 79 | Male | Bladder cancer | TaN0M0 | 27.73 | 29.17 |
| UC035 | 77 | Male | Bladder cancer | T1N1M0 | 29.07 | 29.13 |
| UD1086 | 51 | Male | Bladder cancer | T4N0M0 | 27.87 | 28.98 |
| UC080 | 77 | Male | Bladder cancer | NA | 27.91 | 28.9 |
| UC107 | 67 | Male | Bladder cancer | TaN0M0 | 28.35 | 28.89 |
| UD0910 | 73 | Female | Control |  | 27.68 | 38.15 |
| UA262 | 77 | Male | Control |  | 27.71 | 43 |
| UD0883 | 66 | Male | Control |  | 27.71 | 35.91 |
| UD0935 | 58 | Female | Control |  | 27.72 | 37.69 |
| UD0928 | 50 | Male | Control |  | 27.79 | 43 |
| UD0895 | 56 | Female | Control |  | 27.79 | 39.49 |
| UA281 | 77 | Male | Control |  | 27.8 | 43 |
| UC248 | 58 | Female | Control |  | 27.81 | 43 |
| UD0859 | 60 | Female | Control |  | 27.82 | 38.05 |
| UD0904 | 53 | Male | Control |  | 27.84 | 43 |
| UD0938 | 76 | Female | Control |  | 27.84 | 37.18 |
| UD0887 | 53 | Male | Control |  | 27.84 | 33.92 |
| UA247 | 72 | Male | Control |  | 27.86 | 43 |
| UD0899 | 78 | Male | Control |  | 27.87 | 37.67 |
| UD0937 | 71 | Female | Control |  | 27.88 | 40.11 |
| UA217 | 70 | Male | Control |  | 27.94 | 40 |
| UA254 | 63 | Male | Control |  | 27.94 | 39.4 |
| UD0939 | 68 | Male | Control |  | 27.95 | 39.48 |
| UA311 | 40 | Male | Control |  | 27.97 | 43 |
| UD0911 | 52 | Female | Control |  | 27.97 | 39.63 |
| UD0873 | 66 | Male | Control |  | 27.97 | 37.87 |
| UD0861 | 52 | Male | Control |  | 27.98 | 37.02 |
| UD0856 | 65 | Female | Control |  | 27.99 | 43 |
| UD1087 | 57 | Female | Control |  | 28 | 38.06 |
| UA274 | 65 | Male | Control |  | 28.01 | 43 |
| UD0996 | 67 | Male | Control |  | 28.02 | 43 |
| UA269 | 74 | Male | Control |  | 28.02 | 39.72 |
| UA241 | 68 | Male | Control |  | 28.04 | 43 |
| UA240 | 67 | Male | Control |  | 28.04 | 40 |
| UA261 | 61 | Male | Control |  | 28.04 | 35.7 |
| UD0561 | 51 | Female | Control |  | 28.06 | 40 |
| UC019 | 56 | Female | Control |  | 28.1 | 38.3 |
| UA313 | 60 | Male | Control |  | 28.14 | 40.34 |
| UC001 | 52 | Male | Control |  | 28.14 | 35.77 |
| UC098 | 52 | Male | Control |  | 28.15 | 40 |
| UA223 | 80 | Male | Control |  | 28.16 | 39.03 |
| UA224 | 64 | Male | Control |  | 28.16 | 38.58 |
| UC146 | 41 | Male | Control |  | 28.16 | 38.08 |
| UD0927 | 76 | Male | Control |  | 28.17 | 40.33 |
| UA304 | 47 | Female | Control |  | 28.17 | 40 |
| UD1101 | 66 | Male | Control |  | 28.17 | 40 |
| UA221 | 73 | Male | Control |  | 28.18 | 43 |
| UC017 | 46 | Female | Control |  | 28.18 | 40 |
| UC133 | 61 | Male | Control |  | 28.18 | 39.98 |
| UC132 | 62 | Female | Control |  | 28.19 | 43 |
| UC020 | 43 | Male | Control |  | 28.19 | 40 |
| UA258 | 48 | Male | Control |  | 28.19 | 37.36 |
| UA298 | 61 | Male | Control |  | 28.2 | 43 |
| UC044 | 59 | Male | Control |  | 28.2 | 40 |
| UC376 | 69 | Male | Control |  | 28.2 | 40 |
| UD0513 | 58 | Female | Control |  | 28.21 | 43 |
| UC125 | 55 | Male | Control |  | 28.21 | 43 |
| UC118 | 77 | Male | Control |  | 28.21 | 31.65 |
| UC117 | 50 | Male | Control |  | 28.22 | 43 |
| UD0549 | 56 | Female | Control |  | 28.22 | 38.36 |
| UA263 | 47 | Male | Control |  | 28.22 | 37.83 |
| UC123 | 52 | Male | Control |  | 28.23 | 43 |
| UC139 | 54 | Male | Control |  | 28.24 | 40 |
| UD0169 | 42 | Male | Control |  | 28.24 | 37.7 |
| UC263 | 66 | Male | Control |  | 28.24 | 37.03 |
| UD0870 | 52 | Female | Control |  | 28.25 | 43 |
| UA260 | 44 | Male | Control |  | 28.25 | 37.04 |
| UD0214 | 74 | Male | Control |  | 28.26 | 43 |
| UA207 | 78 | Male | Control |  | 28.26 | 40 |
| UC134 | 55 | Male | Control |  | 28.27 | 40 |
| UD0510 | 58 | Female | Control |  | 28.27 | 39.04 |
| UD0915 | 57 | Female | Control |  | 28.28 | 39.12 |
| UD1104 | 75 | Male | Control |  | 28.28 | 37.22 |
| UC252 | 55 | Female | Control |  | 28.28 | 36.83 |
| UC126 | 62 | Male | Control |  | 28.29 | 40 |
| UC030 | 62 | Female | Control |  | 28.29 | 40 |
| UD0908 | 81 | Male | Control |  | 28.29 | 35.51 |
| UC202 | 55 | Female | Control |  | 28.29 | 35.42 |
| UC115 | 75 | Male | Control |  | 28.3 | 43 |
| UC137 | 55 | Male | Control |  | 28.3 | 43 |
| UD0872 | 63 | Female | Control |  | 28.3 | 39.42 |
| UC047 | 58 | Female | Control |  | 28.31 | 38.82 |
| UC120 | 63 | Male | Control |  | 28.32 | 40 |
| UC007 | 42 | Female | Control |  | 28.32 | 40 |
| UA315 | 63 | Female | Control |  | 28.33 | 43 |
| UD0942 | 63 | Male | Control |  | 28.33 | 43 |
| UC033 | 45 | Female | Control |  | 28.34 | 40 |
| UD0868 | 65 | Male | Control |  | 28.41 | 40.1 |
| UD0951 | 69 | Male | Control |  | 28.41 | 39.26 |
| UC337 | 57 | Male | Control |  | 28.41 | 37.46 |
| UC114 | 54 | Male | Control |  | 28.42 | 38.19 |
| UC240 | 69 | Female | Control |  | 28.44 | 43 |
| UC392 | 46 | Male | Control |  | 28.44 | 43 |
| UC236 | 67 | Female | Control |  | 28.44 | 40 |
| UC149 | 75 | Male | Control |  | 28.44 | 39.47 |
| UD1097 | 57 | Male | Control |  | 28.45 | 40 |
| UA186 | 66 | Male | Control |  | 28.45 | 39.09 |
| UC483 | 42 | Male | Control |  | 28.45 | 38.06 |
| UA172 | 45 | Female | Control |  | 28.46 | 43 |
| UA215 | 73 | Male | Control |  | 28.46 | 43 |
| UD0583 | 51 | Female | Control |  | 28.46 | 40 |
| UC155 | 44 | Male | Control |  | 28.46 | 40 |
| UD1056 | 65 | Male | Control |  | 28.46 | 40 |
| UC145 | 67 | Male | Control |  | 28.46 | 39.09 |
| UC088 | 81 | Male | Control |  | 28.46 | 38.08 |
| UA259 | 80 | Male | Control |  | 28.47 | 43 |
| UD0980 | 62 | Male | Control |  | 28.47 | 43 |
| UC113 | 65 | Male | Control |  | 28.47 | 40 |
| UC152 | 52 | Female | Control |  | 28.47 | 40 |
| UC147 | 69 | Female | Control |  | 28.47 | 38.9 |
| UC451 | 60 | Female | Control |  | 28.47 | 38.21 |
| UD0966 | 59 | Female | Control |  | 28.48 | 40 |
| UC507 | 50 | Female | Control |  | 28.48 | 37.07 |
| UA312 | 55 | Female | Control |  | 28.49 | 37.65 |
| UC422 | 59 | Male | Control |  | 28.5 | 40 |
| UD0841 | 77 | Female | Control |  | 28.5 | 39.51 |
| UC399 | 66 | Female | Control |  | 28.5 | 37.3 |
| UD1053 | 65 | Female | Control |  | 28.5 | 37.23 |
| UC186 | 55 | Male | Control |  | 28.5 | 36.99 |
| UD0764 | 46 | Male | Control |  | 28.51 | 43 |
| UA282 | 74 | Male | Control |  | 28.51 | 43 |
| UD0983 | 40 | Male | Control |  | 28.51 | 43 |
| UC430 | 63 | Male | Control |  | 28.51 | 40 |
| UC442 | 68 | Male | Control |  | 28.51 | 40 |
| UD1039 | 63 | Male | Control |  | 28.51 | 39.22 |
| UD0783 | 54 | Female | Control |  | 28.52 | 40 |
| UC081 | 69 | Male | Control |  | 28.52 | 40 |
| UD0948 | 82 | Male | Control |  | 28.53 | 40 |
| UC097 | 68 | Female | Control |  | 28.54 | 38.22 |
| UD1001 | 65 | Male | Control |  | 28.55 | 43 |
| UC395 | 74 | Female | Control |  | 28.55 | 43 |
| UD0985 | 53 | Male | Control |  | 28.55 | 40 |
| UC024 | 45 | Female | Control |  | 28.55 | 40 |
| UD0558 | 56 | Male | Control |  | 28.56 | 43 |
| UD0879 | 65 | Male | Control |  | 28.56 | 43 |
| UC239 | 56 | Male | Control |  | 28.56 | 36.92 |
| UD0953 | 53 | Female | Control |  | 28.58 | 43 |
| UD0344 | 67 | Female | Control |  | 28.59 | 43 |
| UD0604 | 59 | Female | Control |  | 28.59 | 40 |
| UC386 | 69 | Male | Control |  | 28.59 | 38.19 |
| UD0986 | 75 | Male | Control |  | 28.6 | 39.68 |
| UC032 | 73 | Male | Control |  | 28.6 | 36.81 |
| UA245 | 77 | Male | Control |  | 28.6 | 36.64 |
| UD0704 | 66 | Female | Control |  | 28.61 | 43 |
| UC398 | 57 | Male | Control |  | 28.61 | 38.45 |
| UD0483 | 53 | Female | Control |  | 28.62 | 40 |
| UC268 | 72 | Male | Control |  | 28.62 | 40 |
| UC373 | 80 | Male | Control |  | 28.62 | 38.85 |
| UC207 | 61 | Female | Control |  | 28.62 | 29.76 |
| UC013 | 43 | Male | Control |  | 28.63 | 40 |
| UC406 | 44 | Male | Control |  | 28.63 | 40 |
| UC461 | 55 | Female | Control |  | 28.63 | 40 |
| UC303 | 57 | Female | Control |  | 28.63 | 36.66 |
| UD0791 | 55 | Female | Control |  | 28.64 | 43 |
| UC046 | 61 | Male | Control |  | 28.64 | 35.31 |
| UA257 | 53 | Female | Control |  | 28.65 | 43 |
| UD0978 | 60 | Female | Control |  | 28.65 | 43 |
| UC460 | 57 | Female | Control |  | 28.65 | 38.19 |
| UC466 | 58 | Male | Control |  | 28.66 | 40 |
| UD1119 | 69 | Male | Control |  | 28.67 | 43 |
| UC474 | 57 | Female | Control |  | 28.67 | 37.76 |
| UD1059 | 63 | Male | Control |  | 28.68 | 43 |
| UD0660 | 57 | Female | Control |  | 28.68 | 43 |
| UD1044 | 75 | Male | Control |  | 28.68 | 37.41 |
| UC178 | 55 | Male | Control |  | 28.68 | 36.93 |
| UC284 | 66 | Female | Control |  | 28.69 | 43 |
| UA216 | 81 | Male | Control |  | 28.69 | 43 |
| UC270 | 60 | Male | Control |  | 28.69 | 39.28 |
| UC253 | 65 | Female | Control |  | 28.69 | 39.11 |
| UC385 | 86 | Male | Control |  | 28.69 | 38.19 |
| UC022 | 65 | Female | Control |  | 28.7 | 43 |
| UC198 | 57 | Male | Control |  | 28.7 | 40 |
| UA266 | 69 | Male | Control |  | 28.7 | 39.51 |
| UC459 | 38 | Female | Control |  | 28.7 | 39.47 |
| UD1079 | 71 | Female | Control |  | 28.7 | 38.14 |
| UC250 | 73 | Female | Control |  | 28.7 | 37.9 |
| UC463 | 55 | Female | Control |  | 28.71 | 43 |
| UA206 | 73 | Male | Control |  | 28.71 | 43 |
| UC154 | 44 | Female | Control |  | 28.73 | 40 |
| UD1032 | 62 | Male | Control |  | 28.74 | 43 |
| UD1142 | 71 | Male | Control |  | 28.75 | 43 |
| UD0464 | 55 | Male | Control |  | 28.75 | 43 |
| UC040 | 47 | Female | Control |  | 28.76 | 39.25 |
| UD1107 | 70 | Female | Control |  | 28.76 | 37.16 |
| UD1023 | 63 | Female | Control |  | 28.77 | 37.33 |
| UD0610 | 48 | Female | Control |  | 28.78 | 43 |
| UC059 | 73 | Male | Control |  | 28.79 | 40 |
| UD1081 | 59 | Female | Control |  | 28.8 | 40 |
| UC230 | 59 | Female | Control |  | 28.8 | 40 |
| UC490 | 69 | Female | Control |  | 28.81 | 36.52 |
| UD0452 | 59 | Female | Control |  | 28.82 | 40 |
| UD1151 | 62 | Male | Control |  | 28.83 | 43 |
| UC456 | 69 | Male | Control |  | 28.84 | 43 |
| UD1055 | 58 | Female | Control |  | 28.85 | 43 |
| UD0741 | 46 | Female | Control |  | 28.85 | 43 |
| UC258 | 63 | Female | Control |  | 28.86 | 35.97 |
| UD0876 | 42 | Female | Control |  | 28.87 | 40.92 |
| UC330 | 53 | Female | Control |  | 28.87 | 40 |
| UC496 | 69 | Male | Control |  | 28.87 | 34.58 |
| UD1006 | 62 | Male | Control |  | 28.9 | 43 |
| UD0926 | 46 | Female | Control |  | 28.91 | 40.5 |
| UC201 | 69 | Male | Control |  | 28.91 | 37.93 |
| UD0974 | 60 | Female | Control |  | 28.92 | 43 |
| UC194 | 65 | Female | Control |  | 28.93 | 37.68 |
| UA204 | 64 | Female | Control |  | 28.94 | 40 |
| UC031 | 49 | Male | Control |  | 28.96 | 43 |
| UA237 | 83 | Male | Control |  | 28.96 | 40 |
| UC259 | 54 | Female | Control |  | 28.96 | 40 |
| UD1051 | 72 | Female | Control |  | 28.97 | 43 |
| UA314 | 52 | Female | Control |  | 28.97 | 39.75 |
| UD1090 | 72 | Male | Control |  | 28.98 | 43 |
| UA317 | 54 | Male | Control |  | 29 | 39.25 |
| UG027 | 57 | Male | Control |  | 29.01 | 43 |
| UA319 | 56 | Female | Control |  | 29.01 | 39.55 |
| UC351 | 59 | Female | Control |  | 29.02 | 36.81 |
| UD0280 | 53 | Female | Control |  | 29.05 | 40 |
| UD0616 | 41 | Male | Control |  | 29.07 | 40 |
| UD1000 | 44 | Female | Control |  | 29.07 | 39.48 |
| UC378 | 64 | Female | Control |  | 29.09 | 43 |
| UD0917 | 61 | Male | Control |  | 29.12 | 39.86 |
| UD0644 | 65 | Female | Control |  | 29.13 | 40 |
| UD0987 | 57 | Male | Control |  | 29.15 | 43 |
| UD0163 | 41 | Female | Control |  | 29.17 | 43 |
| UG021 | 45 | Female | Control |  | 29.17 | 38.49 |
| UC005 | 42 | Female | Control |  | 29.19 | 36.95 |
| UC415 | 54 | Female | Control |  | 29.26 | 43 |
| UD0623 | 55 | Female | Control |  | 29.33 | 43 |
| UC200 | 55 | Female | Control |  | 29.33 | 38.1 |
| UC065 | 43 | Female | Control |  | 29.41 | 40 |
| UD1089 | 65 | Male | Control |  | 29.41 | 40 |
| UD0403 | 54 | Male | Control |  | 29.47 | 43 |
| UC140 | 61 | Male | Control |  | 29.47 | 39.16 |
| UA244 | 68 | Male | Control |  | 29.49 | 43 |
| UD0418 | 59 | Female | Control |  | 29.53 | 40 |
| UD0633 | 69 | Female | Control |  | 29.54 | 40 |
| UC006 | 60 | Female | Control |  | 29.55 | 43 |
| UD0362 | 52 | Male | Control |  | 29.62 | 40 |
| UC158 | 72 | Female | Control |  | 29.64 | 40 |
| UC246 | 65 | Male | Control |  | 29.66 | 40 |
| UD0442 | 48 | Female | Control |  | 29.71 | 40 |
| UD0880 | 51 | Male | Control |  | 29.81 | 43 |
| UD0460 | 53 | Male | Control |  | 29.89 | 43 |
| UD0884 | 66 | Male | Control |  | 29.91 | 43 |
| UD0882 | 57 | Male | Control |  | 29.93 | 43 |
| UC051 | 63 | Male | Control |  | 29.97 | 43 |
| UD0973 | 43 | Female | Control |  | 29.98 | 39.14 |
| UG009 | 52 | Male | Control |  | 29.99 | 43 |
| UD0605 | 75 | Female | Control |  | 29.99 | 33.63 |
| UD0376 | 47 | Female | Control |  | 30 | 40 |
| UD0901 | 62 | Female | Control |  | 30.07 | 43 |
| UD0957 | 54 | Male | Control |  | 30.08 | 43 |
| UC404 | 55 | Male | Control |  | 30.1 | 43 |
| UC271 | 69 | Female | Control |  | 30.18 | 37.88 |
| UA203 | 79 | Male | Control |  | 30.19 | 43 |
| UD0520 | 65 | Male | Control |  | 30.22 | 43 |
| UD0485 | 61 | Male | Control |  | 30.24 | 40 |
| UD0962 | 66 | Female | Control |  | 30.26 | 40 |
| UA284 | 70 | Male | Control |  | 30.31 | 43 |
| UC374 | 53 | Female | Control |  | 30.31 | 37.29 |
| UD0885 | 61 | Male | Control |  | 30.34 | 37.76 |
| UD1100 | 57 | Male | Control |  | 30.42 | 43 |
| UC400 | 89 | Male | Control |  | 30.47 | 43 |
| UC384 | 51 | Female | Control |  | 30.48 | 36.88 |
| UC495 | 62 | Male | Control |  | 30.52 | 40 |
| UD0816 | 50 | Male | Control |  | 30.56 | 43 |
| UD0990 | 56 | Female | Control |  | 30.57 | 40.11 |
| UD0556 | 56 | Male | Control |  | 30.61 | 43 |
| UD0777 | 57 | Female | Control |  | 30.61 | 43 |
| UC131 | 55 | Female | Control |  | 30.61 | 43 |
| UD0423 | 62 | Male | Control |  | 30.63 | 43 |
| UD1014 | 67 | Male | Control |  | 30.68 | 43 |
| UD0262 | 52 | Female | Control |  | 30.71 | 43 |
| UC099 | 45 | Male | Control |  | 30.77 | 43 |
| UD0420 | 63 | Male | Control |  | 30.83 | 40 |
| UD0920 | 53 | Male | Control |  | 30.86 | 40.09 |
| UC298 | 69 | Male | Control |  | 30.88 | 40 |
| UD0573 | 56 | Male | Control |  | 31.01 | 43 |
| UD0657 | 45 | Female | Control |  | 31.09 | 43 |
| UD0337 | 58 | Female | Control |  | 31.09 | 39.38 |
| UD1070 | 67 | Female | Control |  | 31.14 | 33.12 |
| UD0309 | 47 | Male | Control |  | 31.21 | 43 |
| UD0327 | 40 | Female | Control |  | 31.22 | 40 |
| UC170 | 74 | Female | Control |  | 31.71 | 37.22 |
| UA291 | 56 | Female | Control |  | 31.76 | 43 |
| UC072 | 62 | Male | Control |  | 31.77 | 43 |
| UD1041 | 68 | Male | Control |  | 31.79 | 43 |
| UD0264 | 41 | Male | Control |  | 31.79 | 43 |
| UD0575 | 61 | Male | Control |  | 31.8 | 43 |
| UC450 | 55 | Female | Control |  | 31.84 | 43 |
| UD0970 | 62 | Male | Control |  | 32.13 | 43 |
| UD0877 | 59 | Male | Control |  | 32.14 | 43 |
| UA210 | 65 | Male | Control |  | 32.28 | 43 |
| UC038 | 51 | Male | Control |  | 32.46 | 43 |
| UC462 | 58 | Female | Control |  | 32.61 | 43 |
| UD0837 | 57 | Female | Control |  | 32.62 | 43 |
| UD0148 | 40 | Male | Control |  | 32.67 | 43 |
| UD1021 | 58 | Male | Control |  | 32.68 | 43 |
| UD0874 | 73 | Male | Control |  | 32.73 | 43 |
| UC431 | 70 | Female | Control |  | 32.9 | 43 |
| UD0320 | 63 | Male | Control |  | 33.03 | 43 |
| UD0643 | 63 | Male | Control |  | 33.45 | 43 |
| UA101 | 71 | Male | Control |  | 33.63 | 43 |
| UD0896 | 63 | Male | Control |  | 33.69 | 43 |
| UC452 | 58 | Female | Control |  | 33.7 | 43 |
| UD0445 | 51 | Female | Control |  | 33.79 | 43 |
| UD0658 | 71 | Female | Control |  | 33.79 | 43 |
| UD0955 | 51 | Male | Control |  | 34.31 | 43 |
| UC053 | 64 | Female | Control |  | 34.67 | 43 |
| UD1076 | 66 | Male | Control |  | 34.87 | 43 |
| UC445 | 66 | Male | Benign tumors of bladder | NA | 28.8 | 30.49 |
| UC179 | 55 | Male | Benign tumors of bladder | NA | 28.01 | 31.93 |
| UD1025 | 79 | Male | Benign tumors of bladder | NA | 28.51 | 32.54 |
| UD1078 | 69 | Male | Benign tumors of bladder | NA | 28.27 | 32.61 |
| UC183 | 65 | Male | Benign tumors of bladder | NA | 28.52 | 32.99 |
| UD1069 | 77 | Male | Benign tumors of bladder | NA | 28.42 | 33.74 |
| UC245 | 47 | Male | Benign tumors of bladder | NA | 28.24 | 36.43 |
| UC257 | 56 | Male | Benign tumors of bladder | NA | 29.06 | 37.81 |
| UA001 | 47 | Male | Benign tumors of bladder | NA | 28.78 | 37.92 |
| UA310 | 61 | Male | Benign tumors of bladder | NA | 29.5 | 39.5 |
| UA126 | 25 | Female | Benign tumors of bladder | NA | 28.48 | 40 |
| UD1018 | 44 | Male | Benign tumors of bladder | NA | 28.57 | 40 |
| UA214 | 56 | Male | Benign tumors of bladder | NA | 28.8 | 40 |
| UC317 | 59 | Male | Benign tumors of bladder | NA | 29.93 | 40 |
| UC329 | 47 | Female | Benign tumors of bladder | NA | 28.44 | 43 |
| UA232 | 51 | Male | Benign tumors of bladder | NA | 32.76 | 43 |
| UD1027 | 54 | Male | Benign tumors of bladder | NA | 28.6 | 43 |
| UD1060 | 56 | Male | Benign tumors of bladder | NA | 28.41 | 43 |
| UA222 | 66 | Female | Benign tumors of bladder | NA | 27.94 | 43 |
| UD1139 | 68 | Male | Benign tumors of bladder | NA | 28.55 | 43 |
| UD1116 | 71 | Male | Benign tumors of bladder | NA | 28.99 | 43 |
| UD1003 | 76 | Male | Benign tumors of bladder | NA | 28.83 | 43 |
| UC322 | 63 | Male | Recurring cancers | NA | 28.6 | 29.87 |
| UC486 | 64 | Male | Recurring cancers | NA | 28.69 | 29.02 |
| UA059 | 64 | Male | Recurring cancers | NA | 28.74 | 43 |
| UC407 | 67 | Male | Recurring cancers | NA | 28.58 | 32.26 |
| UD0598 | 68 | Male | Recurring cancers | T1N0M0 | 29.64 | 31.68 |
| UC478 | 70 | Female | Recurring cancers | NA | 28.16 | 29.51 |
| UD0769 | 71 | Male | Recurring cancers | T4aN0M0 | 28.74 | 30.88 |
| UC308 | 74 | Male | Recurring cancers | NA | 28.59 | 30.05 |
| UD1061 | 57 | Male | Prostate cancers | T2N0M0 | 34.83 | 43 |
| UD0940 | 61 | Male | Prostate cancers | NA | 31.73 | 43 |
| UA271 | 63 | Male | Prostate cancers | NA | 32.18 | 43 |
| UA242 | 64 | Male | Prostate cancers | pMIc | 29.44 | 38.09 |
| UC148 | 64 | Male | Prostate cancers | NA | 33.48 | 43 |
| UA205 | 65 | Male | Prostate cancers | T2cN0M0 | 28.7 | 38.21 |
| UA212 | 65 | Male | Prostate cancers | pT2N0M0 | 29.18 | 37.74 |
| UD0958 | 66 | Male | Prostate cancers | T3bN1M0 | 29 | 39.31 |
| UA233 | 67 | Male | Prostate cancers | pT2N0M0 | 28.05 | 40 |
| UD0972 | 68 | Male | Prostate cancers | T3aN0M0 | 27.93 | 43 |
| UC164 | 68 | Male | Prostate cancers | NA | 28.5 | 40 |
| UA299 | 68 | Male | Prostate cancers | NA | 28.58 | 36.73 |
| UD1050 | 68 | Male | Prostate cancers | T4N0M0 | 28.62 | 43 |
| UA256 | 69 | Male | Prostate cancers | T2cNxM1b | 28.26 | 35.04 |
| UA229 | 70 | Male | Prostate cancers | pT3bN0M0 | 29.74 | 40 |
| UA300 | 71 | Male | Prostate cancers | T2cN0M0 | 31.55 | 43 |
| UA235 | 72 | Male | Prostate cancers | NA | 28.11 | 38.04 |
| UD0982 | 72 | Male | Prostate cancers | T2N0M0 | 28.85 | 35.07 |
| UA209 | 73 | Male | Prostate cancers | pT2N0M0 | 29.14 | 43 |
| UC144 | 73 | Male | Prostate cancers | NA | 32.03 | 43 |
| UA249 | 75 | Male | Prostate cancers | pT2N0M0 | 27.99 | 40 |
| UC136 | 75 | Male | Prostate cancers | pT2N0M0 | 28.58 | 43 |
| UD0977 | 76 | Male | Prostate cancers | T3bN1M0 | 28.88 | 36.59 |
| UD0963 | 76 | Male | Prostate cancers | T2cN0M0 | 29.16 | 37.31 |
| UD1072 | 77 | Male | Prostate cancers | T4N1M0 | 28.41 | 38.05 |
| UD0949 | 77 | Male | Prostate cancers | T3bN1M0 | 29.75 | 40 |
| UD1036 | 77 | Male | Prostate cancers | T3bN0M0 | 31.03 | 43 |
| UD0952 | 78 | Male | Prostate cancers | NA | 31.47 | 43 |
| UA297 | 80 | Male | Prostate cancers | pMIc | 27.74 | 37.6 |
| UA225 | 80 | Male | Prostate cancers | pT2N0M0 | 28.92 | 38.42 |
| UA198 | 85 | Male | Prostate cancers | pT2N0M0 | 28.8 | 36.4 |
| UD0154 | 58 | Male | Prostate cancers | T4NxM0 | 27.92 | 40 |
| UD0100 | 60 | Male | Prostate cancers | T3bN0M0 | 31.45 | 40 |
| UD0117 | 60 | Male | Prostate cancers | T3bN0M0 | 32.78 | 43 |
| UD0204 | 63 | Male | Prostate cancers | TxN0M1b | 31.3 | 40 |
| UD0083 | 64 | Male | Prostate cancers | T3bN0M0 | 31.16 | 43 |
| UD0241 | 64 | Male | Prostate cancers | PT3aNx | 33.73 | 37.36 |
| UD0228 | 64 | Male | Prostate cancers | T2bN0M0 | 34.06 | 43 |
| UD0102 | 67 | Male | Prostate cancers | T3bN0M0 | 28.15 | 43 |
| UD0206 | 69 | Male | Prostate cancers | T2bN0M1b | 28.94 | 43 |
| UA197 | 69 | Male | Prostate cancers | NA | 28.95 | 40 |
| UD0025 | 69 | Male | Prostate cancers | NA | 29.54 | 38.83 |
| UD0138 | 71 | Male | Prostate cancers | T3bN0M0 | 27.94 | 40 |
| UA110 | 71 | Male | Prostate cancers | T3aN0M0 | 29.08 | 40 |
| UD0231 | 72 | Male | Prostate cancers | T3bN0M0 | 28.31 | 40 |
| UD0157 | 75 | Male | Prostate cancers | T3bN0M1a | 30.63 | 43 |
| UD0151 | 81 | Male | Prostate cancers | T3bN0M0 | 28.18 | 43 |
| UA107 | 82 | Male | Prostate cancers | T2cN0M0 | 28.31 | 38.3 |
| UD0165 | 82 | Male | Prostate cancers | T2cN0M0 | 28.95 | 43 |
| UD1066 | 55 | Female | Renal carcinomas | NA | 29.46 | 36.88 |
| UD0329 | 41 | Male | Renal carcinomas | T1aN0M0 | 30.52 | 43 |
| UA099 | 43 | Male | Renal carcinomas | NA | 28.17 | 35.62 |
| UA133 | 45 | Female | Renal carcinomas | NA | 30.08 | 43 |
| UD0198 | 47 | Female | Renal carcinomas | T1bN0M0 | 30.02 | 43 |
| UA151 | 50 | Male | Renal carcinomas | NA | 28.96 | 43 |
| UD0343 | 50 | Female | Renal carcinomas | T1bN0M0 | 29.32 | 43 |
| UA103 | 53 | Male | Renal carcinomas | T1bN0M0 | 28.3 | 38.87 |
| UD0187 | 53 | Male | Renal carcinomas | T1aN0M0 | 33.44 | 43 |
| UD0196 | 54 | Female | Renal carcinomas | T1aN0M0 | 30.65 | 43 |
| UD0185 | 54 | Female | Renal carcinomas | T1aN0M0 | 31.53 | 43 |
| UD0317 | 55 | Male | Renal carcinomas | T1aN0M0 | 30.96 | 43 |
| UD0131 | 55 | Male | Renal carcinomas | T2aN0M0 | 31.33 | 43 |
| UD0219 | 56 | Male | Renal carcinomas | T2aN0M1 | 28.29 | 40 |
| UA145 | 56 | Female | Renal carcinomas | T1aN0M0 | 28.95 | 40 |
| UD0096 | 59 | Male | Renal carcinomas | T3aN0M0 | 28.28 | 40 |
| UA128 | 60 | Male | Renal carcinomas | T1aN0M0 | 29.16 | 40 |
| UD0189 | 64 | Male | Renal carcinomas | T2aN0M0 | 28.19 | 43 |
| UC122 | 68 | Male | Renal carcinomas | T1bN0M0 | 28.45 | 43 |
| UD0227 | 69 | Male | Renal carcinomas | T1aN0M0 | 31.84 | 43 |
| UD0129 | 73 | Female | Renal carcinomas | T1bN0M0 | 28.16 | 39.09 |
| UD0191 | 74 | Male | Renal carcinomas | T2aN0M0 | 28.27 | 40 |
| UC116 | 77 | Male | Renal carcinomas | NA | 28.21 | 38.08 |
| UA155 | 78 | Male | Renal carcinomas | T2aN0M0 | 32.94 | 43 |
| UD1009 | 44 | Male | Renal carcinomas | T2aN0M0 | 28.49 | 43 |
| UD1054 | 46 | Male | Renal carcinomas | T1aN0M0 | 29.21 | 40 |
| UD1046 | 52 | Female | Renal carcinomas | T1aN0M0 | 29.54 | 43 |
| UD1043 | 54 | Male | Renal carcinomas | T1bN0M0 | 28.8 | 40 |
| UD1049 | 56 | Male | Renal carcinomas | NA | 30.6 | 43 |
| UD1063 | 57 | Female | Renal carcinomas | T2aN0M0 | 30.81 | 39.37 |
| UD1144 | 59 | Female | Renal carcinomas | T2aN0M0 | 28.51 | 39.44 |
| UD1120 | 62 | Female | Renal carcinomas | T1aN0M0 | 28.55 | 38.64 |
| UD1113 | 62 | Male | Renal carcinomas | T2aN0M0 | 28.66 | 38.53 |
| UD1020 | 62 | Male | Renal carcinomas | T1bN0M0 | 32.52 | 43 |
| UD1084 | 65 | Male | Renal carcinomas | T1aN0M0 | 29.02 | 43 |
| UD1062 | 65 | Female | Renal carcinomas | T1bN0M0 | 30.84 | 43 |
| UD1108 | 65 | Male | Renal carcinomas | T1bN0M0 | 32.5 | 43 |
| UD1024 | 66 | Male | Renal carcinomas | T1bN0M0 | 31.45 | 43 |
| UD1130 | 67 | Male | Renal carcinomas | T1aN0M0 | 30.96 | 43 |
| UD1033 | 75 | Male | Renal carcinomas | T1bN0M0 | 30.88 | 38.24 |
| UD0900 | 52 | Male | Carcinomas of renal pelvis | T1N0M0 | 27.87 | 35.15 |
| UD0906 | 57 | Female | Carcinomas of renal pelvis | T2N0M0 | 28.12 | 31.21 |
| UD0921 | 62 | Female | Carcinomas of renal pelvis | T3N0M0 | 27.77 | 33.34 |
| UD1052 | 66 | Male | Carcinomas of renal pelvis | T3N0M0 | 28.21 | 31.06 |
| UD1065 | 66 | Male | Carcinomas of renal pelvis | T3N0M0 | 30.49 | 32.32 |
| UA234 | 68 | Female | Carcinomas of renal pelvis | T3N0M0 | 28.03 | 35.94 |
| UD1017 | 70 | Female | Carcinomas of renal pelvis | T3N0M0 | 28.59 | 30.03 |
| UC267 | 74 | Female | Carcinomas of renal pelvis | NA | 30.62 | 37.15 |
| UD0905 | 76 | Female | Carcinomas of renal pelvis | T3N0M0 | 28.45 | 35.21 |
| UD1005 | 76 | Male | Carcinomas of renal pelvis | T1N0M0 | 28.93 | 32.04 |
| UD0615 | 77 | Female | Carcinomas of renal pelvis | T2N0M0 | 28.58 | 32.13 |
| UD1010 | 84 | Female | Carcinomas of renal pelvis | T1N0M0 | 28.31 | 29.88 |
| UD0273 | 45 | Female | Carcinomas of ureter | NA | 28.87 | 43 |
| UD0944 | 52 | Male | Carcinomas of ureter | T1N0M0 | 28.44 | 31.11 |
| UD0947 | 68 | Female | Carcinomas of ureter | T1N0M0 | 28.31 | 36.73 |
| UD1037 | 68 | Male | Carcinomas of ureter | T2N0M0 | 28.71 | 30.2 |
| UA246 | 70 | Female | Carcinomas of ureter | T1N0M0 | 28.3 | 37.84 |
| UD1148 | 83 | Male | Carcinomas of ureter | T1N0M0 | 28.46 | 37.98 |

*****Reference gene is *ACTB*.

**Supplementary Table S11. Association of urine methylated *DMRTA2* with clinical characteristics**

|  | *DMRTA2* methylation in urine samples | | | |
| --- | --- | --- | --- | --- |
|  |  | (+) | (-) | P |
| **Total** | **216** |  |  |  |
| **Age** | <60 y | 29 | 15 | <0.001 |
|  | ≥60 y | 150 | 22 |  |
| **Sex** | Male | 152 | 27 | 0.56 |
|  | Female | 30 | 7 |  |
| **Stage** | Ta | 34 | 20 | <0.001 |
|  | T1/T2 | 104 | 9 |  |
|  | T3/T4 | 18 | 4 |  |
| **Grade** | low | 60 | 24 | <0.001 |
|  | High | 106 | 6 |  |

**Supplementary Table S12. Levels of *DMRTA2* mRNA expression in nine BC cell lines and one bladder epithelial cell line after 5’-Aza-dC treatment (demethylation)**

| Cell Line | MOCK | 5-AZA (*‾X ± S* ) |
| --- | --- | --- |
| SV-HUC-1 | 1.0000 | 58.43±26.20 |
| 5637 | 1.0000 | 2.25±0.96 |
| BIU-87 | 1.0000 | 33.02±12.50 |
| T24 | 1.0000 | 80.01±31.04 |
| SCaBER | 1.0000 | 2.46±0.82 |
| TCCSUP | 1.0000 | 1.38±0.198 |
| J82 | 1.0000 | 1.638±0.34 |
| UM-UC-3 | 1.0000 | 1.60±1.12 |
| SW780 | 1.0000 | 2.25±0.88 |
| RT4 | 1.0000 | 69.27±25.88 |

**Supplementary Table S13. DMRTA2 expression in tissue specimens revealed by IHC staining.**

|  | No staining | Weak staining | Moderate staining | Strong staining |
| --- | --- | --- | --- | --- |
|  | <10% | 11%–50% | 51%–80% | >80% |
| Normal (n = 11) | 11(<10%) | 0 | 0 | 0 |
| Carcinoma (n = 19) | 0 | 16 (11%–50%) | 3 (51%–80%) | 0 |

**Supplementary References**

[1] Ji, M., Guan, H., Gao, C., Shi, B. & Hou, P. Highly frequent promoter methylation and PIK3CA amplification in non-small cell lung cancer (NSCLC). BMC Cancer 2011;11:147.

[2] Yu, J. et al. A novel set of DNA methylation markers in urine sediments for sensitive/specific detection of bladder cancer. Clin Cancer Res. 2007;13:7296–304.

[3] Dulaimi, E., Uzzo, R. G., Greenberg, R. E., Al-Saleem, T. & Cairns, P. Detection of bladder cancer in urine by a tumor suppressor gene hypermethylation panel. Clin Cancer Res. 2004;10: 1887–93 (2004).

[4] Hoque, M. O. et al. Quantitation of promoter methylation of multiple genes in urine DNA and bladder cancer detection. J Natl Cancer Inst. 2006;98:996–1004.

[5] Dulaimi, E. et al. Promoter hypermethylation profile of kidney cancer. Clin Cancer Res. 2004:10: 3972–9.

[6] Ahmed, H. Promoter methylation in prostate cancer and its application for the early detection of prostate cancer using serum and urine samples. Biomark Cancer 2010;2:17–33.

[7] Scher, M. B. et al. Detecting DNA methylation of the BCL2, CDKN2A and NID2 genes in urine using a nested methylation specific polymerase chain reaction assay to predict bladder cancer. J Urol. 2012;188:2101–7.

[8] Friedrich, M. G. et al. Detection of methylated apoptosis-associated genes in urine sediments of bladder cancer patients. Clin Cancer Res. 2004;10:7457–65.

[9] Gurioli, G. et al. Methylation pattern analysis in prostate cancer tissue: identification of biomarkers using an MS-MLPA approach. J Transl Med. 2016;14:249.

[10] Brait, M. et al. Aberrant promoter methylation of multiple genes during pathogenesis of bladder cancer. Cancer Epidemiol Biomarkers Prev. 2008;17:2786–94.

[11] Chan, M. W. Y. et al. Hypermethylation of multiple genes in tumor tissues and voided urine in urinary bladder cancer patients. Clin Cancer Res. 2002;8:464–70.

[12] Nojima, D. et al. CpG methylation of promoter region inactivates E-cadherin gene in renal cell carcinoma. Mol Carcinog. 2001;32:19–27.

[13] Onay, H., Pehlivan, S., Koyuncuoglu, M., Kirkali, Z. & Ozkinay, F. Multigene methylation analysis of conventional renal cell carcinoma. Urol Int. 2009;83:107–12.

[14] Shenoy, N. et al. Role of DNA methylation in renal cell carcinoma. J Hematol Oncol. 2015;8:88.

[15] Christoph, F. et al. Methylation of tumour suppressor genes APAF-1 and DAPK-1 and in vitro effects of demethylating agents in bladder and kidney cancer. Br J Cancer. 2006;95:1701–7.

[16] Morris, M. R. et al. Identification of candidate tumour suppressor genes frequently methylated in renal cell carcinoma. Oncogene 2010;29:2104–17.

[17] van Vlodrop, I. J. H. et al. Prognostic significance of Gremlin1 (GREM1) promoter CpG island hypermethylation in clear cell renal cell carcinoma. Am J Pathol. 2010;176:575–84.

[18] Jerónimo, C. et al. Quantitation of GSTP1 methylation in non-neoplastic prostatic tissue and organ-confined prostate adenocarcinoma. J Natl Cancer Inst. 2001;93:1747–52.

[19] Costa, V. L. et al. Three epigenetic biomarkers, GDF15, TMEFF2, and VIM, accurately predict bladder cancer from DNA-based analyses of urine samples. Clin Cancer Res. 2010;16:5842–51.

[20] Allory, Y. et al. Telomerase reverse transcriptase promoter mutations in bladder cancer: high frequency across stages, detection in urine, and lack of association with outcome. Eur Urol. 2014;65: 360–6.

[21] Wang, L. et al. The Silencing of CCND2 by Promoter Aberrant Methylation in Renal Cell Cancer and Analysis of the Correlation between CCND2 Methylation Status and Clinical Features. PLoS One 2016;11:e0161859.

[22] Urakami, S. et al. Combination analysis of hypermethylated Wnt-antagonist family genes as a novel epigenetic biomarker panel for bladder cancer detection. Clin Cancer Res. 2006;12:2109–16.

[23] Kvasha, S. et al. Hypermethylation of the 5’CpG island of the FHIT gene in clear cell renal carcinomas. Cancer Lett. 2008;265:250–7.

[24] Eggers, H. et al. Prognostic and diagnostic relevance of hypermethylated in cancer 1 (HIC1) CpG island methylation in renal cell carcinoma. Int J Oncol. 2012;40:1650–8.

[25] Reinert, T. et al. Diagnosis of bladder cancer recurrence based on urinary levels of EOMES, HOXA9, POU4F2, TWIST1, VIM, and ZNF154 hypermethylation. PLoS One 2012;7:e46297.

[26] Dalgin, G. S. et al. Identification of novel epigenetic markers for clear cell renal cell carcinoma. J Urol. 2008;180:1126–30.

[27] Chen, P.-C. et al. Distinct DNA methylation epigenotypes in bladder cancer from different Chinese sub-populations and its implication in cancer detection using voided urine. BMC Med Genomics 2011;4:45.

[28] Kawamoto, K. et al. DNA methylation and histone modifications cause silencing of Wnt antagonist gene in human renal cell carcinoma cell lines. Int J Cancer 2008;123:535–42.

[29] Costa, V. L. et al. TCF21 and PCDH17 methylation: An innovative panel of biomarkers for a simultaneous detection of urological cancers. Epigenetics 2011;6:1120–30.

[30] Xin, J. et al. Clinical potential of TCF21 methylation in the diagnosis of renal cell carcinoma. Oncol Lett. 2016;12:1265–70.

[31] Kawakami, K. et al. Functional significance of Wnt inhibitory factor-1 gene in kidney cancer. Cancer Res. 2009;69:8603–10.

[32] Chung, W. et al. Detection of bladder cancer using novel DNA methylation biomarkers in urine sediments. Cancer Epidemiol Biomarkers Prev. 2011;20:1483–91.

[33] Wei, J.-H. et al. A CpG-methylation-based assay to predict survival in clear cell renal cell carcinoma. Nat Commun. 2015;6:8699.

[34] Morris, M. R. et al. Multigene methylation analysis of Wilms’ tumour and adult renal cell carcinoma. Oncogene 2003;22:6794–801.

[35] Du, Z. et al. The epigenetic modifier CHD5 functions as a novel tumor suppressor for renal cell carcinoma and is predominantly inactivated by promoter CpG methylation. Oncotarget 2016;7: 21618–30.

[36] Goltz, D. et al. CXCL12 promoter methylation and PD-L1 expression as prognostic biomarkers in prostate cancer patients. Oncotarget 2016;7:53309–20.

[37] Wang, Z.-R. et al. Validation of DAB2IP methylation and its relative significance in predicting outcome in renal cell carcinoma. Oncotarget 1016;7:31508–19.

[38] Hirata, H. et al. Wnt antagonist DKK1 acts as a tumor suppressor gene that induces apoptosis and inhibits proliferation in human renal cell carcinoma. Int J Cancer 2011;128:1793–803.

[39] Zhang, Q. et al. Aberrant methylation of the 8p22 tumor suppressor gene DLC1 in renal cell carcinoma. Cancer Lett. 2007;249:220–6.

[40] Kompier, L. C. et al. FGFR3, HRAS, KRAS, NRAS and PIK3CA mutations in bladder cancer and their potential as biomarkers for surveillance and therapy. PLoS One 2010;5:e13821.

[41] Cooper, S. J. et al. Loss of type III transforming growth factor-beta receptor expression is due to methylation silencing of the transcription factor GATA3 in renal cell carcinoma. Oncogene 2010;29: 2905–15.

[42] Peters, I. et al. GATA5 CpG island methylation in renal cell cancer: a potential biomarker for metastasis and disease progression. BJU Int. 2012;110:E144-152.

[43] Yoo, K. H., Park, Y.-K., Kim, H.-S., Jung, W.-W. & Chang, S.-G. Epigenetic inactivation of HOXA5 and MSH2 gene in clear cell renal cell carcinoma. Pathol Int. 2010;60:661–6.

[44] Wu, Y. et al. Methylation profiling identified novel differentially methylated markers including OPCML and FLRT2 in prostate cancer. Epigenetics 2016;11:247–58.

[45] Luan, Z. M., Zhang, H. & Qu, X. L. Prediction efficiency of PITX2 DNA methylation for prostate cancer survival. Genet Mol Res. 2016;15(2).

[46] Cancer Genome Atlas Research Network. Comprehensive molecular characterization of clear cell renal cell carcinoma. Nature 2013;499:43–9.

[47] Kawakami, K. et al. Secreted frizzled-related protein-5 is epigenetically downregulated and functions as a tumor suppressor in kidney cancer. Int J Cancer 2011;128:541–50.

[48] Zhao, X., Jen, J. & Peikert, T. Selection of Sensitive Methylation Markers for the Detection of Non-small Cell Lung Cancer. Journal of Molecular Biomarkers and Diagnosis 2015;06.

**Supplementary Images of Original Gels and Blots**

**Figure 4A：MSP**

Original unprocessed electrophoresis gel images：

Note：SK-N-SH and SK-N-BE(2) are neuroblastoma cell lines excluded from Figure 4A.

**Figure 4E: DMRTA2 protein expression**

**1--1** is the original unprocessed chemiluminescence image for GAPDH (**panel 1** in Figure 4E). **1-2** is the original unprocessed colorimetric composite image to show MW markers in the blot.

**2-1** is the orignal unprocessed chemiluminescence image for DMRTA2 (**panel 2** in Figure 4E). **2-2** is the original unprocessed colorimetric composite image to show MW markers in the blot.

Note: SK-N-SH and SK-N-BE(2) are neuroblastoma cell lines and excluded from Figure 4E.

**Figure 4G: DMRTA2 protein expression after 5’-Aza-dC treatment (demethylation)**

**3-1** is the original unprocessed chemiluminescence image for GAPDH (**panel 3** in Figure 4G). **3-2** is the original unprocessed colorimetric composite image to show MW markers in the blot.

**4-1** is the original unprocessed chemiluminescence image for DMRTA2 (**panel 4** in Figure 4G). **4-2** is the original unprocessed colorimetric composite image to show MW markers in the blot.

**5-1** is the original unprocessed chemiluminescence image for GAPDH (**panel 5** in Figure 4G). **5-2** is the original unprocessed colorimetric composite image to show MW markers in the blot.

Note: samples labeled with “other proteins” are unrelated to bladder cancer cell lines in Figure 4G.

**6-1** is the original unprocessed chemiluminescence image for DMRTA2 (**panel 6** in Figure 4G). **6-2** is the original unprocessed colorimetric composite image to show MW markers in the blot.

**7--1** is the original unprocessed chemiluminescence image for GAPDH (**panel 7** in Figure 4G). **7-2** is the original unprocessed colorimetric composite image to show MW markers in the blot.

**8-1** is the original unprocessed chemiluminescence image for DMRTA2 (**panel 8** in Figure 4G). **8-2** is the original unprocessed colorimetric composite image to show MW markers in the blot.
